# Supplementary material for: Cytosolic stress granules relieve the ubiquitin‐proteasome system in the nuclear compartment
Source: EMBO J. 2022 Dec 27;42(3):e111802. doi: 10.15252/embj.2022111802 (PMC9890234; doi:10.15252/embj.2022111802)
Supplement: Supplementary file 1 — Appendix [file EMBJ-42-e111802-s001.zip › 1.1.2.octet-stream]

**[External Email]**-Avoid clicking links or opening attachments unless you trust the sender.

  

Dear Team,

 

please find attached the files and the instructions below.

 

Thanks,

Annkatrin

 

**Von:** Hartmut Vodermaier, EMBOJ <h.vodermaier@embojournal.org>
  
**Gesendet:** Dienstag, 13. Dezember 2022 15:40  
**An:** The EMBO Journal <contact@embojournal.org>  
**Cc:** EMBO Press Production <embopressproduction@wiley.com>  
**Betreff:** Re: EMBJ 2022111802 - Appendix missing

 

|  |  |
| --- | --- |
| ⛔ | This is an external email. |

Dear Annkatrin,

Thanks again for flagging the missing Appendix file referenced on page 24 of this article.

I have now been in contact with the authors, and it was decided to replace this with an “Expanded View Computer Code” file.

I am attaching the file, combined with a legend text file, as ZIP - hope this is the correct format. 

The sentence on page 24,

> "The CellProfiler pipeline is available in the Appendix Supplementary Methods."

should be replace with

“The CellProfiler pipeline is provided as Computer Code EV1.”

Best regards,

Hartmut

**Hartmut Vodermaier, PhD**

Senior Editor | *The EMBO Journal*

h.vodermaier@embojournal.org

*Visit us at* embojournal.org  
*Follow us on Twitter* @embojournal

*and Mastodon* @embojournal@sciencemastodon.com

*Sign up for content alerts at* embopress.org/alertsfeeds

---

The contents of this email and any attachments are confidential and intended only for the person or entity to whom it is addressed. If you are not the intended recipient, any use, review, distribution, reproduction or any action taken in reliance upon this
message is strictly prohibited. If you received this message in error, please immediately notify the sender and permanently delete all copies of the email and any attachments.  
Click here for translations of this disclaimer.


---
