# Supplementary material for: Cytosolic stress granules relieve the ubiquitin‐proteasome system in the nuclear compartment
Source: EMBO J. 2022 Dec 27;42(3):e111802. doi: 10.15252/embj.2022111802 (PMC9890234; doi:10.15252/embj.2022111802)
Supplement: Supplementary file 2 — Expanded View Figures PDF [file EMBJ-42-e111802-s003.pdf]

Expanded View Figures

Figure EV1. G3BP1/2 deficiency impairs the UPS in thermally stressed cells.

A MelJuSo cells expressing Ub-YFP transfected with control siRNA or G3BP1 and G3BP2 siRNA were either left untreated (— heat shock) or exposed to 43°C for 30 min and followed for 4 h after the heat shock (Recovery). Cell lysates were analyzed by immunoblot with G3BP1, G3BP2, and GAPDH antibodies. Quantification of the G3BP1 and G3BP2 band densities. Data represent the mean ± SD (n = 3 independent experiments, Student's unpaired t-test, $^{**}P < 0.01$).

B MelJuSo cells expressing Ub-YFP transfected with control siRNA or G3BP1 and G3BP2 siRNA were subjected to 43°C heat shock for 30 min and followed by automated high-content imaging every 10 min for 4 h. Representative images are shown. Scale bar is 20 μm.

C Fluorescence micrographs of MelJuSo cells expressing YFP-CL1, which were either left untreated (— heat shock) or exposed to 43°C for 30 min and followed for 4 h after the heat shock (HS+ 4 h Rec.). Scale bar is 10 μm.

D Representative images of MelJuSo expressing Ub-YFP, which were transfected with G3BP1/2 siRNA and either transfected with mCherry-C1, mCherry-G3BP1, mCherry-G3BP1F33W or mCherry-G3BP1ΔRGG plasmids and subjected to heat shock. Scale bar is 20 μm.

E Fluorescence micrographs of MelJuSo cells expressing GFP-ODC, which were either left untreated (— heat shock) or exposed to 43°C for 30 min and followed for 4 h after heat shock (HS+ 4 h Rec.). Scale bar is 20 μm.

F Quantification of (E) of > 500 cells per group. Normalized to siControl "- heat shock." The frequency and distribution of the relative fluorescent intensity per cell are shown as violin plots. The solid lines in each distribution represent the median, and dash lines represent the upper and lower interquartile range limits (n = 3 independent experiments, > 1,000 cells analyzed per condition).

 © 2022 The Authors

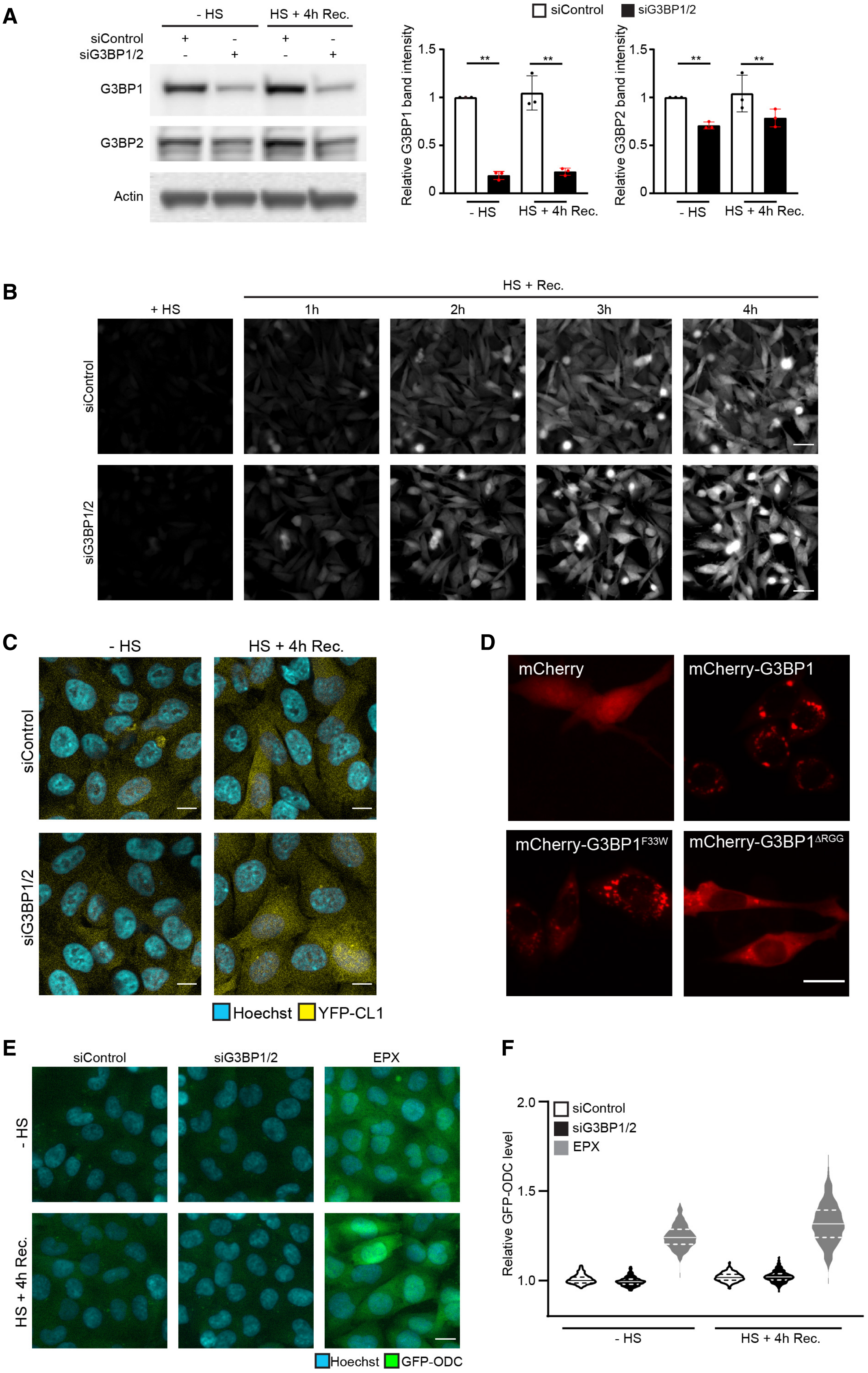
A
- HS
HS + 4h Rec.
siControl
siG3BP1/2
G3BP1
G3BP2
Actin
siControl
siG3BP1/2
Relative G3BP1 band intensity
Relative G3BP2 band intensity
- HS
HS + 4h Rec.
B
+ HS
HS + Rec.
1h
2h
3h
4h
siControl
siG3BP1/2
C
- HS
HS + 4h Rec.
siControl
siG3BP1/2
Hoechst
YFP-CL1
D
mCherry
mCherry-G3BP1
mCherry-G3BP1F33W
mCherry-G3BP1ΔRGG
E
siControl
siG3BP1/2
EPX
- HS
HS + 4h Rec.
Hoechst
GFP-ODC
F
siControl
siG3BP1/2
EPX
Relative GFP-ODC level
- HS
HS + 4h Rec.

Figure EV1.

Figure EV2. Nuclear UPS impairment and nucleolar DRiP accumulation in G3BP1/2 knockout cells.

A Flow cytometric detection of fluorescent intensities MelJuSo cells expressing NLS/NES-GFP-CL1, which were either left untreated (− HS), or exposed to 43°C for 30 min and followed for 4 h after heat shock (HS+ Rec.). Data represent the mean ± SD ($n = 3$ independent experiments, Student's unpaired t-test, *$P < 0.05$).

B Flow cytometric detection of fluorescent intensities in parental U2OS and G3BP1/2 knockout (KO) cells expressing Ub-YFP, which were either left untreated (− HS), or exposed to 43°C for 30 min and followed for 4 h after heat shock (Rec.). Data represent the mean ± SD ($n = 3$ independent experiments, Student's unpaired t-test, *$P < 0.05$).

C Fluorescence micrographs of parental U2OS and G3BP1/2 KO cells expressing Ub-YFP, which were either left untreated (− HS), exposed to 43°C for 30 min (HS), or followed for 4 h after heat shock (HS+ Recovery). Stress granules are visualized by immunostaining for TIA1. Scale bar is 10 μm.

D Representative confocal images of immunofluorescent staining of puromycin-labeled proteins in parental and G3BP1/2 KO U2OS cells exposed to a heat shock (+ HS; left panel), after 1 h recovery (HS + 1 h Rec.; middle panel), and after 2 h recovery (HS + 2 h Rec.; right panel). Scale bar is 10 μm.

E Quantification of nucleolar localization of puromycin labeled proteins in parental and G3BP1/2 KO U2OS cells in (D). Data represent the mean ± SD ($n = 3$ independent experiments, > 50 cells analyzed per condition, Kruskal-Wallis test, **$P < 0.01$, n.s.—not significant).

▶

 © 2022 The Authors

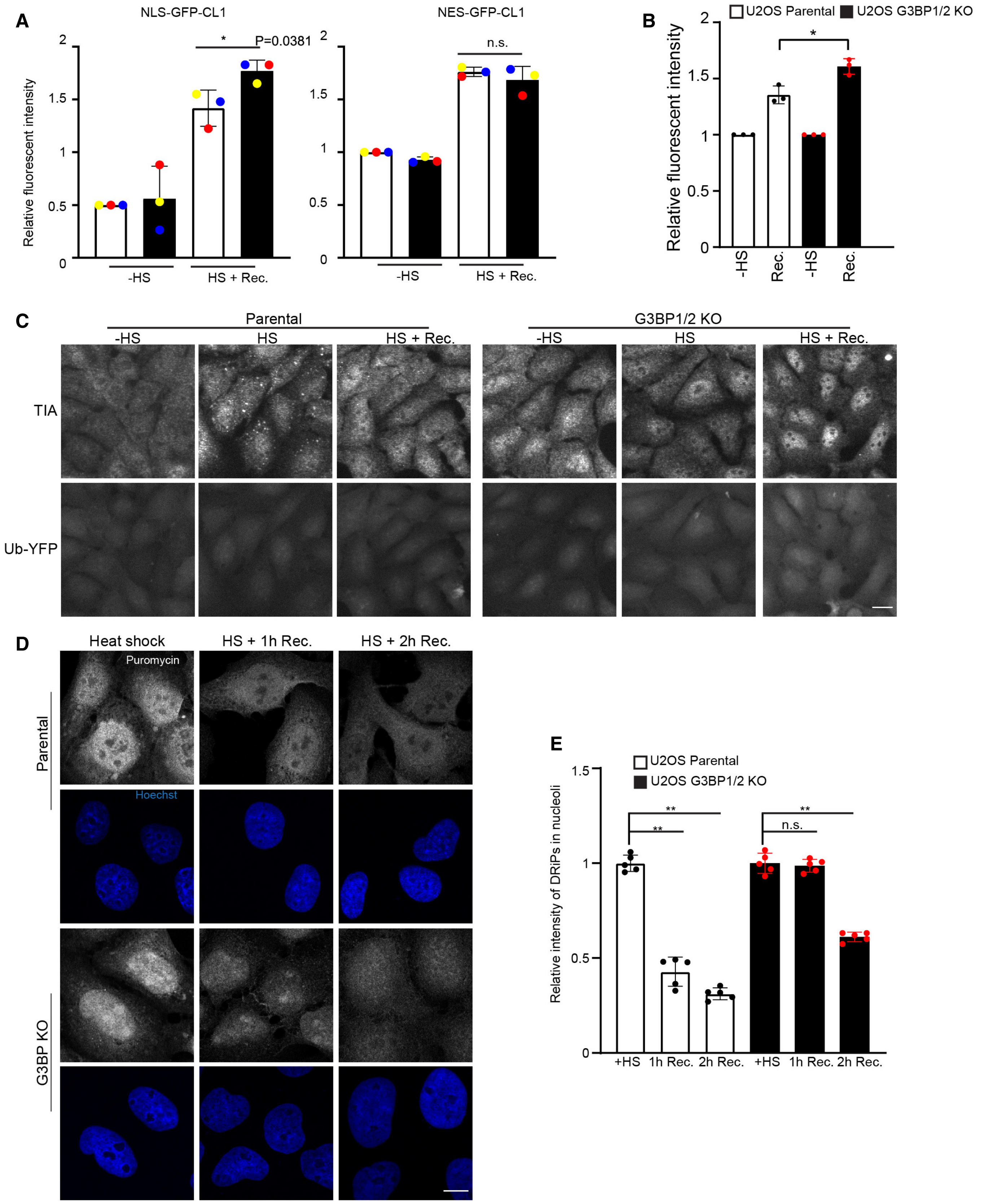
A
NLS-GFP-CL1
NES-GFP-CL1
Relative fluorescent intensity
*
P=0.0381
n.s.
-HS
HS + Rec.
B
U2OS Parental
U2OS G3BP1/2 KO
Relative fluorescent intensity
-HS
Rec.
C
Parental
G3BP1/2 KO
-HS
HS
HS + Rec.
TIA
Ub-YFP
D
Heat shock
HS + 1h Rec.
HS + 2h Rec.
Puromycin
Hoechst
Parental
G3BP KO
E
U2OS Parental
U2OS G3BP1/2 KO
Relative intensity of DRiPs in nucleoli
**
n.s.
+HS
1h Rec.
2h Rec.

Figure EV2.

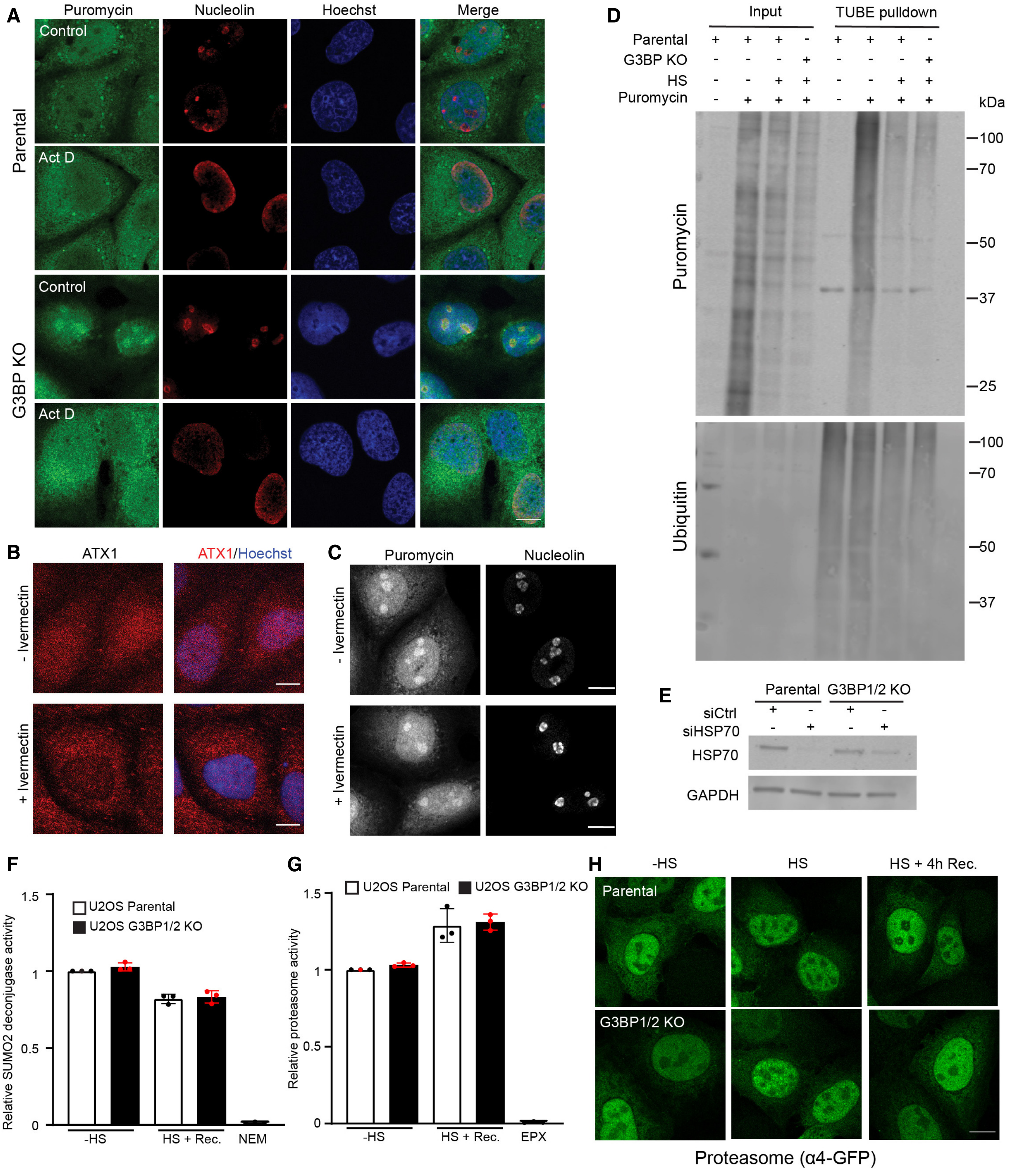

Figure EV3.

© 2022 The Authors

Figure EV3. DRiPs and proteasome activity in thermally stressed G3BP1/2 knockout cells.

A Representative confocal images of immunofluorescent staining of the nucleolar marker nucleolin and puromycin-labeled proteins in U2OS cells pretreated with or without 4 μM Actinomycin D for 3 h, subjected to a heat shock. Scale bar is 20 μm.

B U2OS G3BP1/2 knockout cells were treated with or without 25 μM ivermectin for 2 h. The ataxin1 was visualized by immunostaining. Scale bar is 20 μm.

C U2OS G3BP1/2 knockout cells were treated with or without 25 μM ivermectin for 2 h before being exposed to 43°C heat shock in a 5 μg/ml puromycin-containing medium. The DRiPs and nucleoli were visualized by immunostaining for puromycin and nucleolin, respectively. Scale bar is 20 μm.

D Immunoblot of ubiquitylated DRiPs by TUBE pulldown of lysates from parental U2OS and G3BP1/2 knockout cells after heat shock.

E Immunoblot of HSP70 in parental and G3BP1/2 knockout U2OS cells transfected with HSP70 siRNA for 72 h.

F Parental U2OS and G3BP1/2 knockout cells were either left untreated (− HS), or exposed to 43°C for 30 min and followed for 4 h after heat shock (HS + Rec.). The SUMO2 deconjugase activity was detected by following the conversion of the fluorogenic SUMO2- AMC substrate over 1 h. As a control, 20 mM NEM was added to the reaction mixture to inhibit SUMO2 deconjugases. Data represent the mean ± SD ($n = 3$ independent experiments, Student's unpaired t-test).

G Parental U2OS and G3BP1/2 knockout cells were either left untreated (− HS), or exposed to 43°C for 30 min and followed for 4 h after heat shock (HS + Rec.). The chymotrypsin-like activity (β5 subunit) of the proteasome was detected by following the conversion of the fluorogenic Suc-LLVY-AMC substrate over 1 h. As a control, 100 nM epoxomicin (EPX) was added to the reaction mixture to inhibit proteasome activity. Data represent the mean ± SD ($n = 3$ independent experiments, Student's unpaired t-test).

H Representative images of parental U2OS and G3BP1/2 knockout cells transiently transfected with α4-GFP, which were either left untreated (− HS), or exposed to 43°C for 30 min and followed for 4 h after heat shock (HS + 4 h Rec.). Scale bar is 20 μm.

© 2022 The Authors

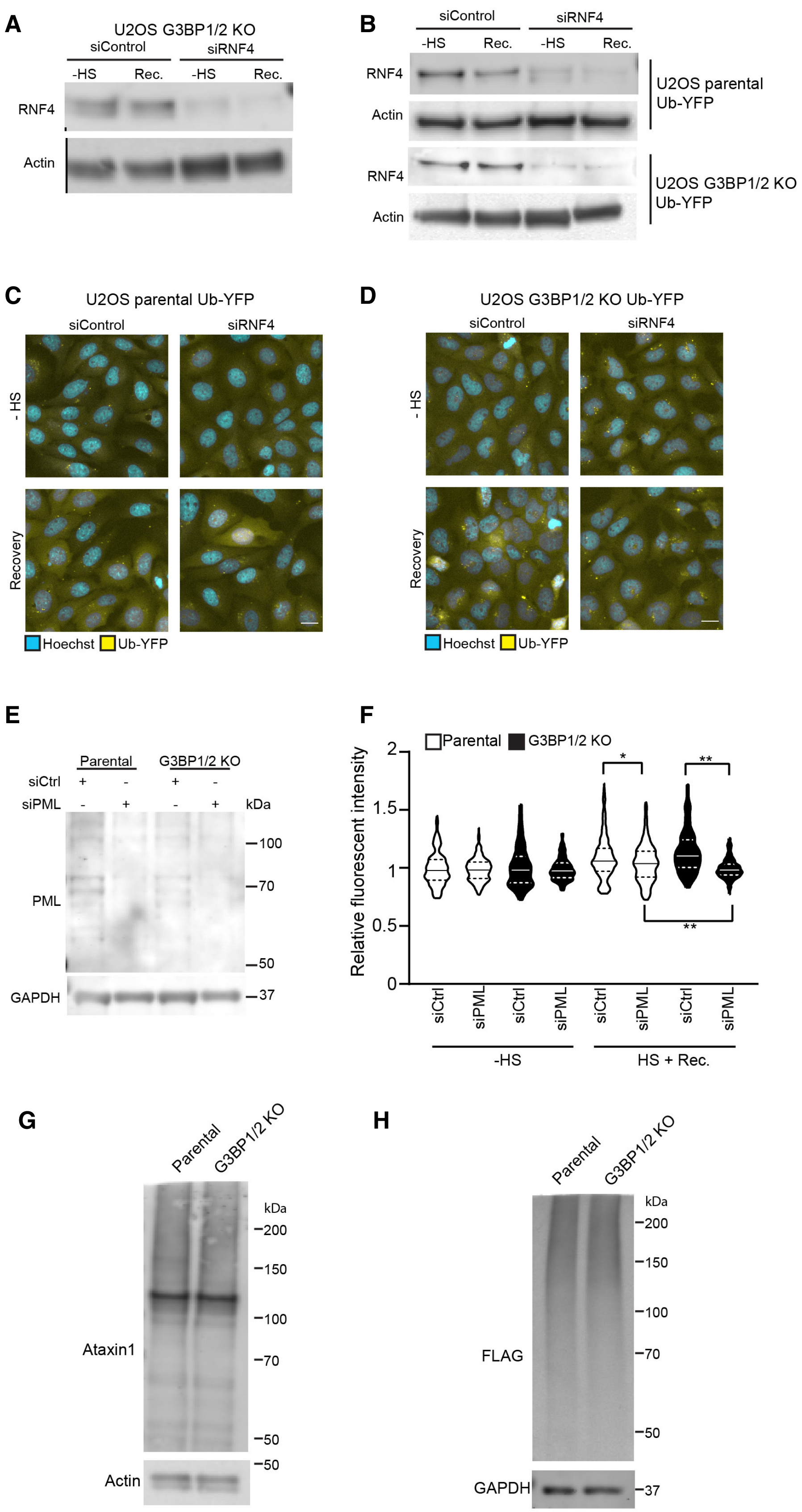

Figure EV4. Inhibition of SUMO-targeted ubiquitylation partially restores UPS activity in thermally stressed G3BP1/2 knockout cells.

A U2OS G3BP1/2 knockout cells expressing Ub-YFP were transfected with control siRNA or RNF4 siRNA. Cell lysates were analyzed by immunoblot with RNF4 and Actin antibodies.

B Parental U2OS and G3BP1/2 KO cells expressing Ub-YFP were transfected with control siRNA or RNF4 siRNA. Cell lysates were analyzed by immunoblot with RNF4 and Actin antibodies.

C Fluorescence micrographs of parental U2OS cells stably expressing Ub-YFP, which had been transfected with control or RNF4 siRNA. Cells were either left untreated (− heat shock), or exposed to 43°C for 30 min and followed for 4 h after heat shock (Recovery). Scale bar is 10 μm.

D Fluorescence micrographs of U2OS G3BP1/2 KO cells stably expressing Ub-YFP, which had been transfected with control or RNF4 siRNA. Cells were either left untreated (− heat shock), or exposed to 43°C for 30 min and followed for 4 h after heat shock (Recovery). Scale bar is 10 μm.

E Immunoblotting for PML in control (siControl) and PML-depleted (siPML) parental and G3BP1/2 KO U2OS cells.

F Quantification of mean cellular YFP fluorescence intensities in U2OS expressing Ub-YFP with and without PML siRNA transfection. The YFP fluorescence is normalized to untreated control cells. The frequency and distribution of the relative fluorescence intensities per cell are shown as violin plots. The solid lines in each distribution represent the median, and dash lines represent the upper and lower interquartile range limits ($n = 3$ independent experiments, > 1,000 cells analyzed per condition, Kruskal-Wallis test, *$P < 0.05$, **$P < 0.01$).

G Immunoblot of ataxin-1 in parental and G3BP1/2 KO U2OS cells transfected with ^{FLAG}Ub-Ataxin-1-82Q.

H Immunoblot of ^{FLAG}Ubiquitin in parental and G3BP1/2 KO U2OS cells transfected with ^{FLAG}Ub-Ataxin-1-82Q.

© 2022 The Authors
