# Supplementary material for: Cytosolic stress granules relieve the ubiquitin‐proteasome system in the nuclear compartment
Source: EMBO J. 2022 Dec 27;42(3):e111802. doi: 10.15252/embj.2022111802 (PMC9890234; doi:10.15252/embj.2022111802)
Supplement: Supplementary file 3 — PDF+ [file EMBJ-42-e111802-s002.pdf]

Article

TRANSPARENT PROCESS OPEN ACCESS

THE EMBO JOURNAL

Cytosolic stress granules relieve the ubiquitin-proteasome system in the nuclear compartment

Shanshan Xu[1], Maria E Gierisch[1], Anna Katharina Schellhaus[1], Ina Poser[2], Simon Alberti[2,3], Florian A Salomons[1] & Nico P Dantuma[1,*]

Abstract

The role of cytosolic stress granules in the integrated stress response has remained largely enigmatic. Here, we studied the functionality of the ubiquitin-proteasome system (UPS) in cells that were unable to form stress granules. Surprisingly, the inability of cells to form cytosolic stress granules had primarily a negative impact on the functionality of the nuclear UPS. While defective ribosome products (DRiPs) accumulated at stress granules in thermally stressed control cells, they localized to nucleoli in stress granule-deficient cells. The nuclear localization of DRiPs was accompanied by redistribution and enhanced degradation of SUMOylated proteins. Depletion of the SUMO-targeted ubiquitin ligase RNF4, which targets SUMOylated misfolded proteins for proteasomal degradation, largely restored the functionality of the UPS in the nuclear compartment in stress granule-deficient cells. Stress granule-deficient cells showed an increase in the formation of mutant ataxin-1 nuclear inclusions when exposed to thermal stress. Our data reveal that stress granules play an important role in the sequestration of cytosolic misfolded proteins, thereby preventing these proteins from accumulating in the nucleus, where they would otherwise infringe nuclear proteostasis.

Keywords protein quality control; proteostasis; stress granules; SUMO; ubiquitin-proteasome system
Subject Categories Post-translational Modifications & Proteolysis; Translation & Protein Quality
DOI 10.15252/embj.2022111802 | Received 1 June 2022 | Revised 25 November 2022 | Accepted 1 December 2022 | Published online 27 December 2022
The EMBO Journal (2023) 42: e111802

Introduction

Conditions that compromise protein homeostasis, that is, proteostasis, form a serious threat to cells as this can lead to cellular dysfunction and cell death unless acute measures are taken to restore the delicate equilibrium that allows proper functioning of the proteome (Sala *et al*, 2017). Mechanisms that safeguard proteostasis are adapted and rewired in the face of proteotoxic stress in order to avoid the accumulation of aberrant proteins that may otherwise pollute the intracellular environment with insoluble aggregates (Sherman & Goldberg, 2001). Reducing protein synthesis, optimizing protein folding, and accelerating the degradation of misfolded proteins are all measures that in a cooperative fashion promote proteostasis during cellular stress.

Misfolded or otherwise aberrant proteins are primarily degraded by the ubiquitin-proteasome system (UPS) (Kwon & Ciechanover, 2017). As such, this complex proteolytic system plays a critical role in the maintenance of proteostasis in the nuclear and cytosolic compartments of cells. Interception of misfolded proteins in the cytosol is facilitated by specific ubiquitin ligases that associate with molecular chaperones, such as Hsp70/HSPA1A, and ubiquitylate their client proteins (Shao & Hegde, 2016). In the nuclear compartment, the SUMO-targeted ubiquitin ligase RNF4 plays a central role in facilitating the degradation of faulty proteins (Guo *et al*, 2014), as it provides misfolded proteins that have initially been SUMOylated with ubiquitin chains that target these proteins for proteasomal degradation (Tatham *et al*, 2008). Importantly, the UPS also governs cellular processes unrelated to protein quality control, some of which are directly relevant to the coordination of the stress response, through the temporally and/or spatially regulated destruction of key regulators (Dang *et al*, 2021). Hence, a saturation of the UPS with misfolded proteins possesses a threat to cells as this may compromise other functions of the UPS that are vital for coping with proteotoxic stress and maintaining cell viability.

A final resort when misfolded proteins accumulate despite these countermeasures is their sequestration at allocated, subcellular locations. The most prominent example is the cytosolic inclusion body known as the aggresome, where in a coordinated and dynamic process, protein aggregates are transiently stored and kept from doing further harm (Kopito, 2000). While these protein deposits may not bring final relieve, they may provide the cell with a crucial time window to restore proteostasis and avoid a critical collapse caused

1 Department of Cell and Molecular Biology (CMB), Karolinska Institutet, Stockholm, Sweden
2 Max Planck Institute of Molecular Cell Biology and Genetics, Dresden, Germany
3 Biotechnology Center (BIOTEC), Center for Molecular and Cellular Bioengineering (CMCB), Technische Universität Dresden, Dresden, Germany
*Corresponding author. Tel: +46 8 52487384; E-mail: nico.dantuma@ki.se

© 2022 The Authors. Published under the terms of the CC BY 4.0 license.

by misfolded proteins overwhelming the folding and degradation systems (Arrasate *et al*, 2004). In addition to aggresomes, also other subcellular structures are involved in the sequestration of misfolded and/or aggregation-prone proteins, such as stress granules and nucleoli in the cytosolic and nuclear compartments, respectively (Nollen *et al*, 2001; Mateju *et al*, 2017; Frottin *et al*, 2019).

Stress granules are membrane-less, stress-induced structures that are part of the integrated stress response (Protter & Parker, 2016). These cytosolic structures are primarily composed of RNA molecules and RNA-binding proteins, which under proteotoxic stress conditions that induce global stalling of protein translation coalesce by liquid–liquid phase separation into small granules. Untranslated mRNAs are kept temporarily in a dormant state in stress granules until protein translation can be resumed upon which these structures will be rapidly resolved. In addition to their function as a temporary storage for RNA molecules that are not engaged in protein synthesis, it has been shown that cytosolic misfolded proteins also localize to stress granules (Ganassi *et al*, 2016; Mateju *et al*, 2017). This suggests that stress granules behave as a reservoir for misfolded proteins during the acute phase of the stress response.

In the nucleus, nucleoli have been identified as preferred sites for the sequestration of misfolded proteins (Nollen *et al*, 2001). These nuclear structures do not only share this functional feature with stress granules but also equally rely on liquid–liquid phase separation for their formation (Molliex *et al*, 2015; Mediani *et al*, 2019a), which implies that similar fundamental physical mechanisms may be in play for the coalescence of misfolded proteins at these locations (Frottin *et al*, 2019). This is supported by the finding that nucleoli can function as an overflow compartment for cytosolic aberrant proteins (Mediani *et al*, 2019a). Another similarity between stress granules and nucleoli is their dependence on the ubiquitin-like modifier SUMO for their structural integrity (Nacerddine *et al*, 2005; Marmor-Kollet *et al*, 2020). As SUMO also regulates the clearance of misfolded proteins by priming them for degradation through SUMO-targeted ubiquitylation (Guo *et al*, 2014), this ubiquitin-like modifier plays a multilayered role in the stress response by regulating both the localization and destruction of misfolded proteins.

Sequestration of misfolded proteins has been suggested to be important in keeping the UPS operative during proteotoxic stress as in the absence of these sequestration mechanisms misfolded proteins may saturate the UPS and distort its function (Maynard *et al*, 2009; Ortega *et al*, 2010). Indeed, the accumulation of aggregation-prone proteins that are linked to neurodegenerative diseases can cause a global collapse of the UPS resulting in disturbance in the timely degradation of regulatory proteins, which compromises cellular functionality and can cause cell death (Bence *et al*, 2001; Bennett *et al*, 2005, 2007). It has been proposed that a dysfunctional UPS may contribute to the cellular pathology observed in neurodegenerative diseases that are characterized by the accumulation of misfolded proteins and the presence of intracellular inclusions, such as amyotrophic lateral sclerosis (ALS) and spinocerebellar ataxia type 1 (SCA-1) (Ciechanover & Brundin, 2003).

Here, we investigated whether the formation of stress granules in response to proteotoxic stress is important for the functionality of the UPS. In line with this hypothesis, we found that cells that were unable to form stress granules displayed aggravated UPS impairment when challenged with acute proteotoxic stress. Surprisingly, the inability to form these cytosolic stress structures had a predominant negative effect on nuclear UPS activity, leading to disturbances in nuclear proteostasis. Our analysis shows that nucleolar relocalization of cytosolic misfolded proteins in stress granule-deficient cells alters the stress response. We propose that stress granules contribute to the preservation of UPS activity by sequestration of misfolded proteins and revealed an unexpected connection between cytosolic and nuclear stress responses aimed at restoring proteostasis.

Results

Stress granule-deficient cells display aggravated impairment of the UPS

We have previously shown that short thermally induced proteotoxic stress is followed by a recovery phase during which there is a transient state of UPS impairment resulting in the accumulation of ubiquitylated proteins (Salomons *et al*, 2009). Mild heat shock is a condition that also induces the formation of cytosolic stress granules, which have been shown to sequester misfolded proteins (Ganassi *et al*, 2016). As sequestration of misfolded proteins may be important to avoid a collapse of the UPS under conditions of proteotoxic stress (Maynard *et al*, 2009; Ortega *et al*, 2010), we investigated whether the absence of stress granules would further compromise the functionality of the UPS in stressed cells. To this end, we depleted a human MelJuSo cell line stably expressing the UPS reporter substrate ubiquitin^{Gly76Val}-yellow fluorescent protein (Ub-YFP) (Menendez-Benito *et al*, 2005) from G3BP1 and G3BP2, two RNA-binding proteins that are critical for the formation of stress granules (Kedersha *et al*, 2016). We confirmed that G3BP1 and G3BP2 were depleted by siRNA transfection (Fig EV1A). Whereas thermally stressed control cells formed cytosolic stress granules, as evidenced by the cytosolic puncta staining for the stress granule marker TIA1, G3BP1/2-depleted cells did not form stress granules but instead showed a predominant nuclear granular TIA1 pattern (Fig 1A). Notably, G3BP1/2 depletion prevented the formation of stress granules but did not interfere with the global inhibition of protein translation in response to proteotoxic stress as illustrated by a similar reduced incorporation of puromycin in newly synthesized proteins in thermally stressed control and stress granule-deficient MelJuSo cells (Fig 1B).

As previously reported (Salomons *et al*, 2009), we detected an accumulation of the Ub-YFP reporter substrate in cells during the recovery phase after mild thermal stress (Fig 1C and D). Interestingly, Ub-YFP increased to significantly higher levels in response to the heat shock in cells that had been depleted of G3BP1/2, suggesting that UPS impairment was aggravated in stress granule-deficient cells (Fig 1C and D). Notably, G3BP1/2 depletion by itself did not cause an increase in Ub-YFP levels in control cells, consistent with the lack of a stress-induced action of G3BP1/2 being responsible for compromised UPS functionality. Following the kinetics of Ub-YFP levels in control and stress, granule-deficient cells confirmed a more dramatic accumulation of Ub-YFP during the recovery phase of the G3BP1/2-depleted cells (Figs 1E and EV1B). A similar phenomenon was observed in cells expressing the aggregation-prone reporter substrate YFP-CL1, carrying the CL1 degradation signal (Bence *et al*, 2001; Menendez-Benito *et al*, 2005), which accumulated to a larger extent in thermally stressed cells that had been depleted of G3BP1/2 (Figs 1F and EV1C).

 © 2022 The Authors

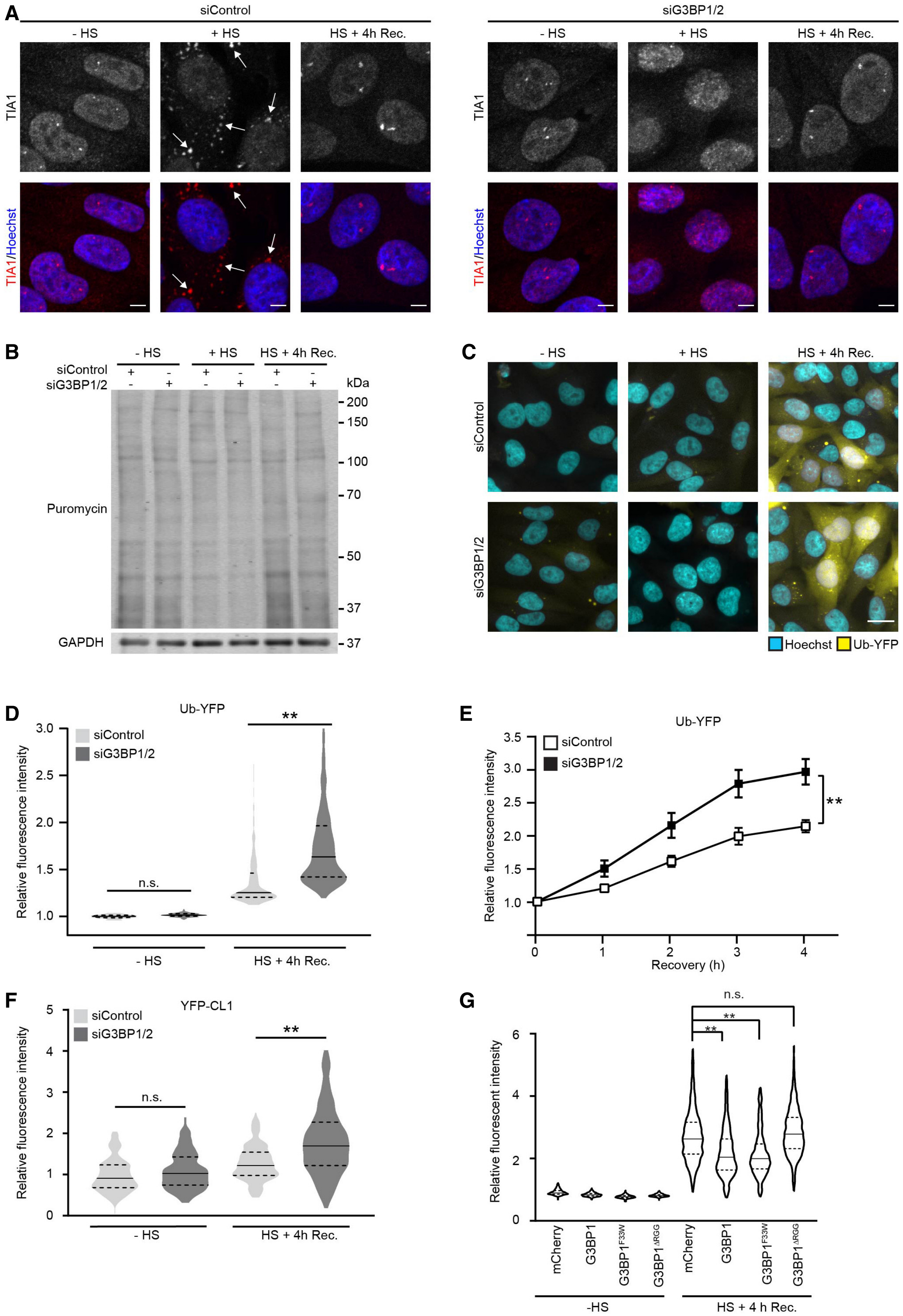
A
siControl
siG3BP1/2
- HS
+ HS
HS + 4h Rec.
TIA1
TIA1/Hoechst
B
siControl
siG3BP1/2
kDa
200
150
100
70
50
37
Puromycin
GAPDH
C
siControl
siG3BP1/2
Hoechst
Ub-YFP
D
Ub-YFP
Relative fluorescence intensity
n.s.
**
E
Ub-YFP
Recovery (h)
F
YFP-CL1
G
Relative fluorescent intensity
mCherry
G3BP1
G3BP1F33W
G3BP1ΔRGG
-HS
HS + 4 h Rec.

Figure 1.

◀ **Figure 1. Stress granule-deficient cells display aggravated impairment of the UPS.**

A Representative confocal images of immunofluorescent staining of the stress granule marker TIA1 in control (siControl, left panel) and G3BP1/2-depleted (siGRBP1/2, right panel) MelJuSo cells. Cells were left untreated (− HS), exposed to 43°C for 30 min (+ HS), and followed for 4 h after heat shock (HS + 4 h Rec.). Arrows indicate the stress granules in thermally stressed control cells. Scale bar, 10 µm.

B Analysis of puromycin incorporation in MelJuSo cells that were left untreated (− HS), exposed to 43°C for 30 min (+ HS), and followed for 4 h after heat shock (HS + 4 h Rec.). The blots were probed with antibodies against puromycin, and GAPDH as a loading control.

C Representative images of MelJuSo cells expressing Ub-YFP, which were either left untreated (− HS), or exposed to 43°C for 30 min (+ HS) and followed for 4 h after heat shock (HS + 4 h Rec.). Scale bar, 20 µm.

D Quantification of mean cellular YFP fluorescence intensities from the experiment shown in (C), normalized to untreated control cells (siControl - HS). The frequency and distribution of the relative fluorescence intensities per cell are shown as violin plots. The solid lines in each distribution represent the median, and dash lines represent the upper and lower interquartile range limits ($n = 3$ independent experiments, > 1,000 cells analyzed per condition, Kruskal-Wallis test, $^{**}P < 0.01$, n.s.—not significant).

E Kinetics of Ub-YFP image intensities in control (siControl) and stress granule-deficient MelJuSo cells (siG3BP1/2) in response to a 30 min 43°C heat shock, followed by 4 h at 37°C (Recovery). Error bars indicate the standard error of the mean ($n = 3$ independent experiments, 12 images analyzed per condition, Student's unpaired *t*-test, $^{**}P < 0.01$).

F Quantification of MelJuSo cells expressing YFP-CL1. Normalized to untreated control cells (siControl - HS). The frequency and distribution of the relative fluorescent intensity per cell are shown as violin plots. The solid lines in each distribution represent the median, and dash lines represent the upper and lower interquartile range limits ($n = 3$ independent experiments, > 1,000 cells analyzed per condition, Kruskal-Wallis test, $^{**}P < 0.01$, n.s.—not significant).

G Quantification of MelJuSo Ub-YFP cells transfected with G3BP1/2 siRNA AND siRNA-resistant G3BP1 mutants YFP intensities. Normalized to mCherry untreated control cells (− HS). The frequency and distribution of the relative fluorescent intensity per cell are shown as violin plots. The solid lines in each distribution represent the median, and dash lines represent the upper and lower interquartile range limits ($n = 3$ independent experiments, > 1,000 cells analyzed per condition, Kruskal-Wallis test, $^{**}P < 0.01$, n.s.—not significant).

In order to investigate whether the aggravated USP Impairment is caused by the inability of G3BP1/2-depleted cells to form stress granules, we performed a rescue experiment with siRNA-resistant wild-type and mutant G3BP1. G3BP1 has been implicated in actions that are independent of its role in stress granule formation but require interaction with the deubiquitinase USP10 (Soncini *et al*, 2001; Kedersha *et al*, 2016; Anisimov *et al*, 2019; Meyer *et al*, 2020). It has been reported that the G3BP1^{F33W} mutant is proficient for stress granule formation but fails to interact with USP10 whereas the G3BP1$^{\Delta RGG}$ is deficient for stress granule formation but still binds USP10 (Panas *et al*, 2015; Kedersha *et al*, 2016). Microscopic analysis confirmed that mCherry-tagged wild-type G3BP1 and G3BP1^{F33W} but not G3BP1$^{\Delta RGG}$ localized in cytosolic puncta in response to heat shock in G3BP1/2 depleted cells (Fig EV1D). In line with our model, we found that wild-type G3BP1 and G3BP1^{F33W} restored the UPS activity in thermally stressed cells to the levels observed in control cells whereas the stress granule-deficient G3BP1$^{\Delta RGG}$ did not rescue UPS activity (Fig 1G).

The impairment of the UPS after thermal stress is confined to the degradation of ubiquitin-dependent proteasome substrates (Salomons *et al*, 2009). We therefore wondered if the aggravated UPS impairment in stress granule-deficient cells could be attributed to an additional defect in ubiquitin-independent proteasomal degradation. However, the ubiquitin-independent proteasome substrate green fluorescent protein (GFP)-ODC did not accumulate in thermally stressed control or stress granule-deficient cells, suggesting that the obstruction only affects substrates that require ubiquitylation for degradation (Fig EV1E and F). We conclude that impairment of ubiquitin-dependent proteasomal degradation is aggravated in stress granule-deficient cells recovering from proteotoxic stress.

Compromised nuclear UPS in stress granule-deficient cells

The UPS is the main proteolytic system responsible for the degradation of short-lived proteins in both the cytosolic and nuclear compartments of cells. Therefore, we next explored if the absence of stress granules had a pan-cellular effect or predominantly affected the UPS in one of these compartments. For this purpose, we took advantage of two compartment-specific CL1 reporter substrates that are engineered to localize to the nuclear (NLS-GFP-CL1) or cytosolic compartment (NES-GFP-CL1) (Bennett *et al*, 2005). Surprisingly, G3BP1/2-depleted cells displayed a significant increase in the accumulation of the nuclear reporter substrate in response to proteotoxic stress (Fig 2A and C), whereas there was no difference between the levels of the cytosolic reporter in control and stress granule-deficient cells (Fig 2B and D). Flow cytometric analysis of the mean fluorescent intensities confirmed that there was a selective increase in the levels of the nuclear reporter in stress granule-deficient cells whereas no significant difference was detected for the cytosolic reporter (Fig EV2A). The relative increase in the levels of the nuclear reporter in G3BP1/2-depleted cells was partly caused by the accumulation of the NLS-GFP-CL1 reporter in nucleoli (Fig 2E). Even though control cells also displayed a slight nucleolar enrichment of NLS-GFP-CL1, quantitative analysis confirmed a more dramatic translocation of this reporter to nucleoli in stress granule-deficient cells (Fig 2F). Together, these data show that the inability of G3BP1/2-depleted cells to induce stress granules after heat shock has a negative impact on the functionality of the UPS in the nuclear compartment and causes sequestration of an aggregation-prone proteasome substrate in nucleoli.

Stress granule-deficient cells accumulate defective ribosome products (DRiPs) in nucleoli

It has been reported that G3BP1/2 is important for preserving proteasome activity in unstressed cells by preventing protein aggregation in the cytosolic compartment (Anisimov *et al*, 2019). However, we did not observe UPS impairment under control conditions in G3BP1/2-depleted cells, suggesting that the phenomenon that we observed is different from a possible effect on protein aggregates (see Fig 1C and D). Moreover, whereas cytosolic protein aggregation causes a global UPS impairment in both the cytoplasmic and nuclear compartments (Bennett *et al*, 2005), we observed that G3BP1/2 depletion in thermal stressed cells aggravated UPS impairment in

 © 2022 The Authors

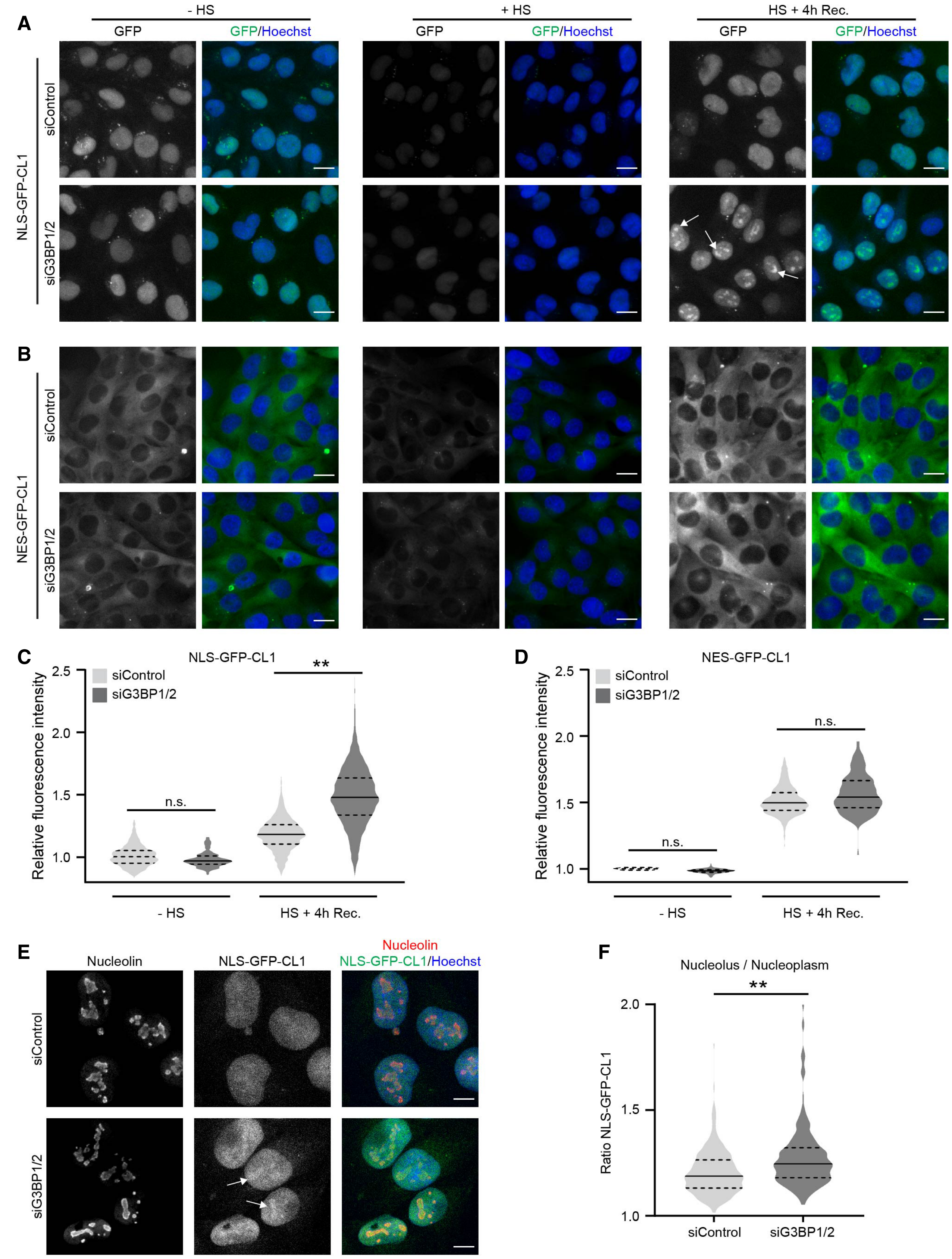
A
- HS
+ HS
HS + 4h Rec.
GFP
GFP/Hoechst
NLS-GFP-CL1
siControl
siG3BP1/2
B
NES-GFP-CL1
C
NLS-GFP-CL1
Relative fluorescence intensity
2.5
2.0
1.5
1.0
n.s.
**
- HS
HS + 4h Rec.
D
NES-GFP-CL1
E
Nucleolin
NLS-GFP-CL1
Nucleolin
NLS-GFP-CL1/Hoechst
F
Nucleolus / Nucleoplasm
Ratio NLS-GFP-CL1

Figure 2.

◀ **Figure 2. Compromised nuclear UPS in stress granule-deficient cells.**

A, B Representative images of stress granule proficient (siControl) and deficient (siG3BP1/2) MelJuSo cells expressing the nuclear UPS reporter NLS-GFP-CL1 (A) and the cytoplasmic UPS reporter NES-GFP-CL1 (B). The cells were left untreated (− HS), exposed to 43°C for 30 min (HS), or exposed to 43°C for 30 min and followed by 4 h recovery (HS + 4 h Rec.). Nucleolar accumulation of NLS-GFP-CL1 during the recovery is indicated by arrows in (A). Scale bar, 20 μm.

C, D Quantifications of the mean cellular GFP fluorescence intensities of (A) and (B) normalized to untreated control cells (siControl - HS). The frequency and distribution of the relative fluorescence intensities per cell are shown as violin plots. The solid lines in each distribution represent the median, and dash lines represent the upper and lower interquartile range limits (n = 3 independent experiments, > 1,000 cells analyzed per condition, Kruskal-Wallis test, $^{**}P < 0.01$, n.s.—not significant).

E Representative confocal images of immunofluorescent staining of the nucleolar marker nucleolin and the nuclear UPS reporter NLS-GFP-CL1 in control (siControl, left panel) and G3BP1/2-depleted (siGRBP1/2, right panel) MelJuSo cells exposed to a 30 min 43°C heat shock, followed by 4 h at 37°C (Recovery). Nucleolar accumulation of NLS-GFP-CL1 is indicated by arrows. Scale bar, 10 μm.

F Quantification of the nucleolus/nucleoplasm ratios of NLS-GFP-CL1 intensities in images of the experiment shown in (E). The frequency and distribution of the ratio per cell are shown as violin plots. The solid lines in each distribution represent the median, and dash lines represent the upper and lower interquartile range limits (n = 3 independent experiments, 100 cells analyzed per condition, Kruskal-Wallis test, $^{**}P < 0.01$).

the nucleus without detectable effects on the UPS in the cytosol. Hence, we decided to further explore the molecular mechanisms underlying the aggravated UPS impairment in stressed G3BP1/2-deficient cells. For this purpose, we used a U2OS cell line in which the genes encoding G3BP1 and G3BP2 have been deleted (Kedersha *et al*, 2016). First, we introduced the Ub-YFP reporter substrate in parental and G3BP1/2 knockout U2OS cells to analyze whether a similar effect on UPS functionality was observed as in MelJuSo cells subjected to siRNA-mediated downregulation of G3BP1/2. Microscopic and flow cytometric analysis indeed confirmed that the lack of G3BP1/2 aggravated stress-induced UPS impairment in U2OS cells without causing accumulation of the reporter under control conditions (Fig EV2B and C).

To probe into the mechanism responsible for aggravated UPS impairment, we investigated how the lack of G3BP1/2 affects the behavior of misfolded proteins in cells recovering from proteotoxic stress. To this end, we labeled newly synthesized proteins during the heat shock with puromycin. As puromycin causes premature termination of protein translation, the puromycin-labeled proteins are a source of defective ribosome products (DRiPs) that are well-established substrates for the UPS (Schubert *et al*, 2000) and that at the same time can be readily detected with puromycin-specific antibodies (Schmidt *et al*, 2009). In parental U2OS cells, thermal stress resulted in the localization of DRiPs in cytosolic TIA1-positive puncta (Fig 3A), consistent with the model that misfolded proteins are sequestered at stress granules (Mateju *et al*, 2017). The lack of stress granules in G3BP1/2 knockout cells was accompanied by translocation of the DRiPs to the nucleus (Fig 3B), where they were enriched in nucleoli (Fig 3C and D), similar to our observations with the nuclear proteasome substrate NLS-GFP-CL1 (Fig 2E and F). The nucleolar sequestration of DRiPs was more persistent as elevated levels could still be detected 2 h after heat shock whereas the stress granules and associated DRiPs were dispersed within 1 h after heat shock (Fig EV2D and E). The difference between parental and G3BP1/2 knockout cells was striking as the enrichment of the DRiPs in nucleoli of the stress granule-deficient cells sharply contrasted with the nucleoli in parental cells that were relatively devoid of DRiPs. The integrity of nucleoli was critical for the sequestration of DRiPs in stress granule-deficient cells as treatment of the cells with actinomycin distorted the nucleolar structure and resulted in a diffuse distribution of the DRiPs through the nucleoplasm (Fig EV3A).

To investigate whether the DRiPs are actively transported to the nuclear compartment in stressed granule-deficient cells, we analyzed the effect of administration of the importin α/β inhibitor ivermectin on the distribution of the DRiPs in stressed cells. While ivermectin prevented nuclear localization of the nuclear protein ataxin-1 (Fig EV3B), it did not inhibit the accumulation of DRiPs in nucleoli of stressed G3BP1/2 knockout cells (Fig EV3C), suggesting that DRiPs passively diffuse into the nuclear compartment (Mediani *et al*, 2019b). The altered localization of the DRiPs did not affect their ubiquitylation status as similar amounts of ubiquitylated DRiPs were pulled down with tandem ubiquitin-binding entities (TUBEs) from parental and G3BP1/2 knockout cells (Fig EV3D).

The molecular chaperone Hsp70, also known as HSPA1A, plays an important role in the subcellular localization of DRiPs (Andersson *et al*, 2021). Accordingly, we found that localization of DRiPs in stress granules and nucleoli of parental and G3BP1/2 knockout cells, respectively, was also dependent on Hsp70 as siRNA-mediated depletion of this chaperone abrogated the translocation of DRiPs at stress granules (Figs 3E and F and EV3E) and nucleoli (Figs 3G and H and EV3E). Together these data show that DRiPs reach the nuclear compartment of stress granule-deficient cells by passive diffusion through the nuclear pore where they accumulate in nucleoli in an Hsp70-dependent fashion.

Altered heat shock response in stress granule-deficient cells

Besides a difference in localization, the presence of DRiPs in nucleoli was also more persistent as this could still be detected in G3BP1/2 knockout cells 1 h after exposure to the thermal stress whereas the stress granules and stress granule-localized DRiPs in parental cells had been dissolved at that time point (Fig 4A). Consistent with a role of Hsp70 in the redistribution of the misfolded proteins, we observed that Hsp70 also followed the distribution of DRiPs in stressed parental and G3BP1/2 knockout cells, where enrichment of Hsp70 could be observed at stress granules and in nucleoli, respectively (Fig 4A). Like the DRiPs, Hsp70 could also still be detected enriched in nucleoli 1 h after the heat shock in G3BP1/2 knockout cells, a time point at which the stress granules and DRiPs localized at stress granules had been resolved (Fig 4A). This suggests not only a translocation but also a more persistent sequestration of DRiPs and Hsp70 in stress granule-deficient cells.

The levels of misfolded proteins are an important determinant for activation of the heat shock response as they titrate Hsp70 away from heat shock factor 1 (HSF1), which once released from Hsp70

 © 2022 The Authors

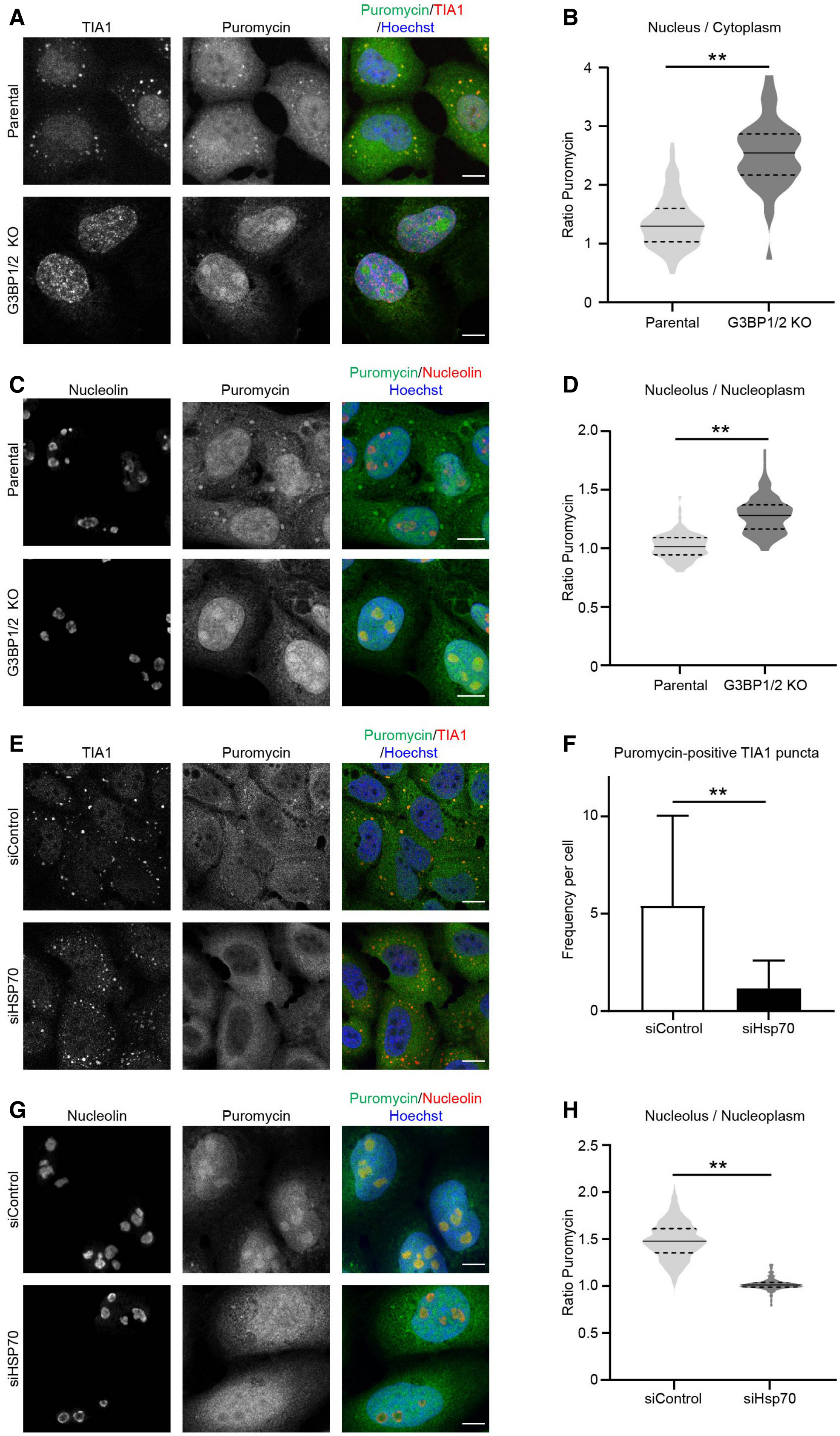
A
TIA1
Puromycin
Puromycin/TIA1/Hoechst
Parental
G3BP1/2 KO
B
Nucleus / Cytoplasm
Ratio Puromycin
**
Parental
G3BP1/2 KO
C
Nucleolin
Puromycin
Puromycin/Nucleolin
Hoechst
Parental
G3BP1/2 KO
D
Nucleolus / Nucleoplasm
Ratio Puromycin
**
Parental
G3BP1/2 KO
E
TIA1
Puromycin
Puromycin/TIA1/Hoechst
siControl
siHSP70
F
Puromycin-positive TIA1 puncta
Frequency per cell
**
siControl
siHsp70
G
Nucleolin
Puromycin
Puromycin/Nucleolin
Hoechst
siControl
siHSP70
H
Nucleolus / Nucleoplasm
Ratio Puromycin
**
siControl
siHsp70

Figure 3.

◀ **Figure 3. Stress granule-deficient cells accumulate defective ribosome products (DRiPs) in nucleoli.**

A Representative confocal images of immunofluorescent staining of the stress granule marker TIA1 and puromycin-labeled proteins in parental and G3BP1/2 knockout U2OS cells. Cells were subjected to 43°C heat shock. Scale bar, 10 μm.

B Quantification of the nucleus/cytoplasm ratios of puromycin intensities in images from (A). The frequency and distribution of the ratio per cell are shown as violin plots. The solid lines in each distribution represent the median, and dash lines represent the upper and lower interquartile range limits ($n = 3$ independent experiments, > 50 cells analyzed per condition, Kruskal-Wallis test, $^{**}P < 0.01$).

C Representative confocal images of immunofluorescent staining of the nucleolar marker nucleolin and puromycin-labeled proteins in parental and G3BP1/2 knockout U2OS cells. Cells were subjected to 43°C heat shock. Scale bar, 10 μm.

D Quantification of the nucleolus/nucleoplasm ratio of puromycin intensities in images from (B). The frequency and distribution of the ratio per cell are shown as violin plots. The solid lines in each distribution represent the median, and dash lines represent the upper and lower interquartile range limits ($n = 3$ independent experiments, > 50 cells analyzed per condition, Kruskal-Wallis test, $^{**}P < 0.01$).

E Representative confocal images of immunofluorescent staining of the stress granule marker TIA1 and puromycin-labeled proteins in control (siControl) and Hsp70-depleted (siHsp70) U2OS cells. Cells were subjected to 43°C heat shock Scale bar, 10 μm.

F Quantification of the numbers of puromycin-positive TIA dots per cell in images from (E). The numbers of positive foci per cell are shown as violin plots. The solid lines in each distribution represent the median, and dash lines represent the upper and lower interquartile range limits ($n = 3$ independent experiments, > 50 cells analyzed per condition, Kruskal-Wallis test, $^{**}P < 0.01$).

G Representative confocal images of immunofluorescent staining of the nucleolar marker nucleolin and puromycin-labeled proteins in control (siControl) and Hsp70-depleted (siHsp70) G3BP1/2 knockout U2OS cells. Cells were subjected to 43°C heat shock. Scale bar, 10 μm.

H Quantification of the nucleolus/nucleoplasm ratio of puromycin intensities in images from (G). The frequency and distribution of the ratio per cell are shown as violin plots. The solid lines in each distribution represent the median, and dash lines represent the upper and lower interquartile range limits ($n = 3$ independent experiments, > 50 cells analyzed per condition, Mann–Whitney test, $^{**}P < 0.01$).

activates a transcriptional program aimed at restoring proteostasis (Masser *et al*, 2020). Hence, we investigated whether the altered behavior of Hsp70 in stress granule-deficient cells was accompanied by changes in activation of the heat shock response. Two parameters for activation of the heat shock response are the formation of HSF1-positive nuclear stress bodies (nSBs) (Zhang *et al*, 2022) and phosphorylation of HSF1 (Holmberg *et al*, 2001), which both positively correlate with activation of the heat shock response.

Microscopic analysis revealed that G3BP1/2 knockout cells have more HSF1 nSBs directly after heat shock than parental cells (Fig 4B and C). The nSBs had dissolved to a greater extent 2 h after the heat shock in G3BP1/2 knockout cells, suggesting that the activation of the heat shock response has shifted to an earlier time point in stress granule-deficient cells. Phosphorylation of HSF1 followed a similar pattern with an increase in phosphorylation of HSF1 directly after heat shock in G3BP1/2 knockout cells, which had returned to basal levels 2 h post-heat shock, whereas the parental cells still displayed elevated levels of phosphorylated HSF1 at that time point (Fig 4D). Quantitative analysis of Hsp70 transcripts, which is a transcriptional target of HSF1 (Jolly *et al*, 1997), also confirmed a premature activation of the heat shock response in G3BP1/2 knockout cells (Fig 4E). Together, these data suggest that in stress granule-deficient cells misfolded proteins are sequestered together with Hsp70 in nucleoli, which is accompanied by early activation of HSF1 during the recovery phase.

Nuclear stress-induced SUMOylation is changed in stress granule-deficient cells

Targeting of SUMOylated proteins for ubiquitin-dependent proteasomal degradation plays an important role in the maintenance of nuclear proteostasis during proteotoxic stress (Guo *et al*, 2014). A key player in this stress response is the SUMO-targeted ubiquitin ligase (STUbL) RNF4, which binds SUMOylated proteins and modifies them with ubiquitin chains that direct them for proteasomal degradation (Tatham *et al*, 2008). As stress-induced HSF1 stimulates the ubiquitin-dependent degradation of SUMOylated proteins by RNF4 (Hendriks *et al*, 2015), we wondered if the altered activation of the heat shock response in stress granule-deficient cells was responsible for the aggravated nuclear UPS impairment in these cells.

To probe into this issue, we first had a closer look at the behavior of SUMO conjugates in parental and G3BP1/2 knockout cells. We noted that there was a striking difference in the localization of SUMO2/3 in these cells with a clear enrichment of SUMO2/3 in nucleoli of parental cells, whereas it was primarily localized in the nucleoplasm and excluded from nucleoli in G3BP1/2 knockout cells (Fig 5A and B). This was accompanied in stress granule-deficient cells with an increase in SUMO2/3-positive puncta that contained promyelocytic leukemia (PML) protein (Fig 5C and D), a classic stress-induced substrate for SUMO-targeted ubiquitylation (Tatham *et al*, 2008). As PML bodies are sites where SUMOylated proteins are targeted for ubiquitin-dependent proteasomal degradation, the increased co-localization could be indicative of enhanced activation of this pathway (Mediani *et al*, 2019b). In line with this model, the levels of SUMOylated proteins rapidly declined during the early recovery phase in thermally stressed G3BP1/2-deficient cells, which could be prevented by administration of the proteasome inhibitor epoxomicin (Fig 5E and F). Such a decline in SUMO conjugates was not observed in stressed parental cells. There was no significant difference in SUMO2 deconjugase activity in lysates of stress granule-proficient and -deficient cells recovering from thermal stress (Fig EV3F), consistent with the model that the difference in the levels of SUMO conjugates was due to enhanced degradation of SUMOylated proteins and not accelerated disassembly of SUMO chains. As the proteolytic activity (Fig EV3G) and localization of proteasomes (Fig EV3H) did not differ between the two cell lines, the enhanced degradation is most likely caused by more efficient targeting of SUMOylated proteins for proteasomal degradation rather than boosting proteasome activity. Together this suggests that clearance of SUMOylated proteins during the early recovery phase is accelerated in stress granule-deficient cells by enhanced targeting of these substrates for ubiquitin-dependent proteasomal degradation.

 © 2022 The Authors

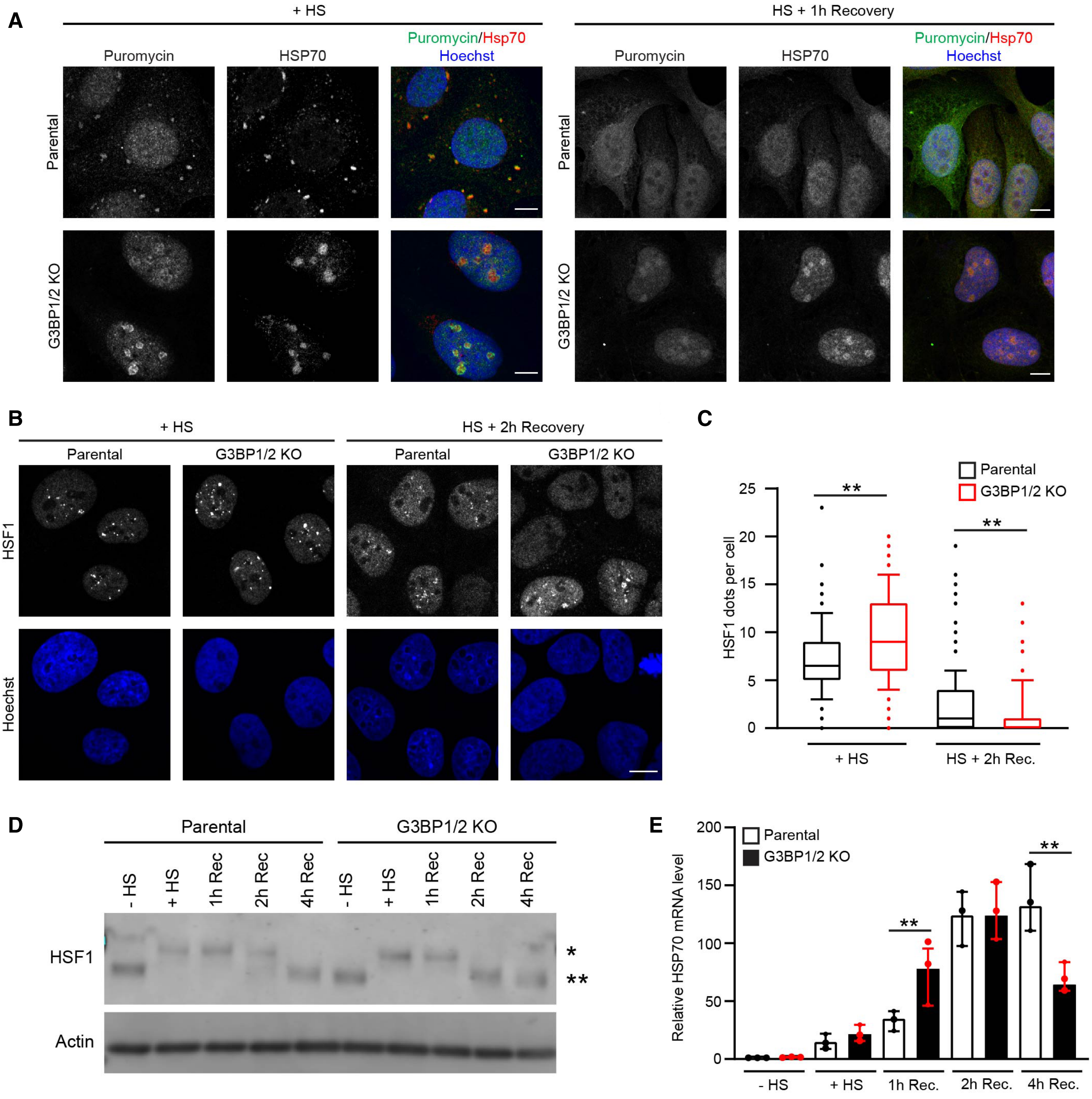

Figure 4. Altered heat shock response in stress granule-deficient cells.

A Representative confocal images of immunofluorescent staining of and puromycin-labeled proteins and Hsp70 in parental and G3BP1/2 knockout U2OS cells exposed to a heat shock at 43°C for 30 min (+ HS; left panel), and after 1 h recovery (HS + 1 h Recovery; right panel). Scale bar, 10 μm.

B Representative confocal images of immunofluorescent staining of HSF1 in parental and G3BP1/2 knockout U2OS cells directly after heat shock (+ HS) and 2 h after 2 h recovery (HS + 2 h Recovery). Scale bar, 10 μm.

C Quantification of the number of HSF1 foci per cell in images from (B), shown as a box plot with median and 5–95 percentiles (n = 3 independent experiments, > 300 cells analyzed per condition, Kruskal-Wallis test, $^{**}P < 0.01$).

D Western blot analysis of parental and G3BP1/2 knockout U2OS cells that were left untreated (— HS), heat-shocked (HS), and followed for 1, 2, and 4 h after heat shock (Rec). The blots were probed with antibodies against HSF1 and GAPDH (* indicated phosphorylated HSF1, and ** indicates unphosphorylated HSF1).

E Analysis of *HSP70* mRNA levels in parental and G3BP1/2 knockout U2OS cells that were left untreated (— HS), heat-shocked (+ HS), and followed for 1, 2, and 4 h after heat shock (Rec.). mRNA levels were normalized to untreated samples. Data represent the geometric mean with 95% confidence interval of triplicate samples (Student's unpaired t-test, $^{**}P < 0.01$).

© 2022 The Authors

Enhanced degradation of STUbL substrate TDP-43 in stress granule-deficient cells

We decided to have a closer look at TDP-43 in stress granule-deficient cells as this RNA-binding protein has been shown to be targeted by the STUbL RNF4 for proteasomal degradation (Guo *et al*, 2014) and is also involved in the formation of stress granules (McGurk *et al*, 2018). Moreover, TDP-43 is linked to ALS and found as a major component in the inclusion bodies of various neurodegenerative diseases (Sreedharan *et al*, 2008). Immunostaining showed that there was a dramatic increase in nuclear puncta that contained TDP-43 and SUMO2/3 in stress granule-deficient cells (Fig 6A and B). Similar nuclear puncta were observed with GFP-tagged TDP-43, which were found to co-localize with both SUMO2/

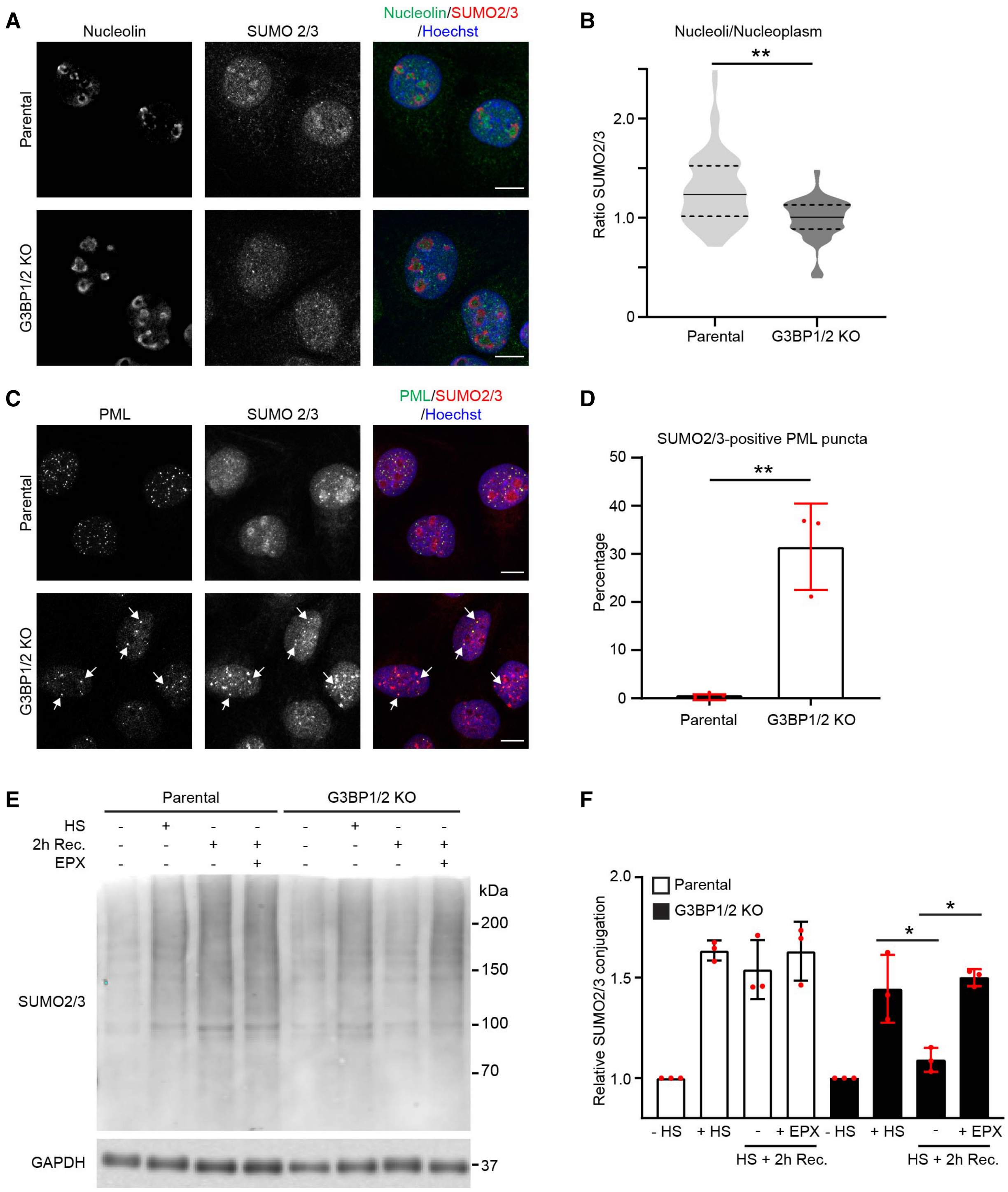

Figure 5.

© 2022 The Authors

Figure 5. Nuclear stress-induced SUMOylation is changed in stress granule-deficient cells.

A Representative confocal images of immunofluorescent staining of the nucleolar marker nucleolin and SUMO2/3 in parental and G3BP1/2 knockout U2OS cells. Cells were subjected to a heat shock (30 min, 43°C) followed by 2 h recovery. Scale bar, 10 µm.

B Quantification of the nucleolus/nucleoplasm ratio of SUMO2/3 intensities in images from (A). The frequency and distribution of the ratio per cell are shown as violin plots. The solid lines in each distribution represent the median, and dash lines represent the upper and lower interquartile range limits ($n = 3$ independent experiments, > 50 cells analyzed per condition, Mann–Whitney test, $^{**}P < 0.01$).

C Representative confocal images of immunofluorescent staining of PML bodies (PML) and SUMO2/3 in parental and G3BP1/2 knockout U2OS cells. Cells were heat-shocked followed by 2 h recovery. Arrows indicate dots positive for PML and SUMO2/3 staining. Scale bar, 10 µm.

D Quantification of SUMO2/3 positive PML puncta in images from (C). Data represent the mean ± SD ($n = 3$ independent experiments, > 50 cells analyzed per condition, Mann–Whitney test, $^{**}P < 0.01$).

E Analysis of SUMO2/3 conjugates in parental and G3BP1/2 knockout U2OS cells that were left untreated (− HS), exposed to a heat shock (+ HS), and followed for 2 h (2 h Rec.) with or without 100 nM proteasome inhibitor epoxomicin (EPX).

F Quantification of the total SUMO2/3 conjugate band densities in (E). Data represent the mean ± SD ($n = 3$ independent experiments, Student's unpaired t-test, $^{*}P < 0.05$).

3 and PML (Fig 6C), supporting the model that these are sites for targeting TDP-43 for SUMO/ubiquitin-dependent degradation. Enhanced degradation of TDP-43 was confirmed in fractionation experiments in which we observed that the levels of SDS-soluble/NP40-insoluble TDP-43 were reduced during the recovery phase in stress granule-deficient but not stress granule-proficient cells (Fig 6D and E). The reduction in SDS-soluble TDP-43 levels in G3BP1/2 knockout cells could be prevented by administration of proteasome inhibitor, confirming that proteasomal degradation is responsible for the enhanced clearance of this population of TDP-43 (Fig 6D and E). These data suggest that there is enhanced targeting of the STUbL substrate TDP-43 for proteasomal degradation during the recovery phase of G3BP1/2 knockout cells.

Inhibition of SUMO-targeted ubiquitylation prevents UPS impairment

We argued that the enhanced targeting of misfolded proteins for ubiquitin/SUMO-dependent degradation during the early recovery phase in G3BP1/2-deficient cells could be responsible for the observed impairment of the nuclear UPS as these substrates may overload the capacity of the UPS in this compartment. To address this question, we investigated whether inhibition of the SUMO/ubiquitin pathway could restore the UPS in the nucleus. Depletion of RNF4 prevented to a large extent the reduction in SUMOylated proteins G3BP1/2-deficient cells recovering from thermal stress (Figs 7A and B and EV4A), although not to the same extent as proteasome inhibition, indicating that RNF4 is responsible for targeting the vast majority of SUMOylated proteins for degradation. Interestingly, depletion of RNF4 had no effect on the accumulation of Ub-YFP in stressed parental cells (Fig 7C and EV4B and C), whereas it significantly reduced the accumulation of the Ub-YFP reporter substrate in G3BP1/2 knockout cells, in agreement with RNF4 substrates being primarily responsible for the aggravated impairment of the UPS in stress granule-deficient cells (Figs 7D and EV4B and D). Moreover, the nucleolar accumulation of the NLS-GFP-CL1 reporter was also prevented by RNF4 depletion in stress granule-deficient cells (Fig 7E). PML bodies play a pivotal role in SUMOylation and ubiquitylation of nuclear proteins (Keiten-Schmitz *et al*, 2020). Interestingly, depletion of PML, like RNF4 depletion, rescued the UPS impairment in G3BP1/2 knockout cells (Fig EV4E and F). This underscores the role of SUMO-targeted ubiquitylation in the UPS dysfunction observed in stress granule-deficient cells. These data are in line with our hypothesis that the enhanced targeting of misfolded proteins for proteasomal degradation in the nuclear compartment overwhelms the nuclear UPS and aggravates UPS impairment.

Enhanced formation of ataxin-1 nuclear inclusions in stress granule-deficient cells

Given the important role of the UPS in nuclear proteostasis, its dysfunctional status in stress granule-deficient cells is likely to have consequences in keeping this compartment devoid of aggregation-prone proteins. We studied possible effects on nuclear proteostasis by transiently expressing a mutant ataxin-1, which carries an expanded polyglutamine repeat and is responsible for the inheritable neurodegenerative disorder spinocerebellar ataxia type 1 (SCA-1), characterized by the presence of intranuclear inclusions with as main constituent mutant ataxin-1 (Zoghbi & Orr, 2000). We took advantage of an artificial precursor protein that allows the identification of ataxin-1-expressing cells independent on the expression levels of the mutant protein (Verhoef *et al*, 2002). The ^{FLAG}Ub-ataxin-1 is cleaved by endogenous deubiquitylating proteins in FLAG-tagged ubiquitin (^{FLAG}Ub) and mutant ataxin-1 allowing identification of transfected cells by the signal of the stable, soluble ^{FLAG}Ub, which is synthesized at stochiometric levels as ataxin-1. Western blotting showed that similar expression levels were obtained in both cell lines (Fig EV4G and H). Interestingly, thermally stressed G3BP1/2 knockout cells contained more and larger ataxin-1 inclusions than the parental cells (Fig 8A). Quantitative assessment confirmed an overall increase in ataxin-1 inclusions in G3BP1/2 knockout cells (Fig 8B). This supports the model that the aggravated impairment of the nuclear UPS compromises the ability of stress granule-deficient cells to eliminate aggregation-prone proteins in the nuclear compartment.

Discussion

During recent years, the importance of stress granule formation as part of the cellular response to proteotoxic stress conditions, and their implication in various disorders has gained considerable interest in the scientific community (Riggs *et al*, 2020). Not the least the central role of liquid phase separation in the formation of these molecular condensates has put the spotlight on stress granules as a prime example of dynamic, membrane-less subcellular structures consisting of self-assembled biomacromolecules (Boeynaems *et al*, 2018). Since liquid phase transition is driven by proteins with

© 2022 The Authors

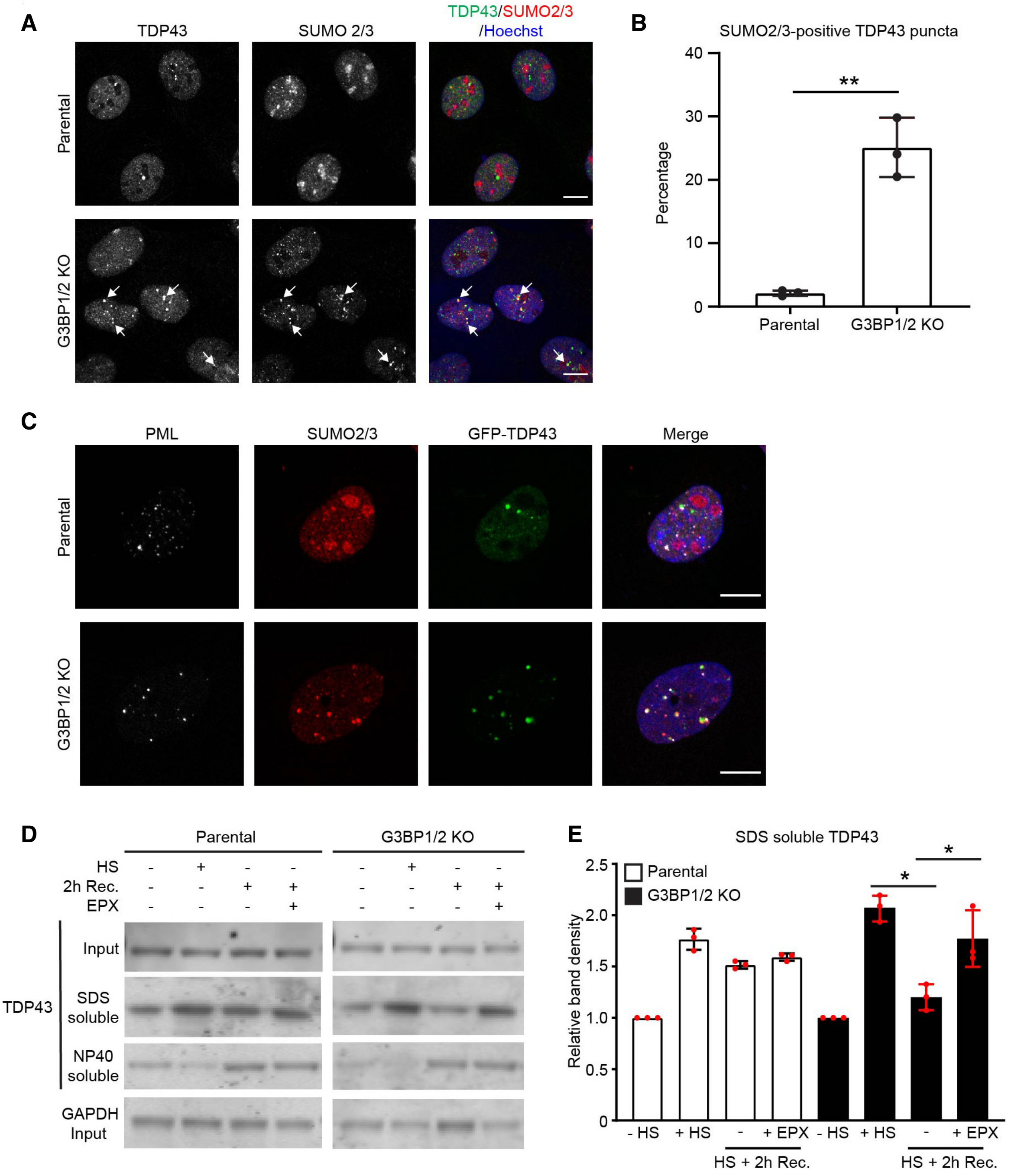

Figure 6. Enhanced degradation of STUbL substrate TDP-43 in stress granule-deficient cells.

A Representative confocal images of immunofluorescent staining of SUMO2/3 and TDP43 in parental and G3BP1/2 knockout U2OS cells. Cells were exposed to a heat shock (30 min, 43°C) followed by 2 h recovery. Arrows indicate dots positive for TDP-43 and SUMO2/3 staining. Scale bar, 10 μm.

B Quantification of the percentage of SUMO2/3 positive TDP43 puncta in images from the experiment shown in (A). Data represent the mean ± SD (n = 3 independent experiments, > 50 cells analyzed per condition, Mann–Whitney test, ** $P < 0.01$).

C Representative confocal images of immunofluorescent staining of SUMO2/3 and PML bodies (PML) in parental and G3BP1/2 knockout U2OS cells transiently transfected with GFP-TDP43. Cells were subjected to heat shock and followed by 2 h recovery. Scale bar, 10 μm.

D Analysis of detergent SDS and NP40 soluble fractions of TDP43 in parental and G3BP1/2 knockout U2OS cells that were left untreated (−HS), exposed to a heat shock (+HS) followed by 2 h recovery (2 h Rec.) in absence or presence of 100 nM proteasome inhibitor epoxomicin (EPX).

E Quantification of the TDP43 band densities in the SDS-soluble fraction. Data represent the mean ± SD (n = 3 independent experiments, Student's unpaired t-test, * $P < 0.05$).

© 2022 The Authors

intrinsically disordered domains, it is not surprising that misfolded proteins have a propensity to coalescence in subcellular structures that rely on liquid phase separation for their macromolecular organization (Mateju *et al*, 2017). While the importance of stress granules as a temporary storage for untranslated mRNA transcripts is well-established, it is less clear if the localization of misfolded proteins in these structures has functional significance for the cell's ability to deal with stress conditions. Since the translocation of

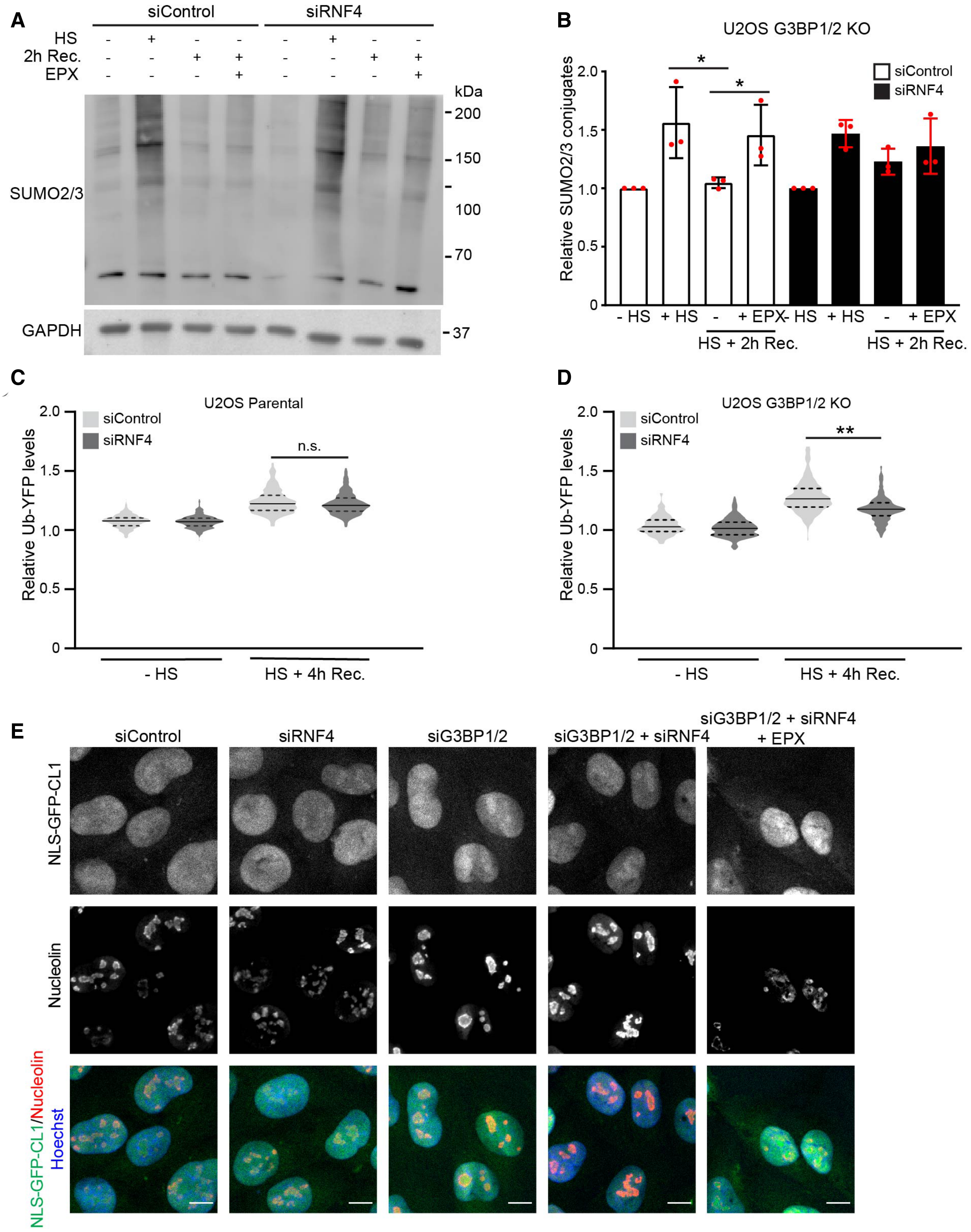

Figure 7.

Figure 7. Inhibition of SUMO-targeted ubiquitylation prevents UPS impairment.

A Analysis of SUMO2/3 conjugates in control (siControl) or RNF4-depleted (siRNF4) G3BP1/2 knockout U2OS cells. Cells were left untreated (−HS), exposed to a heat shock (+HS), followed by 2 h recovery (HS + 2 h Rec.) in the absence or presence of 100 nM proteasome inhibitor epoxomicin (EPX).

B Quantification of the total SUMO2/3 band densities. Data represent the mean ± SD ($n = 3$ independent experiments, $^*P < 0.05$).

C Quantification of mean cellular Ub-YFP fluorescence intensities in control (siControl) and RNF4-depleted (siRNF4) parental U2OS cells. Cells were left untreated (−HS) or exposed to a heat shock followed by 4 h recovery (HS + 4 h Rec.). Fluorescence intensities are normalized to untreated control cells (siControl - HS). Frequency and distribution of the relative fluorescence intensities are shown as violin plots. The solid lines in each distribution represent the median, and dash lines represent the upper and lower interquartile range limits ($n = 3$ independent experiments, > 1,000 cells analyzed per condition, Kruskal-Wallis test, n.s.—not significant).

D Quantification of mean cellular Ub-YFP fluorescence intensities in control (siControl) and RNF4-depleted (siRNF4) G3BP1/2 knockout U2OS cells. Cells were left untreated (−HS) or exposed to a heat shock followed by 4 h recovery (HS + 4 h Rec.). Fluorescence intensities are normalized to untreated control cells (siControl - HS). Frequency and distribution of the relative fluorescence intensities are shown as violin plots. The solid lines in each distribution represent the median, and dash lines represent the upper and lower interquartile range limits ($n = 3$ independent experiments, > 1,000 cells analyzed per condition, Kruskal-Wallis test, $^{**}P < 0.01$).

E Representative confocal images of control (siControl), RNF4-depleted (siRNF4), G3BP1/2-depleted (siG3BP1/2) and G3BP1/2- and RNF4-depleted (siG3BP1/2 + siRNF4) MelJuSo cells expressing the nuclear UPS reporter NLS-GFP-CL1. Cells were exposed to a heat shock followed by 4 h recovery in the absence or presence of the proteasome inhibitor epoxomicin (EPX). The nucleolar marker nucleolin was stained by immunofluorescence. Scale bar, 10 μm.

misfolded proteins to inclusion bodies has been shown to reduce the cellular toxicity caused by aggregation-prone proteins (Arrasate *et al*, 2004), it is feasible that also this specific feature of stress granules contributes to their stress-coping abilities.

In this study, we show that the accumulation of DRiPs in stress granules is important for keeping the UPS operative when cells recover from acute exposure to proteotoxic stress. As it has been well documented that a failure to maintain proteostasis causes cellular dysfunction (Sala *et al*, 2017), the compromised ability of stress granule-deficient cells to execute ubiquitin-dependent proteasomal degradation is expected to have negative consequences for maintaining proteostasis. Given the connection between stalled protein synthesis and stress granules, it is plausible that the proximity of stress granules to stalled ribosomes is of particular importance as it may facilitate the instant capturing of newly synthesized proteins that have not been able to fold properly during proteotoxic stress. Moreover, newly synthesized proteins constitute a major pool of aberrant proteins even in the absence of extrinsic stress conditions (Reits *et al*, 2000; Schubert *et al*, 2000), emphasizing the importance of efficiently capturing this population of aberrant proteins.

Surprisingly, our study revealed that it is primarily the nuclear UPS that is challenged in the stress granule-deficient cells. Because of the cytosolic localization of stress granules, we initially expected the pool of misfolded proteins that are not captured by stress granules to contaminate first and foremost the cytosolic compartment. Unexpectedly, our analysis revealed that, in the absence of stress granules, DRiPs rapidly translocate to the nuclear compartment, where they accumulate primarily in nucleoli. A likely explanation for this phenomenon may be the fact that, next to cytoplasmic stress granules, nucleoli are liquid phase-separated structures that can also function as a sink for the sequestration of misfolded proteins (Frottin *et al*, 2019). Hence, in the absence of stress granules, the DRiPs may condensate at these locations in the nucleus because their limited size will allow passive diffusion to the nuclear compartment where they will be readily trapped in nucleoli due to the tendency of these disordered polypeptides to participate in liquid phase separation. The sheer volume of nucleoli will increase the likelihood that DRiPs will end up in these structures. It is noteworthy though that we observed that localization of DRiPs at stress granules and nucleoli requires the presence of Hsp70, as has been reported by others (Nollen *et al*, 2001; Askanas *et al*, 2009; Kotoglou *et al*, 2009; Frottin *et al*, 2019). The interaction with Hsp70 may prevent aggregation of the DRiPs or otherwise keep them in a state that is susceptible to phase separation. The nuclear accumulation of DRiPs may be unfortunate from the perspective of the integrated stress response as this will transfer the burden of these potentially toxic proteins to the nuclear compartment, which is less well equipped to eliminate them due to, among others, the absence of the autophagic-lysosomal system. Since the degradation of aggregated proteins is problematic for the UPS (Verhoef *et al*, 2002), a situation may arise where any delay in the removal of these aberrant proteins may cause an irreversible disturbance of the nuclear proteostasis. Thus, our data suggest that the temporary sequestration of misfolded proteins in stress granules is important for the maintenance of nuclear proteostasis.

The nuclear accumulation of DRiPs directly affected the distribution of Hsp70, which largely followed the cellular distribution of DRiPs. Buffering of the pool of nuclear Hsp70 by misfolded proteins, thereby releasing HSF1 (Masser *et al*, 2020), is a likely cause for the observed premature activation of HSF1 that was evidenced by an increase in HSF1 nSBs, phosphorylation of HSF1 and transcription of the HSP70 gene during the early recovery phase. It has been shown that HSF1 stimulates ubiquitin-dependent proteasomal degradation of SUMOylated proteins through the STUbL RNF4 (Hendriks *et al*, 2015). In line with a modified SUMO landscape in nuclei of stress granule-deficient cells, we observed an altered distribution and increased co-localization of SUMO, ubiquitin, and PML, which coincided with enhanced degradation of RNF4 substrates. Importantly, curtailing this pathway through depletion of the STUbL RNF4 improved the functionality of the nuclear UPS in stress granule-deficient but not stress granule-proficient cells. This supports our hypothesis that the redistribution of DRiPs to the nucleolar compartment buffers the pool of nuclear Hsp70, resulting in early activation of the SUMO-ubiquitin-RNF4 protein quality pathway, which on its turn overloads the nuclear UPS with substrates during the recovery phase (Fig 8C). Our observation that degradation of ubiquitin-independent substrates was not inhibited suggests that a ubiquitin-dependent event prior the actual proteolysis in the proteasome is saturated in stress granule-deficient cells. Thus, the inability of cells to temporarily sequester DRiPs in cytosolic stress granules has persistent consequences for nuclear ubiquitin-dependent proteasomal degradation, compromising protein quality control in this compartment.

The disbalance in nuclear proteostasis in stress granule-deficient cells is likely to bear relevance to neurodegenerative diseases as it may cause a transient state of UPS dysfunction during which aggregation-prone proteins can accumulate and precipitate into insoluble protein aggregates. Indeed, we found that the propensity of mutant ataxin-1 to form ubiquitin-positive nuclear inclusions, which are a hallmark of SCA-1 cellular pathology (Skinner

 © 2022 The Authors

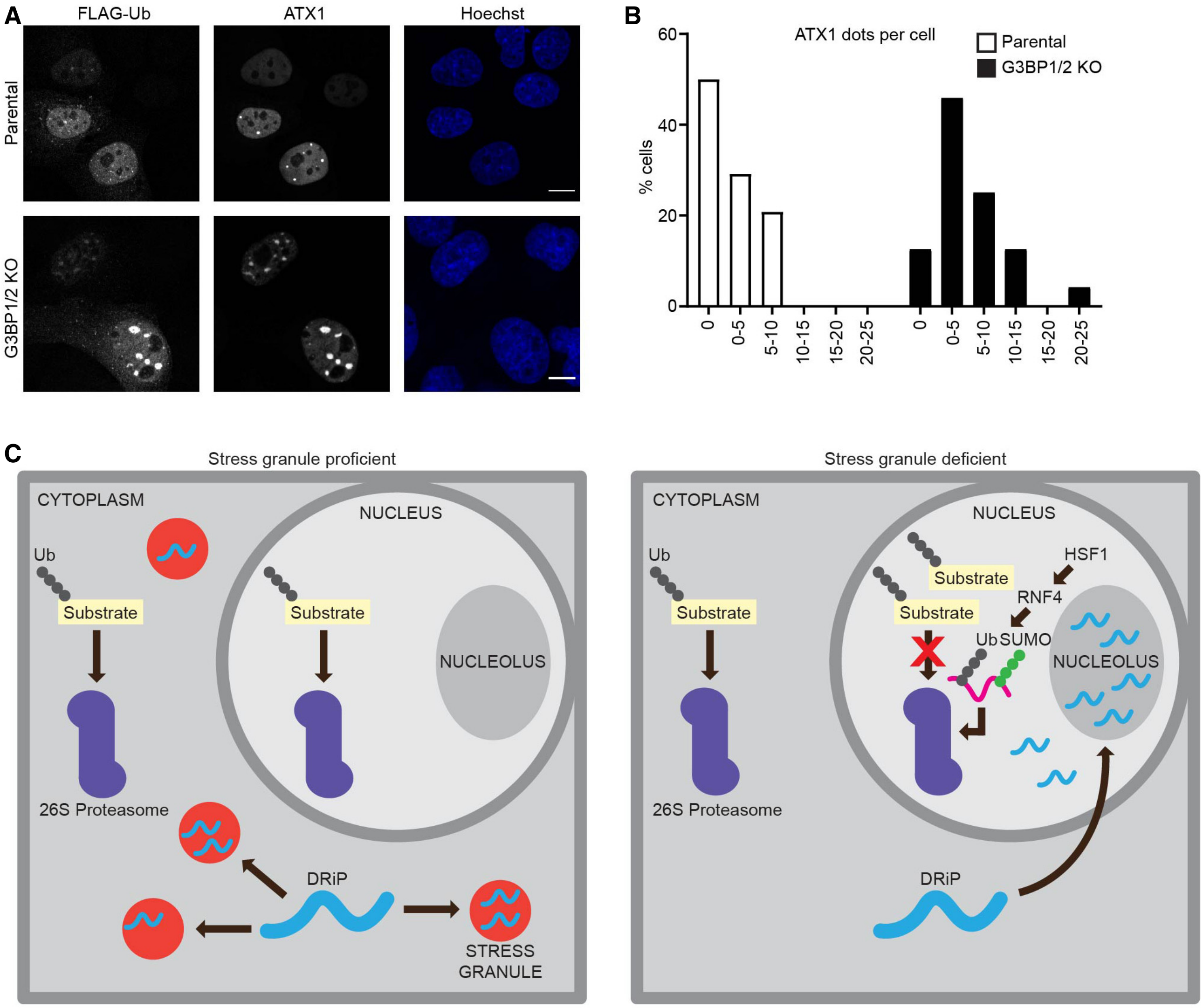

Figure 8. Enhanced formation of ataxin-1 nuclear inclusions in stress granule-deficient cells.

A Representative confocal images of immunofluorescent staining of FLAG-tagged ubiquitin (^{FLAG}Ub) and mutant ataxin1 (ATX1) in parental and G3BP1/2 knockout U2OS cells. Cells were subjected to a heat shock followed by 4 h recovery. Scale bar, 10 µm.

B Frequency distribution of ataxin-1 inclusions per cell from three independent experiments.

C Schematic drawing of the model. In the absence of stress granules, proteotoxic stress causes DRiPs to translocate from the cytosol to the nucleus where they accumulate in nucleoli and sequester Hsp70. This results in early activation of HSF1 and RNF4 and targeting of SUMOylated proteins for proteasomal degradation. These substrates overload the nuclear UPS impairing the degradation of other ubiquitin-dependent proteasome substrates.

et al, 1997), is increased in cells that are unable to respond to stress by the formation of stress granules. This may provide an explanation why several proteins involved in stress granule formation are linked to neurodegenerative diseases as a deficiency in forming stress granules may give conditions that promote the precipitation of misfolded proteins into nuclear aggregates (Daigle *et al*, 2013). In this respect, it is noteworthy that for several aggregation-prone proteins linked to neurodegenerative diseases localization to the nucleus is critical for their cytotoxic effect underscoring the importance of a proper functioning nuclear protein quality control in order to avoid protein aggregation in this compartment (Klement *et al*, 1998; Saudou *et al*, 1998).

Material and Methods

Plasmids

The GFP-TDP43 expression plasmid was generated by PCR amplifying the TDP43 open reading frame from mCherry-TDP43 using primers 5′-TAC AAG TCC GGA CTC AGA TCT CGA GGA ATG TCT GAA TAT ATT CGG GTA AC-3′ (forward primer) and 5′-CGC GGT ACC GTC GAC TGC AGA ATT CGG ATC CCT ACA TTC CC-3′ (reverse primer) and inserting it into *Eco*RI-digested EGFP-C1 (Clontech Laboratories) using NEBuilder HiFi DNA Assembly Master Mix (New England Biolabs) according to the manufacturer's

© 2022 The Authors

instructions. The mCherry-G3BP1 expression constructs were generated by PCR amplifying the G3BP1 constructs from GFP-G3BP1 mutants (kind gift from Gerald McInerney) using the primers 5′-GAT CTC GAG AAG CTT CGA ATT CAA TGG TGA TGG AGA AGC C-3′ (forward primer) and 5′-TCA GTT ATC TAG ATC CGG TGG ATC CTC AAA GGC GAT TATA CTC GTC -3′ (reverse primer for mCherry-G3BP1 and mCherry-G3BP1^{F33W}) or 5′- TCA GTT ATC TAG ATC CGG TGG ATC CTT ACT GCC GTG GCG CAA G-3′ (reverse primer mCherry-G3BP1$^{\Delta RGG}$) and inserting it into *Eco*RI and *Bam*HI-digested EGFP-C1 (Clontech Laboratories) using NEBuilder HiFi DNA Assembly Master Mix (New England Biolabs). The siRNA-resistant mCherry-G3BP1 expression constructs were generated by PCR amplifying these mCherry-G3BP1 constructs using primers 5′-CTC ATG AAG TTG ATA AGT CGG AAC TGA ATG ATT TCT TTC AAA GTT ATG-3′ (forward primer) and 5′-CAT AAC TTT GAA AGA AAT CAT TCA GTT CCG ACT TAT CAA CTT CAT GAG-3′ (reverse primer). The NLS-GFP-NLS-GFP-CL1 (NLS-GFP-CL1) and NES-GFP-NES-GFP-CL1 (NES-GFP-CL1) expression plasmids were a generous gift from Dr. Ron Kopito (Stanford University; Bennett *et al*, 2005). The ^{FLAG}Ub-Ataxin1-82Q plasmid (Verhoef *et al*, 2002) and α4-GFP plasmid (Dantuma *et al*, 2006) have been described previously.

Cell culture and transfections

The human melanoma MelJuSo (RRID:CVCL_1403) and human osteosarcoma U2OS (RRID:CVCL_0042) cell lines were cultured in DMEM + GlutaMAX (ThermoFisher Scientific) supplemented with 10% Fetal Bovine Serum (FBS) in a humidified chamber at 37°C and 5% CO_2. Cell lines are routinely tested for mycoplasma infection. The MelJuSo GFP-ODC, NLS-GFP-CL1, and NES-GFP-CL1 cell lines were created by transfection with corresponding expression plasmids using Lipofectamine 3000 (Invitrogen). Clones were selected in the presence of 0.5 mg/ml G418 (Gibco) and screened for GFP fluorescence by flow cytometry upon administration of the proteasome inhibitor MG132 (Sigma, M8699). Transient transfection was performed using Lipofectamine 3000 (Invitrogen) according to the manufacturer's instructions. For knockdown experiments, the following siRNA constructs were purchased from Invitrogen or Dharmacon: control siRNA (4390843), control siRNA (D-001210-01), G3BP1 (s19754), G3BP2 (s19207), HSP70 (M-005168–01), RNF4 (custom, 5′-GAA UGG ACG UCU CAU CGU U-3′) and PML (J-006547-07). Lipofectamine RNAiMax (#13778150; ThermoFisher Scientific) was used for the transfection of siRNA according to the manufacturer's instructions.

Heat shock and recovery

Cell culture plates were sealed with parafilm and placed in 43°C water bath for 30 min. After 30 min, the cells were placed in incubator at 37°C and 5% CO_2 for 2 h or 4 h for recovery.

Puromycin labelling

Puromycin (Sigma) was diluted to 5 μg/ml in prewarmed DMEM medium and added to cells before being subjected to heat shock in 43°C water bath for 30 min. For heat shock samples, cells were fixed with 4% PFA or lysed directly after heat shock. For recovery samples, puromycin was washed away with prewarmed DMEM medium (three times) and replaced with fresh medium, followed by incubation at 37°C and 5% CO_2 for the indicated recovery time. To study the role of nucleolar integrity was treated for 3 h with 4 μM actinomycin D (Sigma, A1410) before administration of puromycin.

Immunofluorescence

MelJuSo and U2OS cell lines were grown on coverslips overnight and treated as indicated. Cells were fixed using 4% paraformaldehyde (Sigma) for 15 min, permeabilized using 0.2% Triton-X100 in 1× phosphate-buffered saline (PBS) for 15 min, and blocked using 3% BSA in 1× PBS for 30 min. The primary antibodies were diluted in 0.1% Tween-20 in PBS and incubated overnight at 4°C. The following primary antibodies were used: anti-puromycin (MABE343; Sigma), anti-TIA1 (#140595; Abcam), anti-SUMO2/3 (#109005; Abcam), anti-PML (#53773; Abcam), anti-TDP43(#57105; Abcam), anti-Nucleolin (#22758; Abcam), anti-HSF1 (#4356; Cell Signaling Technology), anti-HSP70 (ADI-SPA-810; Enzo Life Sciences), anti-Ataxin-1 (#703273; ThermoFisher Scientific), anti-FLAG (F4042; Sigma). Goat anti-mouse or goat anti-rabbit IgG coupled to Alexa-Fluor 488, 546, or 647 (ThermoFisher Scientific) were used as secondary antibodies and diluted 1:500 in 0.1% Tween-20 in PBS. Nuclear counterstaining was performed using Hoechst 33342 (Molecular Probes) 1:5,000 in PBS for 15 min. Fixed cells were examined with a Zeiss LSM 880 confocal laser scanning microscope (Plan-Neofluar 63×/1.4 oil objective). Image processing was performed with FiJi and quantitative analyses were performed using cell profile software.

Immunoblotting

Equal amounts of cells were lysed in 1× LDS sample buffer (ThermoFisher Scientific) containing 10% NuPAGE reducing agent (NP0004; ThermoFisher Scientific). Lysates were boiled at 95°C for 5 min. Cell protein extracts were resolved by Bis–Tris polyacrylamide gel electrophoresis gels (NP0323, NP04343; ThermoFisher Scientific) and run in 1× MOPS buffer (NP0001; ThermoFisher Scientific). Proteins were transferred onto nitrocellulose membranes (#10600023; GE Healthcare) in a Tris–glycine transfer buffer containing 20% methanol. After blocking in Tris-buffered saline (TBS) containing 5% nonfat milk and 0.1% Tween-20, membranes were incubated with primary antibodies, washed with TBS-Tween-20 0.1%, and incubated with secondary goat anti-rabbit or goat anti-mouse antibodies linked to horse radish peroxidase (HRP) (NA934V, NA931V; GE Healthcare). Detection was performed by enhanced chemiluminescence (Amersham ECL reagents; GE Healthcare) on Medical X-ray films (Fujifilm). Alternatively, goat anti-mouse or goat anti-rabbit coupled to a far-red fluorescent dye (#926-68070, #926-32210; LI-COR) were used, and membranes scanned with an Odyssey scanner (LI-COR) and analyzed with Image Studio Lite analysis software version 5.2 (LI-COR). For quantification of western blots, band intensities were determined with ImageJ from independent experiments obtained with independent samples.

The following primary antibodies were used: anti-GFP (#290; Abcam), anti-Hsp70 (ADI-SPA-810; Enzo Life Sciences), anti-puromycin (MABE343; Sigma), anti-GAPDH (#9485; Abcam), anti-β-actin (#8226; Abcam), anti-HSF1 (#4356; Cell Signaling

 © 2022 The Authors

Technology), anti-G3BP1 (PA5-29455; ThermoFisher Scientific), anti-G3BP2 (#86135; Abcam), anti-SUMO2/3 (#109005; Abcam), anti TDP43 (#57105; Abcam), anti-RNF4 (AF7964; R&D Systems), anti-PML (#53773; Abcam), and anti-Ataxin-1 (#703273; ThermoFisher Scientific).

Solubility assay

The solubility assay was done essentially as described previously (Keiten-Schmitz *et al*, 2020). Parental U2OS or G3BP1/2 knockout U2OS cells were grown in 6 well plates and transfected with control siRNA or an siRNA directed against RNF4. Cells were either left untreated or exposed to 43°C for 30 min after which they were washed twice in cold PBS, followed by scraping the cells and collecting them in 150 µl lysis buffer on ice (50 mM Tris–pH 8.8), 100 mM NaCl, 5 mM $MgCl_2$, 0.5% NP-40, 2 mM DTT, 250 U/ml benzonase (Merck), 1 mM PMSF, 1× complete EDTA-free Protease Inhibitor Cocktail (Roche), 20 mM N Ethylmaleimide (NEM) (Sigma-Aldrich). After rotating the lysates for 30 min at 4°C, the samples were centrifugated (18,000 *g*, 4°C, 15 min). The supernatants were diluted with SDS–PAGE loading buffer and subsequently boiled for 5 min at 95°C (NP-40 soluble fraction). The pellets were resuspended in 50 µl of resuspension buffer (20 mM Tris, pH 8.0, 15 mM $MgCl_2$, 2 mM DTT, 250 U/ml benzonase, 1 mM PMSF, 1× Complete EDTA-free Protease Inhibitor Cocktail, and 20 mM NEM). After incubating the resuspended pellets for 30 min on ice, 50 µl of SDS buffer (5% SDS, 100 mM DTT, 10% glycerin, bromophenol blue) was added to the samples followed by boiling for 10 min at 95°C (SDS-soluble fraction).

High content microscopy

For live imaging, MelJuSo Ub-YFP were seeded in a 96-well plate (Falcon) at 4,000 cells per well and incubated overnight. After exposure to 43°C for 30 min, the medium was replaced by Leibovitz's L-15 medium (ThermoFisher Scientific) and 4 sites per well were imaged every 10 min using ImageXpress automated widefield microscope (Molecular Devices) with a 20× objective. A similar procedure was used for high-content imaging of fixed cells. The fluorescence intensities were automatically quantified by CellProfiler. The CellProfiler pipeline is available in the Appendix S1.

Rescue experiment

MelJuSo Ub-YFP cells were transfected with G3BP1/2 siRNA for 24 h followed by transiently transfected with siRNA-resistant expression plasmids: mCherry-C1, mCherry-G3BP1, mCherry-G3BP1^{F33W} or mCherry-G3BP1$^{\Delta RGG}$ for 48 h. After transfection, these cells were either left untreated (-HS), exposed to a heat shock for 30 min (HS), or exposed to a heat shock and followed by 4 h recovery (4 h Recovery). The yellow fluorescent intensities of the Ub-YFP reporter in mCherry-positive cells were quantified by CellProfiler.

Flow cytometry

For analysis of the reporter levels by flow cytometry, MelJuSo cells stably expressing NLS/NES-GFP-CL1 or parental U2OS and G3BP1/2 KO cells stably expressing Ub-YFP were seeded and treated in 12-well plates. Cells were harvested with trypsin and washed with PBS. Using the BD FACS Canto II (BD Biosciences), 20,000 cells were captured per group and cellular YFP and GFP intensities measured using the 488-nm laser. Data were analyzed using FlowJo (TreeStar V10). The reporter level was determined as the geometric mean of green or yellow fluorescent intensities for each group.

Proteasome activity assay

Parental U2OS and G3BP1/2 KO cells were treated as indicated and harvested in lysis buffer (25 mM HEPES pH 7.2, 50 mM NaCl, 1 mM MgCl2, 1 mM ATP, 1 mM DTT, 10% glycerol, 1% Triton X-100). After centrifugation, protein concentrations were measured with the protein assay dye reagent (Bio-Rad). Ten microgram proteins were mixed with 80 µl reaction buffer (lysis buffer without Triton X-100) and 10 µl suc-LLVY-AMC (Enzo, BML-P802-005) for a final concentration of 1 µM suc-LLVY-AMC. As a control, 100 nM of the proteasome inhibitor epoxomicin (Sigma) was added to the reaction mixture. Samples were analyzed in a microplate reader (FLUOStar OPTIMA) at 355 nm/460 nm every minute for 1 h.

Proteasome distribution

Parental U2OS and G3BP1/2 KO cells were transfected with GFP-tagged proteasome subunit α4 for 48 h and either left untreated or subjected to heat shock for 30 min and recovery for 4 h. The nucleus was stained by Hoechst before imaging by confocal microscopy.

SUMO2 deconjugase activity

Parental U2OS and G3BP1/2 KO cells were treated as indicated and harvested in lysis buffer (25 mM HEPES pH 7.2, 50 mM NaCl, 1 mM MgCl2, 1 mM ATP, 1 mM DTT, 10% glycerol, 1% Triton X-100). After centrifuge, protein concentrations were measured with the protein assay dye reagent (Bio-Rad). Ten microgram of protein were mixed with 80 µl reaction buffer (lysis buffer without Triton X-100) and 10 µl SUMO2-AMC (R&D Systems, UL-758) for a final concentration of 1 µM. As a control, 20 mM N-ethylmaleimide (NEM; ThermoFisher Scientific) to inhibit SUMO deconjugases. Samples were analyzed in a microplate reader (FLUOStar OPTIMA) at 355 nm/460 nm every minute for 1 h.

TUBE pulldown

After the indicated treatment, U2OS cells were harvested and lysed in RIPA buffer on ice for 30 min. After 10 min centrifugation at 4°C and 16,000 *g*, the supernatant was mixed with pre-equilibrium TUBE beads (Life Sensors) and incubated overnight at 4°C. The beads were washed three times with detergent-free RIPA buffer. After the final wash, 2× SDS sample buffer with the reducing agent was added to the beads before boiling samples for 10 min at 95°C. Prior to proceeding with SDS–PAGE and western blotting, beads were spun down at 2,500 *g* for 1 min. The supernatant was loaded on the gel.

Quantitative PCR

U2OS cells were subjected to heat shock or left untreated and collected by cell scraping at the indicated time points. Total RNA was

© 2022 The Authors

isolated with the RNeasy Mini Kit (QIAGEN) and subjected to reverse transcription to generate cDNA. Quantitative PCR (qPCR) was used to quantify the abundance of *HSP70* transcripts using SYBR Green dye. *GAPDH* was used to normalize qPCR signal and DMSO-treated controls were used to calculate the fold change of gene expression using the standard $\Delta\Delta C_t$ metric. Primer sequences were: *HSP70*: 5′-TTT GAG GGC ATC GAC TTC TAC A-3′ (forward primer) and 5′-CCA GGA CCA GGT CGT GAA TC-3′ (reverse primer); *GAPDH*: 5′-GGA GCG AGA TCC CTC CAA AAT-3′ (forward primer), 5′-GGC TGT TGT CAT ACT TCT CAT GG-3′ (reverse primer).

Statistical analysis

Statistical analyses were performed using GraphPad Prism version 8.3. To test for Gaussian distribution, the D'Agostino, and Pearson or Shapiro–Wilk normality test were used for smaller sample sizes. If the normality test was passed, data were analyzed by the Student's unpaired *t*-test or by ANOVA. If the data failed the test for normal distribution, statistical analysis was performed using the nonparametric Mann–Whitney test or the Kruskal-Wallis test for multiple comparisons, with the Dunnett's or Tukey's test to adjust for multiple comparisons. The Grubbs' test was used for the detection of outliers. Adjusted *P*-values are shown. Data are shown as mean ± standard deviation (S.D.) unless stated otherwise. The following *P*-values were considered significant: $^*P \leq 0.05$; $^{**}P \leq 0.01$.

Data availability

This study does not contain deposited data. Original data can be provided upon request.

Expanded View for this article is available online.

Acknowledgements
We thank Gerald McInerney, Marc Panas, Lifeng Liu, and members of the Dantuma lab for helpful input and the Biomedicum Imaging Center (BIC) and FACS facility for technical assistance. This work was supported by the Swedish Research Council (N.P.D. 2016-02479), the Swedish Cancer Society (N.P.D. CAN 2018/693), and the Joint Programme Neurodegenerative Diseases (JPND) (CureALS, 2015-06794, PP-829-050; S.A., N.P.D.). S.X. was supported by a scholarship from the Chinese Scholarship Council (CSC 201700260275). A.K.S. and M.E.G. were supported by research fellowships from the Deutsche Forschungsgemeinschaft (DFG) (SHE 2079/1-1; GI-1329/1-1).

Author contributions
Shanshan Xu: Conceptualization; formal analysis; validation; investigation; visualization; methodology; writing—review and editing. **Maria Gierisch:** Supervision; investigation; methodology; writing—review and editing. **Anna Katharina Schellhaus:** Investigation; writing—review and editing. **Ina Poser:** Investigation. **Simon Alberti:** Supervision. **Florian Salomons:** Conceptualization; supervision; investigation; methodology; writing—review and editing. **Nico P. Dantuma:** Conceptualization; formal analysis; supervision; funding acquisition; investigation; writing—original draft; project administration; writing—review and editing.

Disclosure and competing interests statement
The authors declare that they have no conflict of interest.

References

Andersson R, Eisele-Burger AM, Hanzen S, Vielfort K, Oling D, Eisele F, Johansson G, Gustafsson T, Kvint K, Nystrom T (2021) Differential role of cytosolic Hsp70s in longevity assurance and protein quality control. *PLoS Genet* 17: e1008951

Anisimov S, Takahashi M, Kakihana T, Katsuragi Y, Kitaura H, Zhang L, Kakita A, Fujii M (2019) G3BP1 inhibits ubiquitinated protein aggregations induced by p62 and USP10. *Sci Rep* 9: 12896

Arrasate M, Mitra S, Schweitzer ES, Segal MR, Finkbeiner S (2004) Inclusion body formation reduces levels of mutant huntingtin and the risk of neuronal death. *Nature* 431: 805–810

Askanas V, Engel WK, Nogalska A (2009) Inclusion body myositis: a degenerative muscle disease associated with intra-muscle fiber multi-protein aggregates, proteasome inhibition, endoplasmic reticulum stress and decreased lysosomal degradation. *Brain Pathol* 19: 493–506

Bence NF, Sampat RM, Kopito RR (2001) Impairment of the ubiquitin-proteasome system by protein aggregation. *Science* 292: 1552–1555

Bennett EJ, Bence NF, Jayakumar R, Kopito RR (2005) Global impairment of the ubiquitin-proteasome system by nuclear or cytoplasmic protein aggregates precedes inclusion body formation. *Mol Cell* 17: 351–365

Bennett EJ, Shaler TA, Woodman B, Ryu KY, Zaitseva TS, Becker CH, Bates GP, Schulman H, Kopito RR (2007) Global changes to the ubiquitin system in Huntington's disease. *Nature* 448: 704–708

Boeynaems S, Alberti S, Fawzi NL, Mittag T, Polymenidou M, Rousseau F, Schymkowitz J, Shorter J, Wolozin B, Van Den Bosch L *et al* (2018) Protein phase separation: a new phase in cell biology. *Trends Cell Biol* 28: 420–435

Ciechanover A, Brundin P (2003) The ubiquitin proteasome system in neuordegenerative diseases: sometimes the chicken, sometimes the egg. *Neuron* 40: 427–446

Daigle JG, Lanson NA Jr, Smith RB, Casci I, Maltare A, Monaghan J, Nichols CD, Kryndushkin D, Shewmaker F, Pandey UB (2013) RNA-binding ability of FUS regulates neurodegeneration, cytoplasmic mislocalization and incorporation into stress granules associated with FUS carrying ALS-linked mutations. *Hum Mol Genet* 22: 1193–1205

Dang F, Nie L, Wei W (2021) Ubiquitin signaling in cell cycle control and tumorigenesis. *Cell Death Differ* 28: 427–438

Dantuma NP, Groothuis TA, Salomons FA, Neefjes J (2006) A dynamic ubiquitin equilibrium couples proteasomal activity to chromatin remodeling. *J Cell Biol* 173: 19–26

Frottin F, Schueder F, Tiwary S, Gupta R, Korner R, Schlichthaerle T, Cox J, Jungmann R, Hartl FU, Hipp MS (2019) The nucleolus functions as a phase-separated protein quality control compartment. *Science* 365: 342–347

Ganassi M, Mateju D, Bigi I, Mediani L, Poser I, Lee HO, Seguin SJ, Morelli FF, Vinet J, Leo G *et al* (2016) A surveillance function of the HSPB8-BAG3-HSP70 chaperone complex ensures stress granule integrity and dynamism. *Mol Cell* 63: 796–810

Guo L, Giasson BI, Glavis-Bloom A, Brewer MD, Shorter J, Gitler AD, Yang X (2014) A cellular system that degrades misfolded proteins and protects against neurodegeneration. *Mol Cell* 55: 15–30

Hendriks IA, Treffers LW, Verlaan-de Vries M, Olsen JV, Vertegaal ACO (2015) SUMO-2 orchestrates chromatin modifiers in response to DNA damage. *Cell Rep* 10: 1778–1791

Holmberg CI, Hietakangas V, Mikhailov A, Rantanen JO, Kallio M, Meinander A, Hellman J, Morrice N, MacKintosh C, Morimoto RI *et al* (2001) Phosphorylation of serine 230 promotes inducible transcriptional activity of heat shock factor 1. *EMBO J* 20: 3800–3810

Jolly C, Morimoto R, Robert-Nicoud M, Vourc'h C (1997) HSF1 transcription factor concentrates in nuclear foci during heat shock: relationship with transcription sites. *J Cell Sci* 110: 2935–2941

 © 2022 The Authors

Kedersha N, Panas MD, Achorn CA, Lyons S, Tisdale S, Hickman T, Thomas M, Lieberman J, McInerney GM, Ivanov P *et al* (2016) G3BP-Caprin1-USP10 complexes mediate stress granule condensation and associate with 40 S subunits. *J Cell Biol* 212: 845 – 860

Keiten-Schmitz J, Wagner K, Piller T, Kaulich M, Alberti S, Muller S (2020) The nuclear SUMO-targeted ubiquitin quality control network regulates the dynamics of cytoplasmic stress granules. *Mol Cell* 79: 54 – 67.e7

Klement IA, Skinner PJ, Kaytor MD, Yi H, Hersch SM, Clark HB, Zoghbi HY, Orr HT (1998) Ataxin-1 nuclear localization and aggregation: role in polyglutamine-induced disease in SCA1 transgenic mice. *Cell* 95: 41 – 53

Kopito RR (2000) Aggresomes, inclusion bodies and protein aggregation. *Trends Cell Biol* 10: 524 – 530

Kotoglou P, Kalaitzakis A, Vezyraki P, Tzavaras T, Michalis LK, Dantzer F, Jung JU, Angelidis C (2009) Hsp70 translocates to the nuclei and nucleoli, binds to XRCC1 and PARP-1, and protects HeLa cells from single-strand DNA breaks. *Cell Stress Chaperones* 14: 391 – 406

Kwon YT, Ciechanover A (2017) The ubiquitin code in the ubiquitin-proteasome system and autophagy. *Trends Biochem Sci* 42: 873 – 886

Marmor-Kollet H, Siany A, Kedersha N, Knafo N, Rivkin N, Danino YM, Moens TG, Olender T, Sheban D, Cohen N *et al* (2020) Spatiotemporal proteomic analysis of stress granule disassembly using APEX reveals regulation by SUMOylation and links to ALS pathogenesis. *Mol Cell* 80: 876 – 891.e6

Masser AE, Ciccarelli M, Andreasson C (2020) Hsf1 on a leash – controlling the heat shock response by chaperone titration. *Exp Cell Res* 396: 112246

Mateju D, Franzmann TM, Patel A, Kopach A, Boczek EE, Maharana S, Lee HO, Carra S, Hyman AA, Alberti S (2017) An aberrant phase transition of stress granules triggered by misfolded protein and prevented by chaperone function. *EMBO J* 36: 1669 – 1687

Maynard CJ, Bottcher C, Ortega Z, Smith R, Florea BI, Diaz-Hernandez M, Brundin P, Overkleeft HS, Li JY, Lucas JJ *et al* (2009) Accumulation of ubiquitin conjugates in a polyglutamine disease model occurs without global ubiquitin/proteasome system impairment. *Proc Natl Acad Sci USA* 106: 13986 – 13991

McGurk L, Gomes E, Guo L, Mojsilovic-Petrovic J, Tran V, Kalb RG, Shorter J, Bonini NM (2018) Poly(ADP-ribose) prevents pathological phase separation of TDP-43 by promoting liquid Demixing and stress granule localization. *Mol Cell* 71: 703 – 717.e9

Mediani L, Guillen-Boixet J, Alberti S, Carra S (2019a) Nucleoli and Promyelocytic leukemia protein (PML) bodies are phase separated nuclear protein quality control compartments for misfolded proteins. *Mol Cell Oncol* 6: e1415624

Mediani L, Guillen-Boixet J, Vinet J, Franzmann TM, Bigi I, Mateju D, Carra AD, Morelli FF, Tiago T, Poser I *et al* (2019b) Defective ribosomal products challenge nuclear function by impairing nuclear condensate dynamics and immobilizing ubiquitin. *EMBO J* 38: e101341

Menendez-Benito V, Verhoef LG, Masucci MG, Dantuma NP (2005) Endoplasmic reticulum stress compromises the ubiquitin-proteasome system. *Hum Mol Genet* 14: 2787 – 2799

Meyer C, Garzia A, Morozov P, Molina H, Tuschl T (2020) The G3BP1-family-USP10 Deubiquitinase complex rescues Ubiquitinated 40 S subunits of ribosomes stalled in translation from lysosomal degradation. *Mol Cell* 77: e1195

Molliex A, Temirov J, Lee J, Coughlin M, Kanagaraj AP, Kim HJ, Mittag T, Taylor JP (2015) Phase separation by low complexity domains promotes stress granule assembly and drives pathological fibrillization. *Cell* 163: 123 – 133

Nacerddine K, Lehembre F, Bhaumik M, Artus J, Cohen-Tannoudji M, Babinet C, Pandolfi PP, Dejean A (2005) The SUMO pathway is essential for nuclear integrity and chromosome segregation in mice. *Dev Cell* 9: 769 – 779

Nollen EA, Salomons FA, Brunsting JF, van der Want JJ, Sibon OC, Kampinga HH (2001) Dynamic changes in the localization of thermally unfolded nuclear proteins associated with chaperone-dependent protection. *Proc Natl Acad Sci USA* 98: 12038 – 12043

Ortega Z, Diaz-Hernandez M, Maynard CJ, Hernandez F, Dantuma NP, Lucas JJ (2010) Acute polyglutamine expression in inducible mouse model unravels ubiquitin/proteasome system impairment and permanent recovery attributable to aggregate formation. *J Neurosci* 30: 3675 – 3688

Panas MD, Schulte T, Thaa B, Sandalova T, Kedersha N, Achour A, McInerney GM (2015) Viral and cellular proteins containing FGDF motifs bind G3BP to block stress granule formation. *PLoS Pathog* 11: e1004659

Protter DSW, Parker R (2016) Principles and properties of stress granules. *Trends Cell Biol* 26: 668 – 679

Reits EA, Vos JC, Gromme M, Neefjes J (2000) The major substrates for TAP *in vivo* are derived from newly synthesized proteins. *Nature* 404: 774 – 778

Riggs CL, Kedersha N, Ivanov P, Anderson P (2020) Mammalian stress granules and P bodies at a glance. *J Cell Sci* 133: jcs242487

Sala AJ, Bott LC, Morimoto RI (2017) Shaping proteostasis at the cellular, tissue, and organismal level. *J Cell Biol* 216: 1231 – 1241

Salomons FA, Menendez-Benito V, Bottcher C, McCray BA, Taylor JP, Dantuma NP (2009) Selective accumulation of aggregation-prone proteasome substrates in response to proteotoxic stress. *Mol Cell Biol* 29: 1774 – 1785

Saudou F, Finkbeiner S, Devys D, Greenberg ME (1998) Huntingtin acts in the nucleus to induce apoptosis but death does not correlate with the formation of intranuclear inclusions. *Cell* 95: 55 – 66

Schmidt EK, Clavarino G, Ceppi M, Pierre P (2009) SUnSET, a nonradioactive method to monitor protein synthesis. *Nat Methods* 6: 275 – 277

Schubert U, Anton LC, Gibbs J, Norbury CC, Yewdell JW, Bennink JR (2000) Rapid degradation of a large fraction of newly synthesized proteins by proteasomes. *Nature* 404: 770 – 774

Shao S, Hegde RS (2016) Target selection during protein quality control. *Trends Biochem Sci* 41: 124 – 137

Sherman MY, Goldberg AL (2001) Cellular defenses against unfolded proteins: a cell biologist thinks about neurodegenerative diseases. *Neuron* 29: 15 – 32

Skinner PJ, Koshy BT, Cummings CJ, Klement IA, Helin K, Servadio A, Zoghbi HY, Orr HT (1997) Ataxin-1 with an expanded glutamine tract alters nuclear matrix-associated structures. *Nature* 389: 971 – 974

Soncini C, Berdo I, Draetta G (2001) Ras-GAP SH3 domain binding protein (G3BP) is a modulator of USP10, a novel human ubiquitin specific protease. *Oncogene* 20: 3869 – 3879

Sreedharan J, Blair IP, Tripathi VB, Hu X, Vance C, Rogelj B, Ackerley S, Durnall JC, Williams KL, Buratti E *et al* (2008) TDP-43 mutations in familial and sporadic amyotrophic lateral sclerosis. *Science* 319: 1668 – 1672

Tatham MH, Geoffroy MC, Shen L, Plechanovova A, Hattersley N, Jaffray EG, Palvimo JJ, Hay RT (2008) RNF4 is a poly-SUMO-specific E3 ubiquitin ligase required for arsenic-induced PML degradation. *Nat Cell Biol* 10: 538 – 546

Verhoef LG, Lindsten K, Masucci MG, Dantuma NP (2002) Aggregate formation inhibits proteasomal degradation of polyglutamine proteins. *Hum Mol Genet* 11: 2689 – 2700

Zhang H, Shao S, Zeng Y, Wang X, Qin Y, Ren Q, Xiang S, Wang Y, Xiao J, Sun Y (2022) Reversible phase separation of HSF1 is required for an acute transcriptional response during heat shock. *Nat Cell Biol* 24: 340 – 352

Zoghbi HY, Orr HT (2000) Glutamine repeats and neurodegeneration. *Annu Rev Neurosci* 23: 217 – 247

License: This is an open access article under the terms of the Creative Commons Attribution License, which permits use, distribution and reproduction in any medium, provided the original work is properly cited.

Expanded View Figures

Figure EV1. G3BP1/2 deficiency impairs the UPS in thermally stressed cells.

A MelJuSo cells expressing Ub-YFP transfected with control siRNA or G3BP1 and G3BP2 siRNA were either left untreated (− heat shock) or exposed to 43°C for 30 min and followed for 4 h after the heat shock (Recovery). Cell lysates were analyzed by immunoblot with G3BP1, G3BP2, and GAPDH antibodies. Quantification of the G3BP1 and G3BP2 band densities. Data represent the mean ± SD ($n = 3$ independent experiments, Student's unpaired t-test, $^{**}P < 0.01$).
B MelJuSo cells expressing Ub-YFP transfected with control siRNA or G3BP1 and G3BP2 siRNA were subjected to 43°C heat shock for 30 min and followed by automated high-content imaging every 10 min for 4 h. Representative images are shown. Scale bar is 20 μm.
C Fluorescence micrographs of MelJuSo cells expressing YFP-CL1, which were either left untreated (− heat shock) or exposed to 43°C for 30 min and followed for 4 h after the heat shock (HS+ 4 h Rec.). Scale bar is 10 μm.
D Representative images of MelJuSo expressing Ub-YFP, which were transfected with G3BP1/2 siRNA and either transfected with mCherry-C1, mCherry-G3BP1, mCherry-G3BP1F33W or mCherry-G3BP1ΔRGG plasmids and subjected to heat shock. Scale bar is 20 μm.
E Fluorescence micrographs of MelJuSo cells expressing GFP-ODC, which were either left untreated (− heat shock) or exposed to 43°C for 30 min and followed for 4 h after heat shock (HS+ 4 h Rec.). Scale bar is 20 μm.
F Quantification of (E) of > 500 cells per group. Normalized to siControl "- heat shock." The frequency and distribution of the relative fluorescent intensity per cell are shown as violin plots. The solid lines in each distribution represent the median, and dash lines represent the upper and lower interquartile range limits ($n = 3$ independent experiments, > 1,000 cells analyzed per condition).

 © 2022 The Authors

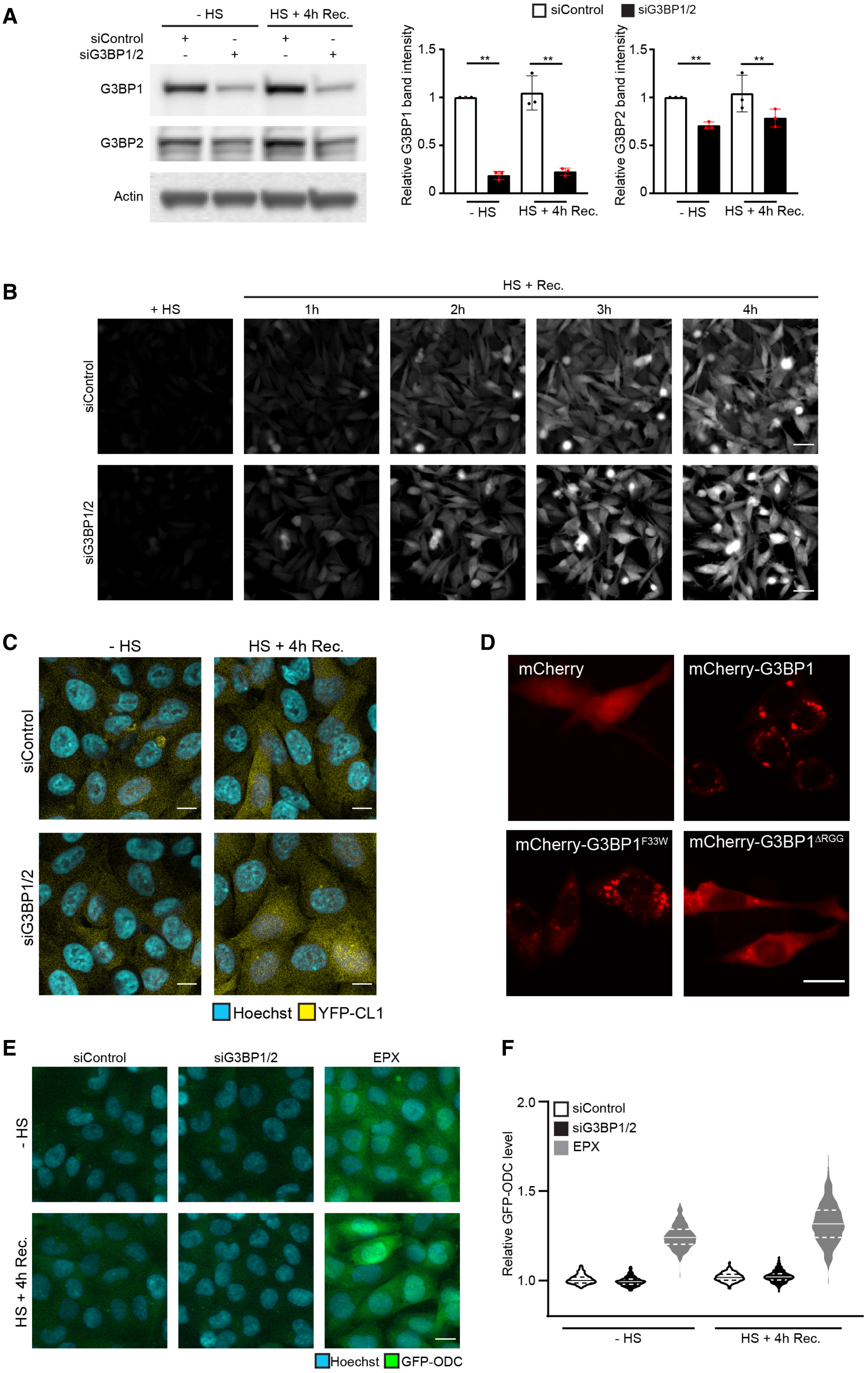
A
- HS
HS + 4h Rec.
siControl
siG3BP1/2
G3BP1
G3BP2
Actin
siControl
siG3BP1/2
Relative G3BP1 band intensity
Relative G3BP2 band intensity
B
+ HS
HS + Rec.
1h
2h
3h
4h
siControl
siG3BP1/2
C
- HS
HS + 4h Rec.
Hoechst
YFP-CL1
D
mCherry
mCherry-G3BP1
mCherry-G3BP1F33W
mCherry-G3BP1ΔRGG
E
siControl
siG3BP1/2
EPX
- HS
HS + 4h Rec.
Hoechst
GFP-ODC
F
siControl
siG3BP1/2
EPX
Relative GFP-ODC level

Figure EV1.

Figure EV2. Nuclear UPS impairment and nucleolar DRiP accumulation in G3BP1/2 knockout cells.

A Flow cytometric detection of fluorescent intensities MelJuSo cells expressing NLS/NES-GFP-CL1, which were either left untreated (− HS), or exposed to 43°C for 30 min and followed for 4 h after heat shock (HS+ Rec.). Data represent the mean ± SD (n = 3 independent experiments, Student's unpaired t-test, *$P < 0.05$).

B Flow cytometric detection of fluorescent intensities in parental U2OS and G3BP1/2 knockout (KO) cells expressing Ub-YFP, which were either left untreated (− HS), or exposed to 43°C for 30 min and followed for 4 h after heat shock (Rec.). Data represent the mean ± SD (n = 3 independent experiments, Student's unpaired t-test, *$P < 0.05$).

C Fluorescence micrographs of parental U2OS and G3BP1/2 KO cells expressing Ub-YFP, which were either left untreated (− HS), exposed to 43°C for 30 min (HS), or followed for 4 h after heat shock (HS+ Recovery). Stress granules are visualized by immunostaining for TIA1. Scale bar is 10 µm.

D Representative confocal images of immunofluorescent staining of puromycin-labeled proteins in parental and G3BP1/2 KO U2OS cells exposed to a heat shock (+ HS; left panel), after 1 h recovery (HS + 1 h Rec.; middle panel), and after 2 h recovery (HS + 2 h Rec.; right panel). Scale bar is 10 µm.

E Quantification of nucleolar localization of puromycin labeled proteins in parental and G3BP1/2 KO U2OS cells in (D). Data represent the mean ± SD (n = 3 independent experiments, > 50 cells analyzed per condition, Kruskal-Wallis test, **$P < 0.01$, n.s.—not significant).

 © 2022 The Authors

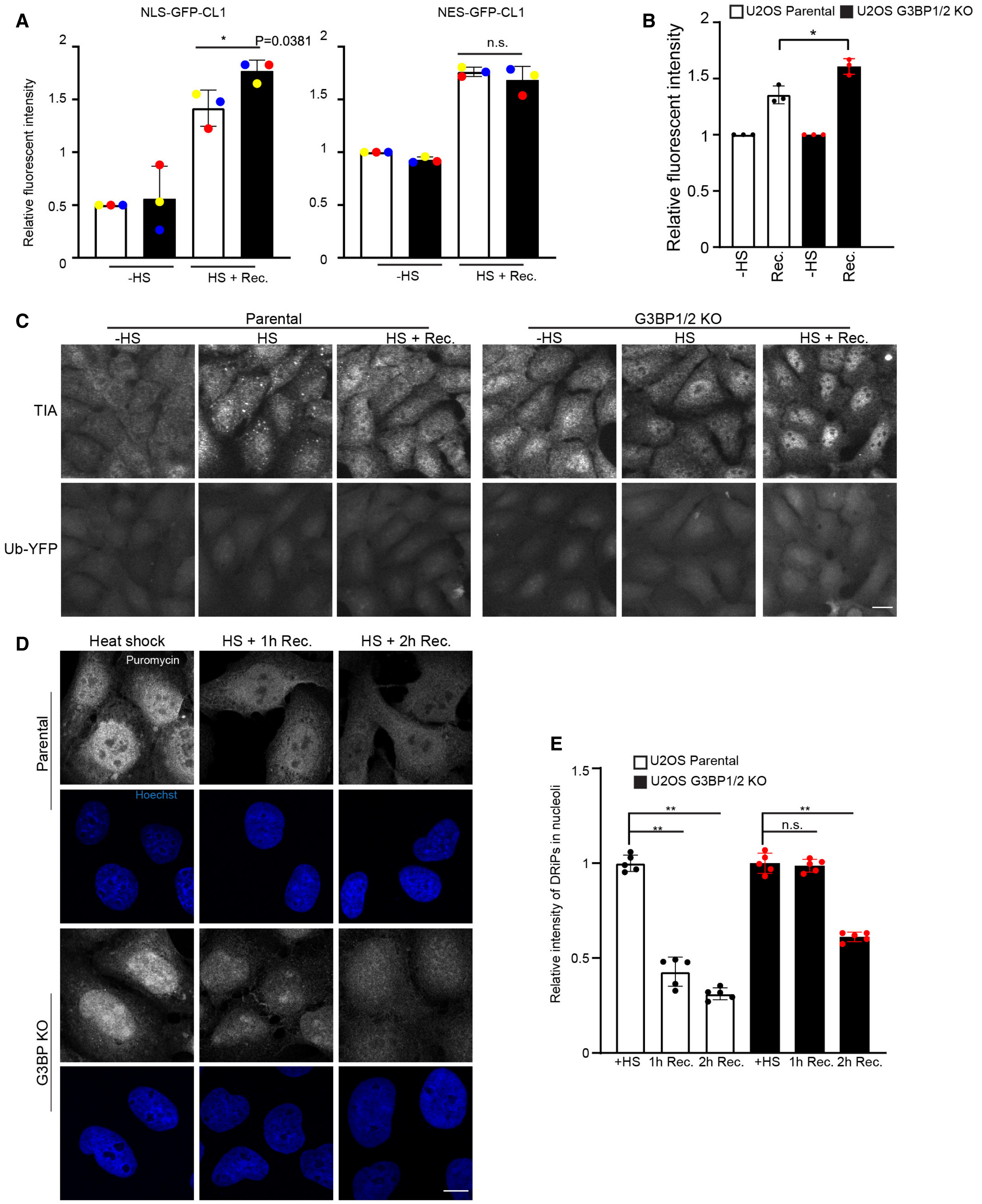
A
NLS-GFP-CL1
NES-GFP-CL1
Relative fluorescent intensity
* P=0.0381
n.s.
-HS
HS + Rec.
B
U2OS Parental
U2OS G3BP1/2 KO
Relative fluorescent intensity
*
-HS
Rec.
-HS
Rec.
C
Parental
G3BP1/2 KO
-HS
HS
HS + Rec.
TIA
Ub-YFP
D
Heat shock
HS + 1h Rec.
HS + 2h Rec.
Puromycin
Hoechst
Parental
G3BP KO
E
U2OS Parental
U2OS G3BP1/2 KO
Relative intensity of DRiPs in nucleoli
**
**
**
n.s.
+HS
1h Rec.
2h Rec.
+HS
1h Rec.
2h Rec.

Figure EV2.

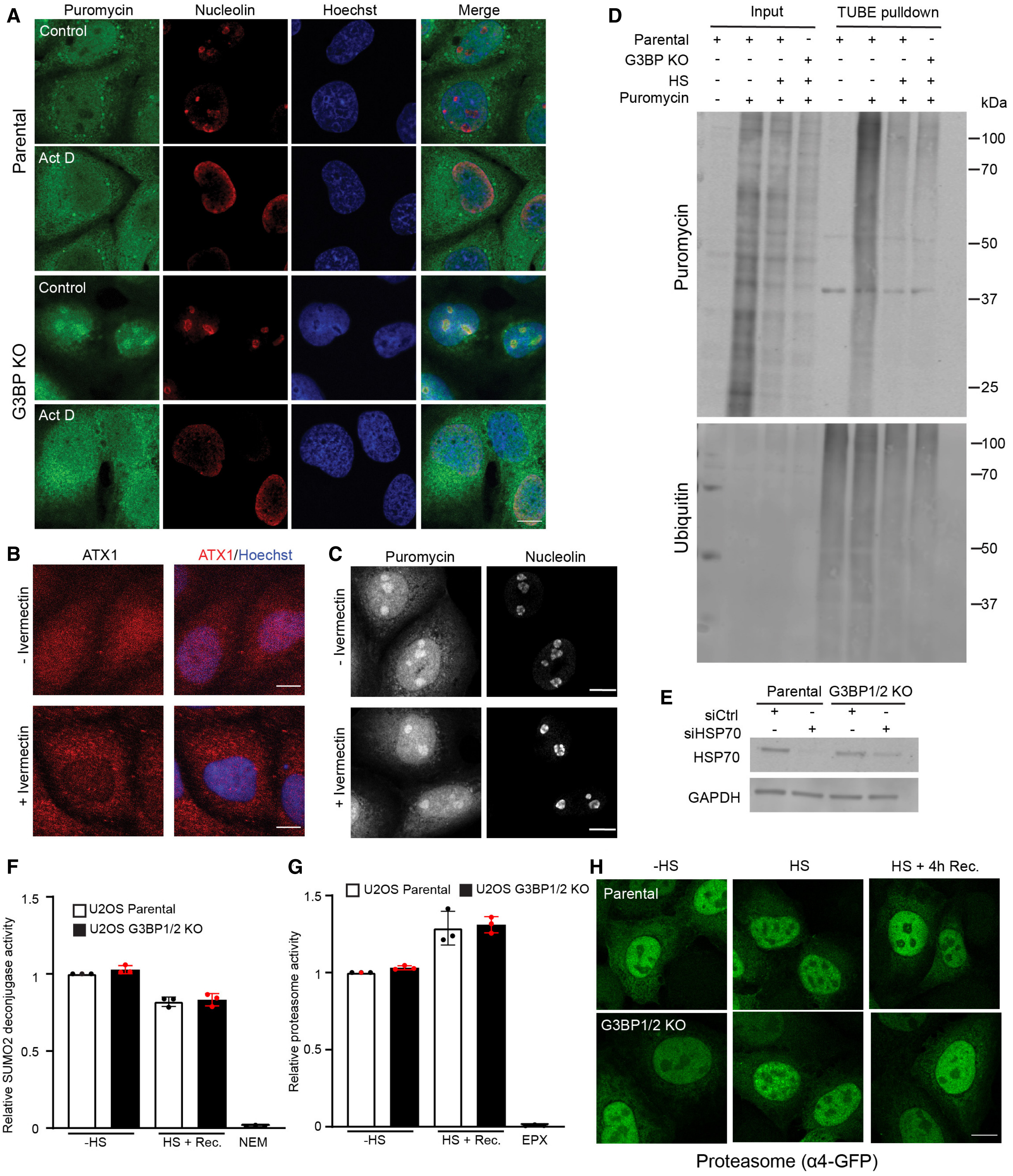

Figure EV3.

© 2022 The Authors

Figure EV3. DRiPs and proteasome activity in thermally stressed G3BP1/2 knockout cells.

A Representative confocal images of immunofluorescent staining of the nucleolar marker nucleolin and puromycin-labeled proteins in U2OS cells pretreated with or without 4 μM Actinomycin D for 3 h, subjected to a heat shock. Scale bar is 20 μm.

B U2OS G3BP1/2 knockout cells were treated with or without 25 μM ivermectin for 2 h. The ataxin1 was visualized by immunostaining. Scale bar is 20 μm.

C U2OS G3BP1/2 knockout cells were treated with or without 25 μM ivermectin for 2 h before being exposed to 43°C heat shock in a 5 μg/ml puromycin-containing medium. The DRiPs and nucleoli were visualized by immunostaining for puromycin and nucleolin, respectively. Scale bar is 20 μm.

D Immunoblot of ubiquitylated DRiPs by TUBE pulldown of lysates from parental U2OS and G3BP1/2 knockout cells after heat shock.

E Immunoblot of HSP70 in parental and G3BP1/2 knockout U2OS cells transfected with HSP70 siRNA for 72 h.

F Parental U2OS and G3BP1/2 knockout cells were either left untreated (− HS), or exposed to 43°C for 30 min and followed for 4 h after heat shock (HS + Rec.). The SUMO2 deconjugase activity was detected by following the conversion of the fluorogenic SUMO2- AMC substrate over 1 h. As a control, 20 mM NEM was added to the reaction mixture to inhibit SUMO2 deconjugases. Data represent the mean ± SD ($n = 3$ independent experiments, Student's unpaired t-test).

G Parental U2OS and G3BP1/2 knockout cells were either left untreated (− HS), or exposed to 43°C for 30 min and followed for 4 h after heat shock (HS + Rec.). The chymotrypsin-like activity (β5 subunit) of the proteasome was detected by following the conversion of the fluorogenic Suc-LLVY-AMC substrate over 1 h. As a control, 100 nM epoxomicin (EPX) was added to the reaction mixture to inhibit proteasome activity. Data represent the mean ± SD ($n = 3$ independent experiments, Student's unpaired t-test).

H Representative images of parental U2OS and G3BP1/2 knockout cells transiently transfected with α4-GFP, which were either left untreated (− HS), or exposed to 43°C for 30 min and followed for 4 h after heat shock (HS + 4 h Rec.). Scale bar is 20 μm.

© 2022 The Authors

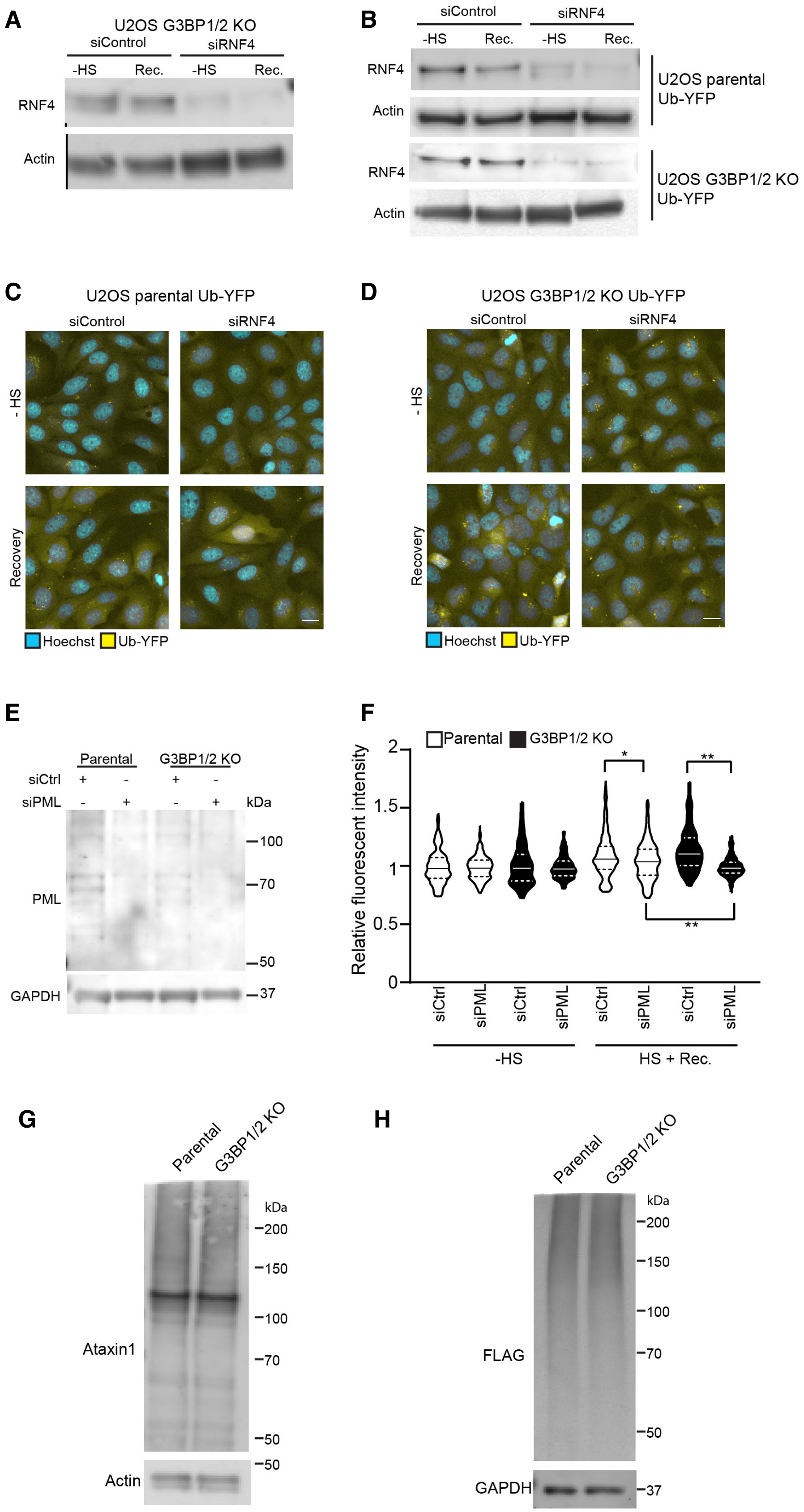

Figure EV4. Inhibition of SUMO-targeted ubiquitylation partially restores UPS activity in thermally stressed G3BP1/2 knockout cells.

A U2OS G3BP1/2 knockout cells expressing Ub-YFP were transfected with control siRNA or RNF4 siRNA. Cell lysates were analyzed by immunoblot with RNF4 and Actin antibodies.

B Parental U2OS and G3BP1/2 KO cells expressing Ub-YFP were transfected with control siRNA or RNF4 siRNA. Cell lysates were analyzed by immunoblot with RNF4 and Actin antibodies.

C Fluorescence micrographs of parental U2OS cells stably expressing Ub-YFP, which had been transfected with control or RNF4 siRNA. Cells were either left untreated (− heat shock), or exposed to 43°C for 30 min and followed for 4 h after heat shock (Recovery). Scale bar is 10 μm.

D Fluorescence micrographs of U2OS G3BP1/2 KO cells stably expressing Ub-YFP, which had been transfected with control or RNF4 siRNA. Cells were either left untreated (− heat shock), or exposed to 43°C for 30 min and followed for 4 h after heat shock (Recovery). Scale bar is 10 μm.

E Immunoblotting for PML in control (siControl) and PML-depleted (siPML) parental and G3BP1/2 KO U2OS cells.

F Quantification of mean cellular YFP fluorescence intensities in U2OS expressing Ub-YFP with and without PML siRNA transfection. The YFP fluorescence is normalized to untreated control cells. The frequency and distribution of the relative fluorescence intensities per cell are shown as violin plots. The solid lines in each distribution represent the median, and dash lines represent the upper and lower interquartile range limits (n = 3 independent experiments, > 1,000 cells analyzed per condition, Kruskal-Wallis test, *P < 0.05, **P < 0.01).

G Immunoblot of ataxin-1 in parental and G3BP1/2 KO U2OS cells transfected with ^{FLAG}Ub-Ataxin-1-82Q.

H Immunoblot of ^{FLAG}Ubiquitin in parental and G3BP1/2 KO U2OS cells transfected with ^{FLAG}Ub-Ataxin-1-82Q.

© 2022 The Authors
