# Supplementary material for: Cytosolic stress granules relieve the ubiquitin‐proteasome system in the nuclear compartment
Source: EMBO J. 2022 Dec 27;42(3):e111802. doi: 10.15252/embj.2022111802 (PMC9890234; doi:10.15252/embj.2022111802)
Supplement: Supplementary file 4 [file EMBJ-42-e111802-s004.pdf]

THE EMBO JOURNAL

Cytosolic stress granules relieve the ubiquitin-proteasome system in the nuclear compartment

Shanshan Xu, Maria Gierisch, Anna Schellhaus, Ina Poser, Simon Alberti, Florian Salomons, and Nico P. Dantuma
DOI: 10.15252/embj.2022111802

Corresponding author(s): Nico P. Dantuma (nico.dantuma@ki.se)

Review Timeline:

Submission Date:	1st Jun 22
Editorial Decision:	8th Jul 22
Revision Received:	12th Oct 22
Editorial Decision:	21st Nov 22
Revision Received:	25th Nov 22
Accepted:	1st Dec 22

Editor: Hartmut Vodermaier

Transaction Report:

(Note: With the exception of the correction of typographical or spelling errors that could be a source of ambiguity, letters and reports are not edited. Depending on transfer agreements, referee reports obtained elsewhere may or may not be included in this compilation. Referee reports are anonymous unless the Referee chooses to sign their reports.)

1st Editorial Decision **8th Jul 2022**

Thank you for submitting your study on stress granules and nuclear quality control for our consideration. With some delay, for which I would like to apologize, we have now received a full set of reports from three expert referees, copied below for your information. As you will see, all referees find your results, to varying degrees, potentially interesting, but at the same time also note that not all conclusions are decisively supported, that alternative explanations not always tested, and that several aspects would be strengthened with deeper follow-up insights.

Should you be able to adequately strengthen the criticized aspects and extend the analyses in the direction suggested by the overlapping comments of the reviewers, we would be interested in pursuing a revised version of the study further for publication. Since it is our policy to consider only a single round of major revision, it might in this case be helpful to discuss already during the early stages of your revision work how the various raised concerns could be addressed. I would therefore invite you to carefully consider the reports together with your co-workers, and to send me a tentative point-by-point response via email, which could serve as the basis for further discussion via email or online call. I should add that we could also offer extension of the default three-months revision period if needed, with our 'scooping protection' (meaning that competing work appearing elsewhere in the meantime will not affect our considerations of your study) remaining valid also throughout this extension.

Referee #1:

Summary

This manuscript contains a number of interesting observations, but is very hard to read and follow as too many seemingly unrelated aspects are presented here and connected. The manuscript starts with an interesting observation that depletion of G3BP1/2 that impairs stress granule (SG) formation leads to an impairment of UPS activity. It is however unclear if UPS components are not present or dysregulated or simply not functional in the nucleus upon G3BP1/2. The authors did not discuss any other role of G3BP1/2 than its requirement for SG formation. Next, they observed that defective ribosomal products accumulate in the nucleolus when SG formation is impaired and that Hsp70 is involved in the re-distribution and this activates the heat shock response. The next section on SUMO-targeted Ubiquitin ligases (RNF4) is not obvious and is disconnected from the results presented up to this point. The SG-deficient cells exhibit a higher number of SUMO2/3 in the nucleoli, but are cleared faster than in control cells in the recovery period. They could show that TDP-43 that is targeted by RNF4 accumulates in epoxomicin treated cells. To test the connection between the RNF4 and the proteasome they depleted RNF4 and could observe differences in the accumulation of Ub-YFP in the recovery period of SG-deficient cells vs control. And finally, they challenged SG-deficient cells with the expression of aggregation-prone ataxin and observed the expected accumulation of ataxin aggregates in G3BP-1/2 depleted cells.
Overall, the authors performed a high number of experiments and obtained interesting data. However, correlation vs causality is not clear and needs to be established by e.g. alternative experimental strategies to validate the data and also rescue experiments by e.g. re-introducing G3BP1/2 would substantially strengthen the manuscript.

Major comments:

• Figure 1A: There are TIA1 positive signals also upon siG3BP1/2 - but in the nucleus. The authors should comment on the implications of this signal.

• Figure 2A: NLS-GFP-CL1 forms foci in the nucleus upon siG3BP1/2. To confirm that the overall levels are also elevated due to impaired nuclear UPS activity I suggest to validate the accumulation of NLS-GFP-CL1 by western blot. Furthermore, the authors should test, if the nuclear proteasome levels, its subcellular distribution or its activity is changed upon siG3BP1/2.
• The data depicted in EV4 are not convincing. There is no obvious difference in the Ub-YFP levels between the G3BP1/2 knockout and control strain. There is also no quantification. This experiment needs to be validated by another method e.g. western blot of Ub-YFP levels.
• The authors argue that Hsp70 supports the transport of DRiPs to the nucleus. I would be cautious with such a conclusion as Hsp70 depleted cells will be impaired in numerous cellular pathways and the observed differences to the control might be an indirect consequence.
• The observation that Hsp70 co-localizes with the DRiPs in the nucleoli and that this is accompanied with an activation of the HSR is not surprising. It is established that Hsp70 co-localizes with nucleolar protein aggregates and is required for the nucleolar protein quality control: Azkanas et al., 2019; Kotoglou et al. 2009; Frottin et al., 2019

Minor comments:

• What is the reason for using MelJuSo cells for knockdown and U2OS cells for knockout experiments?
• The manuscript contains numerous orthographical and grammatical mistakes and needs careful proof-reading.
• Figure description and legends do not always fit e.g. loading control is GAPDH, but stated as actin in the text. Figure legend for Fig 2A/B lists NLS-GFP-CL1 for both conditions. Figure 2C/D quantification of YFP instead of GFP.
• Figures can be improved by providing more information on what certain graphs depict: e.g. the depicted fluorescence (e.g. EV1B); protein band identity in Fig 5E, 7A., y-axis labelling in Fig 5B, color assignment for PML in the merge of Fig 6C missing
• Quantification of siRNA-mediated knockdown of G3BP1/2 is missing in EV1A
• Scale bars are either missing or certainly not correct in Figures 1A vs C, 4A, EV3
• Figure 2: The data for + HS are missing
• Materials & Method section is not complete: there is no information on the puromycin-labelling of newly synthesized proteins.

Referee #2:

The study of Xu et al. examine the consequences of disrupting of stress granule formation on the functionality of the ubiquitin-proteasome (UPS) system. This was achieved by analyzing the localization and stability of different UPS substrates in WT and G3BP1/2-deficient cells. The authors demonstrate that G3BP1/2-deficient cells have less UPS activity during the recovery phase from heat stress, as demonstrated by the accumulation of different unstable model proteins: Ub-YFP, NLS-GFP-CL1, and puromycin-released nascent chains (referred to as defective ribosomal producs, DRiPs). NLS-GFP-CL1 (nuclear) and DRiPs (cytoplasmic) proteins partially relocate to the nucleoli of G3BP1/2-deficient cells after heat shock. Both stress granules and nucleoli can function as a storage compartment for misfolded and/or aggregation-prone proteins; the experiments reported here indicate that in the absence of the former some defective proteins can instead relocate to the nucleolus. The authors propose that enhanced targeting of misfolded proteins for proteasomal degradation in the nuclear compartment overwhelms the nuclear UPS and aggravates UPS impairment. Finally, they show that G3BP1/2-KO cells present more and larger inclusions of mutant ataxin-1, demonstrating that loss of cytosolic stress granule impinges on nuclear proteostasis.
The study addresses important molecular mechanisms related to stress response and maintenance of proteostasis in the context stress and/or disease, and many of the reported observations are highly relevant and novel. I think they significantly advance the fast growing body of knowledge concerning the role of phase separation in stress response, and therefore would be of interest to a broad audience. Although the manuscript is well written, the mechanistic links and the overall physiological consequences of these observations can be further clarified/discussed.
1. Current literature indicate that metastable proteins relocate to the nucleoli as a conjugate with Hsp70 as a protective mechanism to avoid aggregation and to facilitate their clearance upon stress recovery. A key finding of this study is that cytoplasmic DRiPs relocate to nucleoli in the absence of stress granules. The authors propose that nuclear accumulation of DRiPs may be unfortunate as it will transfer the burden of these potentially toxic proteins to the nuclear compartment, which is less well equipped to eliminate them. While Fig. 4 shows that they relocate to the nucleus, it is not completely clear to me whether they accumulate and/or persist longer during heat shock recovery in G3BP1/2-KO cells. Quantitative time-course data would be required to support this conclusion.
Moreover, wouldn't DRiPs retention in the nucleoli nevertheless serve as a protective role against their aggregation? It would be interesting to test whether nucleolar disassembly with actinomycin D (in the context of WT vs. G3BP1/2-KO, prior to DRiPs relocation) would promote DRiPs degradation in the cytoplasm, where the UPS is still active, or whether it would promote their aggregation. It would also be important to show if such interventions would have an effect on stress survival.
2. The authors demonstrate that SUMOylated proteins (including TDP41 and PML) present a dramatically different behavior than DRiPs: after heat shock, they accumulate in the nucleoli in WT cells but not in G3BP1/2-KO. Also, in G3BP1/2-KO SUMOylated protein levels decrease via UPS degradation in the recovery phase, while the UPS substrate Ub-YFP accumulates. Disruption of ubiquitin-dependent degradation of SUMOylated proteins restored degradation of Ub-YFP in G3BP1/2-KO cells. The authors

propose that enhanced targeting of misfolded proteins for proteasomal degradation in the nuclear compartment overwhelms the nuclear UPS and aggravates UPS impairment. While I agree with the general conclusion, I find some observations rather surprising, and in need of further mechanistic discussion/clarification. The efficient degradation of SUMOylated proteins and GFP-ODC hint that the activity of nuclear proteasomes per se is not significantly compromised. If this is the case, does it mean that SUMOylated proteins and Ub-YFP compete for the same ubiquitination enzymes? Or do the authors believe that certain types of substrates are favored for proteasomal degradation (as indicated in Fig. 8C)? I would find it rather surprising that an aggregation-prone protein such as TDP43 would have an advantage over other proteins. Another possibility is that typically cytosolic substrates (as the case of newly synthesized proteins, DRiPs) might not encounter the adequate quality control pathways in the nucleus. Here, analysis of the polyubiquitination levels of DRiPs and/or Ub-YFP could help to clarify the underlying molecular mechanisms.

3. The material and methods section needs significant improvement. There is no description of how images were quantified. In Fig. 5C, for example, I can visually pinpoint a large number of co-localized PML and SUMO 2/3 puncta in parental cells, however the corresponding quantification shows a percentage of SUMO2/3-positive PML puncta close to 0. Moreover, it is not completely clear if the reporters used were transient transfections, polyclonal or monoclonal cell lines. For example, how the Ub-YFP reporter was introduced in parental and G3BP1/2 knockout U2OS cells is not described, and appropriate control experiments excluding quantitative differences in reporter expression levels are not shown. I also find that the description of the ataxin-1 construct insufficient to understand the experiment without consulting its original reference. Also here the authors don't show a quantification of the control demonstrating that expression levels were equal in parental and G3BP1/2 KO cells. There is also no description of the employed puromycin treatments, nor of the experiment in Fig. 1B. The methods section explains the rationale for the use of a number of different statistical tests; nevertheless, the employed tests should be mentioned in Figure legends.

4. Please correct the Figure references in lines 219, 220, and 319. Axis of Fig. 5B has the wrong label.

Referee #3:

In the presented manuscript Xu and colleagues provide evidence on how the ubiquitin-proteasome system (UPS) functions in cells that are unable to form stress granules under elevated temperature. Interestingly, the inability of cells to form stress granules negatively impacts the functionality of UPS within the cell nucleus. While misfolded proteins are accumulated at stress granules in the cytoplasm under the heat shock, they relocalize to nucleoli in stress granules-deficient cells. Their nuclear redistribution is accompanied by relocalization and enhanced degradation of SUMOylated proteins. Additionally, depletion of the SUMO-targeted ubiquitin ligase RNF4, which marks SUMOylated misfolded proteins for proteasomal degradation, restores the UPS functionality to some degree in nucleoli of stress granule-deficient cells. The authors proposed that stress granules function as sequestration sites for misfolded proteins to prevent their accumulation in the nucleus, where they would otherwise affect the functional nuclear proteome. This is an interesting manuscript but some results are sometimes less clear to make claims fully justified. Further experimental proof would be needed to support some of the claims. Some conclusions do not appear to be fully supported by the presented data. Specific comments are listed below:

Major concerns:

Figure 1A. It is established that TIA1 cytotoxic granule-associated RNA binding protein also regulates transcription and pre-mRNA splicing in the nucleus. In control cells and in fully recovered cells TIA1 is localized in some nuclear condensates. What are they? They are localized outside nucleoli. It seems that they are less visible or disorganized under heat shock in control cells. However, they are clearly noticeable in G3BP1/2-depleted cells under heat shock in addition to the nucleolar staining. Are they PML NBs? They look positive for the Ub-YFP in Fig. 1C.

Figure 2E. I can clearly see positively stained nucleoli with NLS-GFP-CL1 reporter in control cells as far as I can tell. Are these images taken under the same confocal settings for control and G3BP1/2-depleted cells? GFP-positive G3BP1/2-depleted cells look generally brighter but these data are not convincing.

I believe the authors are fully aware that heat shock drastically affects nucleolar pre-rRNA synthesis and consequently the nucleolar functional sub-organization mediated by LLPS. It's obvious that nucleoli in HSP70-depleted cells are affected more severally than unaffected nucleoli in the puromycin-treated control cells. Therefore, their status for colocalization comparison is not the same. In other words, HSP70 is important for the nucleolar function under heat shock and its actions might be unrelated to UPS.

While the basal level of SUMOylated proteins can be detected in various cell types, it can significantly increase after proper stimulation. It has been proposed that the SUMO-2/-3 pathway may constitute an element of the cellular response to environmental stress to globally increase the SUMOylation level. Is it possible that the depletion of G3BP1/2 brings novel stress conditions associated with increased SUMOylation?

The authors should evaluate changes in protein SUMOylation by measuring endogenous levels of SUMO1 and SUMO2/3. They should check and compare the status and the presence of active SUMO-specific proteases (SENPs) which remove SUMO from protein substrates in G3BP1/2-depleted cells versus control cells. In addition, they should use cysteine protease inhibitor PR619 to check and compare the status of SUMOylation in control and coilin KD HeLa cells.

Free SUMO can be recycled for another round of protein conjugation. Do you detect any significant changes in free SUMO levels in G3BP1/2-depleted cells vs controls?

In my opinion, the authors ignore the role of nuclear condensates called PML nuclear bodies. It is well established that the SUMOylated proteins accumulate predominantly bound to chromatin and localized to PML nuclear bodies. Figure 5C suggests the presence of PML NBs in parental and in G3BP1/2-depleted cells. However, the authors completely avoided the function of

PML NBs in UPS within the nucleus in their story. They should expand on this issue and provide additional evidence of the function of PML NBs in G3BP1/2-depleted cells.
Does the size of aggregates in nucleoli vary during the recovery from heat shock in G3BP1/2-depleted cells?

Xu et al, Rebuttal letter

Rebuttal EMBOJ-2022-111802 "Cytosolic stress granules relieve the ubiquitin-proteasome system in the nuclear compartment"

We would like to thank the reviewers for their constructive comments. We feel that addressing the concerns of the reviewers has considerably strengthened our study. Additional experiments have been included in the manuscript and where requested we have edited the text and clarified issues that were unclear. Changes are marked in red font in the revised manuscript.

Reviewer #1: "This manuscript contains a number of interesting observations, but is very hard to read and follow as too many seemingly unrelated aspects are presented here and connected. The manuscript starts with an interesting observation that depletion of G3BP1/2 that impairs stress granule (SG) formation leads to an impairment of UPS activity. It is however unclear if UPS components are not present or dysregulated or simply not functional in the nucleus upon G3BP1/2. The authors did not discuss any other role of G3BP1/2 than its requirement for SG formation. Next, they observed that defective ribosomal products accumulate in the nucleolus when SG formation is impaired and that Hsp70 is involved in the re-distribution and this activates the heat shock response. The next section on SUMO-targeted Ubiquitin ligases (RNF4) is not obvious and is disconnected from the results presented up to this point. The SG-deficient cells exhibit a higher number of SUMO2/3 in the nucleoli, but are cleared faster than in control cells in the recovery period. They could show that TDP-43 that is targeted by RNF4 accumulates in epoxomicin treated cells. To test the connection between the RNF4 and the proteasome they depleted RNF4 and could observe differences in the accumulation of Ub-YFP in the recovery period of SG-deficient cells vs control. And finally, they challenged SG-deficient cells with the expression of aggregation-prone ataxin and observed the expected accumulation of ataxin aggregates in G3BP-1/2 depleted cells.
Overall, the authors performed a high number of experiments and obtained interesting data. However, correlation vs causality is not clear and needs to be established by e.g. alternative experimental strategies to validate the data and also rescue experiments by e.g. re-introducing G3BP1/2 would substantially strengthen the manuscript."

Authors: We have performed rescue experiments with siRNA-resistant G3BP1. Besides validating that the observed UPS impairment is indeed caused to G3BP1 deficiency, we also used this opportunity to have a closer look if the UPS impairment is caused by the inability of G3BP1-deficient cells to form stress granules. In addition to its critical role in stress granule formation it has been reported that G3BP1 performs other activities through its interaction with the deubiquitinase USP10 (Anisimov et al, 2019; Kedersha et al, 2016; Meyer et al, 2020; Soncini et al, 2001). To distinguish whether the effect on the UPS is caused by an inability to form stress granules or, alternatively, through an unrelated USP10-dependent activity of G3BP1, we rescued G3BP1/2-depleted cells with siRNA resistant mCherry-tagged wild-type G3BP1, mutant G3BP1^{F33W} (proficient for stress granule formation but deficient for USP10 binding) and mutant G3BP1$^{\Delta RGG}$ (deficient for stress granule formation but proficient for USP10 binding) (Panas et al, 2015). In line with our hypothesis, we found that the stress granule proficient mCherry-wtG3BP1 and mCherry- G3BP1^{F33W} prevented the aggravated UPS impairment of thermally stressed G3BP1/2 knockout cells whereas the stress granule deficient G3BP1$^{\Delta RGG}$ mutant did not rescue. These data are now shown in new Fig 1G and Fig EV1D and discussed in lines 160-173.

Reviewer 1:

Major comments:

“• Figure 1A: There are TIA1 positive signals also upon siG3BP1/2 - but in the nucleus. The authors should comment on the implications of this signal.”

Authors: This is a sharp observation of the reviewer. Indeed, whereas the G3BP1/2-depleted cells did not display the typical dot-like cytosolic TIA1 pattern, indicative for stress granules, there was the appearance of some nuclear dots in these cells. As we felt that the characterization of these nuclear speckles lied outside of the scope of our study, we did not investigate this in detail. We have now performed a co-staining for TIA1 with SUMO and PML but have been unable to detect co-localization with these markers in response to thermal stress. As these structures remain unidentified, we do not want to draw conclusions about their relevance for the UPS impairment but have noted their presence now in the result section (lines 137-141).

“• Figure 2A: NLS-GFP-CL1 forms foci in the nucleus upon siG3BP1/2. To confirm that the overall levels are also elevated due to impaired nuclear UPS activity I suggest to validate the accumulation of NLS-GFP-CL1 by western blot.”

Authors: Because of the semiquantitative nature of western blotting, we opted to quantify the total levels of NLS-GFP-CL1 and NES-GFP-CL1 by flow cytometry instead as this provides a more quantitative readout. Three independent experiments confirmed that there was a significant increase in the levels of NLS-GFP-CL1 in thermally stressed cells whereas no significant difference was observed for the NES-GFP-CL1 reporter. These data are shown in new Fig EV2A and discussed in lines 195-198.

“Furthermore, the authors should test, if the nuclear proteasome levels, its subcellular distribution or its activity is changed upon siG3BP1/2.”

Authors: We have analyzed the localization of proteasomes in control and G3BP1/2 knock-out cells by expressing a GFP-tagged proteasome subunit. The catalytic activity of the proteasome was analyzed using the fluorogenic substrate suc-LLVY-AMC. No apparent or significant difference were observed between control and G3BP1/2 knockout cells. These data are shown in Fig EV3G and H and discussed in lines 336-339.

“• The data depicted in EV4 are not convincing. There is no obvious difference in the Ub-YFP levels between the G3BP1/2 knockout and control strain. There is also no quantification. This experiment needs to be validated by another method e.g. western blot of Ub-YFP levels.”

Authors: We agree that this may be hard to evaluate from the micrographs but we think that the effect may have been somewhat masked by the Hoechst signal. Therefore, we have removed the Hoechst signal and converted the images to grey scale. Moreover, we have complemented this data set with a flow cytometric analysis of the fluorescent intensities of these cells, which shows a significant increase in the levels of Ub-YFP in G3BP1/2 knock-out cells 4 hours after thermal stress. This is shown in new Fig. EV2B and C and discussed in lines 225-227.

“• The authors argue that Hsp70 supports the transport of DRiPs to the nucleus. I would be cautious with such a conclusion as Hsp70 depleted cells will be impaired in numerous cellular pathways and the observed differences to the control might be an indirect consequence.”

Authors: We concluded in our manuscript that the localization of the DRiPs is dependent on Hsp70. Even after careful reading, we have not been able to detect a statement in our manuscript that would imply that Hsp70 transports or support the transport of DRiPs. We are aware of the fact that indirect effects of Hsp70 depletion may play a role but feel that is not relevant at this point for our conclusions. Our data conclusively shows that nucleolar localization is dependent on Hsp70.

"• The observation that Hsp70 co-localizes with the DRiPs in the nucleoli and that this is accompanied with an activation of the HSR is not surprising. It is established that Hsp70 co-localizes with nucleolar protein aggregates and is required for the nucleolar protein quality control: Azkanas et al., 2019; Kotoglou et al. 2009; Frottin et al., 2019"

Authors: We did not claim that this was unexpected and cited papers that are consistent with this sequence of events. We believe that the novelty of our study lies in part in the fact that cytosolic stress granules prevent/suppress this nuclear response, not the nature of the nuclear response. We included the papers by Askanas et al and Kotoglou et al. The paper by Frottin et al was already cited in this context. See lines 458-460.

Minor comments:

"• What is the reason for using MelJuSo cells for knockdown and U2OS cells for knockout experiments?"

Authors: We originally started the study with MelJuSo because we have used these cells earlier in studies on the functionality of the UPS. MelJuSo cell lines expressing various types of reporter substrates had already been developed by us and had been characterized. Later we started to work also with U2OS cells as U2OS cells in which the G3BP1 and G3BP2 genes have been deleted were available and had already been characterized by other groups. We believe that the reproducibility of our key findings in two independent cell lines using different approaches (knock-down and knock-out) strengthens our study and supports that we have revealed a common mechanism.

"• The manuscript contains numerous orthographical and grammatical mistakes and needs careful proof-reading."

Authors: We have carefully checked the manuscript for typos and grammatical errors.

"• Figure description and legends do not always fit e.g. loading control is GAPDH, but stated as actin in the text. Figure legend for Fig 2A/B lists NLS-GFP-CL1 for both conditions. Figure 2C/D quantification of YFP instead of GFP."

Authors: We have added explanatory details to the figures and corrected mistakes in the figure legends.

"• Figures can be improved by providing more information on what certain graphs depict: e.g. the depicted fluorescence (e.g. EV1B); protein band identity in Fig 5E, 7A., y-axis labelling in Fig 5B, color assignment for PML in the merge of Fig 6C missing"

Authors: We have added explanatory details to the figures and corrected mistakes.

"• Quantification of siRNA-mediated knockdown of G3BP1/2 is missing in EV1A"

Authors: We have added a quantification of the western blot. Note that quantification of western blot is semi-quantitative. Most important is that depletion inhibits the formation of stress granules which is a functional readout for G3BP1/2 depletion.

"• Scale bars are either missing or certainly not correct in Figures 1A vs C, 4A, EV3"

Authors: Scale bars have been edited and corrected.

"• Figure 2: The data for + HS are missing"

Authors: We have now included also the +HS images but omitted the Hoechst staining because of space limitation. See Fig 2A.

"• Materials & Method section is not complete: there is no information on the puromycin-labelling of newly synthesized proteins."

Authors: We have complemented the Materials & Method section with the missing information as well as included experimental methods that were performed for the revision. Changes are marked in red font.

Referee #2: "The study of Xu et al. examine the consequences of disrupting of stress granule formation on the functionality of the ubiquitin-proteasome (UPS) system. This was achieved by analyzing the localization and stability of different UPS substrates in WT and G3BP1/2-deficient cells. The authors demonstrate that G3BP1/2-deficient cells have less UPS activity during the recovery phase from heat stress, as demonstrated by the accumulation of different unstable model proteins: Ub-YFP, NLS-GFP-CL1, and puromycin-released nascent chains (referred to as defective ribosomal producs, DRiPs). NLS-GFP-CL1 (nuclear) and DRiPs (cytoplasmic) proteins partially relocate to the nucleoli of G3BP1/2-deficient cells after heat shock. Both stress granules and nucleoli can function as a storage compartment for misfolded and/or aggregation-prone proteins; the experiments reported here indicate that in the absence of the former some defective proteins can instead relocate to the nucleolus. The authors propose that enhanced targeting of misfolded proteins for proteasomal degradation in the nuclear compartment overwhelms the nuclear UPS and aggravates UPS impairment. Finally, they show that G3BP1/2-KO cells present more and larger inclusions of mutant ataxin-1, demonstrating that loss of cytosolic stress granule impinges on nuclear proteostasis.
The study addresses important molecular mechanisms related to stress response and maintenance of proteostasis in the context stress and/or disease, and many of the reported observations are highly relevant and novel. I think they significantly advance the fast growing body of knowledge concerning the role of phase separation in stress response, and therefore would be of interest to a broad audience. Although the manuscript is well written, the mechanistic links and the overall physiological consequences of these observations can be further clarified/discussed."

"1. Current literature indicate that metastable proteins relocate to the nucleoli as a conjugate with Hsp70 as a protective mechanism to avoid aggregation and to facilitate their clearance upon stress recovery. A key finding of this study is that cytoplasmic DRiPs relocate to nucleoli in the absence of stress granules. The authors propose that nuclear accumulation of DRiPs may be unfortunate as it will transfer the burden of these potentially toxic proteins to the nuclear compartment, which is less well equipped to eliminate them. While Fig. 4 shows that they relocate to the nucleus, it is not completely clear to me whether they accumulate and/or persist longer during heat shock recovery in G3BP1/2-KO cells. Quantitative time-course data would be required to support this conclusion. Moreover, wouldn't DRiPs retention in the nucleoli nevertheless serve as a protective role against their aggregation?"

Authors: In the Fig 4A of the original manuscript, we showed already that DRiPs could still be detected 1 hr after heat shock in the nucleoli of stress granule-deficient cells whereas the stress granules had been resolved at that time point in control cells. This indeed suggests that there is a more persistent sequestration of DRiPs in stress granule deficient cells. We have now included in new Fig EV2D and E micrographs and quantification of the localization of DRiPs in nucleoli at 0, 1 hr and 2 hrs post heat shock. This point is now also emphasized in lines 241-244.

"It would be interesting to test whether nucleolar disassembly with actinomycin D (in the context of WT vs. G3BP1/2-KO, prior to DRiPs relocation) would promote DRiPs degradation in the cytoplasm, where the UPS is still active, or whether it would promote their aggregation. It would also be important to show if such interventions would have an effect on stress survival."

Authors: This is an interesting suggestion. We have tested the effect of actinomycin D and found that the disintegration of nucleoli by actinomycin D was accompanied by dispersing of the DRiPs. This shows that the integrity of nucleoli is important for the sequestration of DRiPs. Actinomycin D inhibits transcription and since the steady-state levels of the short-lived UPS reporters are extremely sensitive to changes in transcription, it is unfortunately technically complicated to explore the functional status of the UPS in the presence of actinomycin D. Regarding cell viability, we used a mild heat shock with little, if any effect, on cell survival. Under the conditions that we use, we do not think that it would be very meaningful to look at the effect on stress survival. The effect of actinomycin D on DRiP sequestration is show in new Fig EV3A and discussed at lines 247-250.

"2. The authors demonstrate that SUMOylated proteins (including TDP41 and PML) present a dramatically different behavior than DRiPs: after heat shock, they accumulate in the nucleoli in WT cells but not in G3BP1/2-KO. Also, in G3BP1/2-KO SUMOylated protein levels decrease via UPS degradation in the recovery phase, while the UPS substrate Ub-YFP accumulates. Disruption of ubiquitin-dependent degradation of SUMOylated proteins restored degradation of Ub-YFP in G3BP1/2-KO cells. The authors propose that enhanced targeting of misfolded proteins for proteasomal degradation in the nuclear compartment overwhelms the nuclear UPS and aggravates UPS impairment. While I agree with the general conclusion, I find some observations rather surprising, and in need of further mechanistic discussion/clarification. The efficient degradation of SUMOylated proteins and GFP-ODC hint that the activity of nuclear proteasomes per se is not significantly compromised."

Authors: Indeed, we do not have any indications that the activity of proteasomes is affected. We have compared the localization and proteolytic activity in stress granule-proficient and -deficient

cells and did not detect any differences. See also our reply to Reviewer #1. These new data are now shown in Fig EV3G and H and are discussed at lines 336-339.

"If this is the case, does it mean that SUMOylated proteins and Ub-YFP compete for the same ubiquitination enzymes? Or do the authors believe that certain types of substrates are favored for proteasomal degradation (as indicated in Fig. 8C)? I would find it rather surprising that an aggregation-prone protein such as TDP43 would have an advantage over other proteins. Another possibility is that typically cytosolic substrates (as the case of newly synthesized proteins, DRiPs) might not encounter the adequate quality control pathways in the nucleus. Here, analysis of the polyubiquitination levels of DRiPs and/or Ub-YFP could help to clarify the underlying molecular mechanisms."

Authors: Our data indeed suggest that certain substrates are prioritized during proteotoxic stress. This is an interesting but complicated issue that will require further in-depth studies. We have data comparing ubiquitylation of DRiPs in stress granule-proficient and -deficient cells after heat shock, which did not reveal a striking difference. These data can be included in new Fig EV3D and is discussed at lines 257-260.

"3. The material and methods section needs significant improvement. There is no description of how images were quantified. In Fig. 5C, for example, I can visually pinpoint a large number of co-localized PML and SUMO 2/3 puncta in parental cells, however the corresponding quantification shows a percentage of SUMO2/3-positive PML puncta close to 0. Moreover, it is not completely clear if the reporters used were transient transfections, polyclonal or monoclonal cell lines. For example, how the Ub-YFP reporter was introduced in parental and G3BP1/2 knockout U2OS cells is not described, and appropriate control experiments excluding quantitative differences in reporter expression levels are not shown. I also find that the description of the ataxin-1 construct insufficient to understand the experiment without consulting its original reference. Also here the authors don't show a quantification of the control demonstrating that expression levels were equal in parental and G3BP1/2 KO cells. There is also no description of the employed puromycin treatments, nor of the experiment in Fig. 1B. The methods section explains the rationale for the use of a number of different statistical tests; nevertheless, the employed tests should be mentioned in Figure legends."

Authors: We would like to thank the reviewer for pointing out these shortcomings. We have added the requested information in the text. We have clarified the ^{FLAG}Ub-Ataxin-1 construct in more detail and cited an earlier paper in which this technique is described. Moreover, we included western blots of with FLAG antibody (detecting ^{FLAG}Ub conjugates) and ataxin-1 antibody and both blots show comparable expression levels. These data are now shown on in new Fig EV4G and H and mentioned at lines 405-406.

4. Please correct the Figure references in lines 219, 220, and 319. Axis of Fig. 5B has the wrong label.

Authors: This has been corrected.

Referee #3:

"In the presented manuscript Xu and colleagues provide evidence on how the ubiquitin-proteasome system (UPS) functions in cells that are unable to form stress granules under elevated temperature. Interestingly, the inability of cells to form stress granules negatively impacts the functionality of UPS within the cell nucleus. While misfolded proteins are accumulated at stress granules in the cytoplasm under the heat shock, they relocalize to nucleoli in stress granules-deficient cells. Their nuclear redistribution is accompanied by relocalization and enhanced degradation of SUMOylated proteins. Additionally, depletion of the SUMO-targeted ubiquitin ligase RNF4, which marks SUMOylated misfolded proteins for proteasomal degradation, restores the UPS functionality to some degree in nucleoli of stress granule-deficient cells. The authors proposed that stress granules function as sequestration sites for misfolded proteins to prevent their accumulation in the nucleus, where they would otherwise affect the functional nuclear proteome. This is an interesting manuscript but some results are sometimes less clear to make claims fully justified. Further experimental proof would be needed to support some of the claims. Some conclusions do not appear to be fully supported by the presented data. Specific comments are listed below:"

"Major concerns:
Figure 1A. It is established that TIA1 cytotoxic granule-associated RNA binding protein also regulates transcription and pre-mRNA splicing in the nucleus. In control cells and in fully recovered cells TIA1 is localized in some nuclear condensates. What are they? They are localized outside nucleoli. It seems that they are less visible or disorganized under heat shock in control cells. However, they are clearly noticeable in G3BP1/2-depleted cells under heat shock in addition to the nucleolar staining. Are they PML NBs? They look positive for the Ub-YFP in Fig. 1C."

Authors: This is an intriguing observation. We have performed co-staining for PML and SUMO but did not detect co-localization with the TIA1-positive nuclei in stressed and recovered control and G3BP1/2-depleted MelJuSo cells. As we feel that the characterization of these nuclei TIA1 punctate, though interesting, lie outside of the scope of this study we have not included these negative data.

Figure 2E. I can clearly see positively stained nucleoli with NLS-GFP-CL1 reporter in control cells as far as I can tell. Are these images taken under the same confocal settings for control and G3BP1/2-depleted cells? GFP-positive G3BP1/2-depleted cells look generally brighter but these data are not convincing.

Authors: The reviewer is correct that a slight increase in nucleolar NLS-GFP-CL1 can also be detected in thermally stressed control cells in Fig. 2E. This is also clear from the quantification shown in Fig 2F as the ratio between NLS-GFP-CL1 in nucleoli and nucleoplasm in control cells is also larger than 1 indicative for higher nucleolar levels. However, the levels of nucleolar NLS-GFP-CL1 are clearly increased for the G3BP1/2 depleted cells as can be seen in the micrographs and accompanying quantification. The images were captured with the same settings as is true for all experiments where we compare levels or localization of proteins in stress granule-proficient and -deficient cells. We emphasize now that we are referring to a quantitative, not a qualitative, difference at lines 201-203.

"I believe the authors are fully aware that heat shock drastically affects nucleolar pre-rRNA synthesis and consequently the nucleolar functional sub-organization mediated by LLPS. It's obvious that nucleoli in HSP70-depleted cells are affected more severally than unaffected nucleoli in the

puromycin-treated control cells. Therefore, their status for colocalization comparison is not the same. In other words, HSP70 is important for the nucleolar function under heat shock and its actions might be unrelated to UPS."

Authors: We conclude that Hsp70 is required for nucleolar localization of DRiPs. Based on our data, it is not possible to conclude whether this is a direct or indirect effect. We refrain from drawing conclusions about the reason for the Hsp70 dependency.

"While the basal level of SUMOylated proteins can be detected in various cell types, it can significantly increase after proper stimulation. It has been proposed that the SUMO-2/-3 pathway may constitute an element of the cellular response to environmental stress to globally increase the SUMOylation level. Is it possible that the depletion of G3BP1/2 brings novel stress conditions associated with increased SUMOylation?"

Authors: This is an interesting suggestion. In our western blots we have not detected any striking differences in the levels of SUMO2/3 conjugates in stress granule-proficient and -deficient cells under physiological conditions or directly after heat shock (compare lane 1 and 2 with lane 5 and 6 in Fig 5E). Differences are only observed during the recovery phase, which is in line with our model that the clearance of the SUMO2/3 modified proteins is accelerated in stress granule-deficient cells. We have no reason to believe that G3BP1/2 deficiency elicits a stress response that causes an increase in SUMO2/3 conjugates.

"The authors should evaluate changes in protein SUMOylation by measuring endogenous levels of SUMO1 and SUMO2/3."

Authors: We have focused on SUMO2/3 as SUMO1 bears little relevance to our study. Even when optimizing the conditions for western blotting, we were unable to detect a pool of free SUMO2/3.

"They should check and compare the status and the presence of active SUMO-specific proteases (SENPs) which remove SUMO from protein substrates in G3BP1/2-depleted cells versus control cells."

Authors: We have tested the levels of SUMO deconjugase activity using the fluorogenic substrate SUMO-AMC. The SUMO deconjugase activity was reduced during the recovery phase in control and G3BP1/2 knockout cells, which may contribute to the increase levels of SUMO2/3 conjugates in response to stress. Importantly, there was no difference between the SUMO deconjugase activity in control and G3BP1/2 knockout cells indicating that changes in SUMO deconjugation is unlikely to be responsible for the increased clearance of SUMO2/3 conjugates in G3BP1/2 knockout cells when recovering from proteotoxic stress. These data are shown in new Fig EV3F and discussed at lines 331-336.

"In addition, they should use cysteine protease inhibitor PR619 to check and compare the status of SUMOylation in control and coilin KD HeLa cells."

Authors: The rational for this experiment was not clear to us. Moreover, PR619 does not only inhibit SUMOylation but also ubiquitylation which will complicate the interpretation of the data. It is unclear to us why coilin knockdown HeLa cells should be used.

"Free SUMO can be recycled for another round of protein conjugation. Do you detect any significant changes in free SUMO levels in G3BP1/2-depleted cells vs controls?"

Authors: As mentioned above, we have been unable to detect free SUMO2/3 suggesting that the bulk of SUMO2/3 is incorporated in chains under control and stress conditions.

"In my opinion, the authors ignore the role of nuclear condensates called PML nuclear bodies. It is well established that the SUMOylated proteins accumulate predominantly bound to chromatin and localized to PML nuclear bodies. Figure 5C suggests the presence of PML NBs in parental and in G3BP1/2-depleted cells. However, the authors completely avoided the function of PML NBs in UPS within the nucleus in their story. They should expand on this issue and provide additional evidence of the function of PML NBs in G3BP1/2-depleted cells."

Authors: We agree that the role of PML in nuclear bodies could be interesting as it is involved in SUMO mediated protein control. We have depleted PML in parental and G3BP1/2 knockout cells and analyzed the status of the UPS during the recovery phase. Similar to RNF4 depletion, we found that depletion of PML largely prevented the aggravated UPS impairment in G3BP1/2 knockout cells. This is in line with our model that enhanced targeted of protein for SUMO/ubiquitin-dependent degradation is responsible for the compromised UPS activity in these cells. These data are shown in Fig EV4E and F and are discussed at lines 382-385.

"Does the size of aggregates in nucleoli vary during the recovery from heat shock in G3BP1/2-depleted cells?"

Authors: The localization of nucleolar DRiPs of typically matches the contours of the nucleoli. We have not referred to those structures as "aggregates in nucleoli". From our data it seems localization of DRiPs throughout the nucleoli and, as such, measuring the size will bear little relevance to the dynamics of the DRiPs as they will reflect the size of the nucleoli.

Citations

Anisimov S, Takahashi M, Kakihana T, Katsuragi Y, Kitaura H, Zhang L, Kakita A, Fujii M (2019) G3BP1 inhibits ubiquitinated protein aggregations induced by p62 and USP10. *Sci Rep* **9:** 12896

Kedersha N, Panas MD, Achorn CA, Lyons S, Tisdale S, Hickman T, Thomas M, Lieberman J, McInerney GM, Ivanov P, Anderson P (2016) G3BP-Caprin1-USP10 complexes mediate stress granule condensation and associate with 40S subunits. *J Cell Biol* **212:** 845-860

Meyer C, Garzia A, Morozov P, Molina H, Tuschl T (2020) The G3BP1-Family-USP10 Deubiquitinase Complex Rescues Ubiquitinated 40S Subunits of Ribosomes Stalled in Translation from Lysosomal Degradation. *Mol Cell* **77:** 1193-1205 e1195

Panas MD, Schulte T, Thaa B, Sandalova T, Kedersha N, Achour A, McInerney GM (2015) Viral and cellular proteins containing FGDF motifs bind G3BP to block stress granule formation. *PLoS Pathog* **11:** e1004659

Soncini C, Berdo I, Draetta G (2001) Ras-GAP SH3 domain binding protein (G3BP) is a modulator of USP10, a novel human ubiquitin specific protease. *Oncogene* **20:** 3869-3879

1st Revision - Editorial Decision 21st Nov 2022

Thank you for submitting your revised manuscript, and apologies for the delay with its external and editorial re-evaluation. The study has been seen once more by the original referees, and I am happy to say that all three were fully satisfied with the revisions. As soon as a few remaining editorial points listed below have been addressed, we should therefore be ready to accept the manuscript for publication.

Referee #1:

The authors have convincingly addressed all concerns and I support the publication of this manuscript.

Referee #2:

In the revised manuscript the authors show that G3BP1/2 knockout does not influence the proteolytic activity and the localization of proteasomes, nor the ubiquitination status of DRiPs. These findings are consistent with the proposed model of proteasomal overload rather than UPS dysfunction. The new analyses significantly improved the manuscript, and the authors have addressed my most relevant concerns. Although some mechanistic aspects are still unclear, the authors compile a number of relevant observations showing how loss of stress granules impinges on nuclear proteostasis.

Referee #3:

I believe the current version of the manuscript is suitable for publication in EMBO J.

2nd Revision - Editorial Decision 1st Dec 2022

Thank you for submitting your final revised manuscript for our consideration. I am pleased to inform you that we have now accepted it for publication in The EMBO Journal.

EMBO Press Author Checklist

Corresponding Author Name: Nico Dantuma	
Journal Submitted to: Hartmut Vodermaier	
Manuscript Number: EMBOJ-2022-111802	

USEFUL LINKS FOR COMPLETING THIS FORM
The EMBO Journal - Author Guidelines
EMBO Reports - Author Guidelines
Molecular Systems Biology - Author Guidelines
EMBO Molecular Medicine - Author Guidelines

Reporting Checklist for Life Science Articles (updated January
This checklist is adapted from Materials Design Analysis Reporting (MDAR) Checklist for Authors. MDAR establishes a minimum set of requirements in transparent reporting in the life sciences (see Statement of Task: 10.31222/osf.io/9sm4x). Please follow the journal's guidelines in preparing your manuscript.
Please note that a copy of this checklist will be published alongside your article.

Abridged guidelines for figures
1. Data
The data shown in figures should satisfy the following conditions:
- the data were obtained and processed according to the field's best practice and are presented to reflect the results of the experiments in an accurate and unbiased manner.
- ideally, figure panels should include only measurements that are directly comparable to each other and obtained with the same assay.
- plots include clearly labeled error bars for independent experiments and sample sizes. Unless justified, error bars should not be shown for technical
- if n<5, the individual data points from each experiment should be plotted. Any statistical test employed should be justified.
- Source Data should be included to report the data underlying figures according to the guidelines set out in the authorship guidelines on Data

2. Captions
Each figure caption should contain the following information, for each panel where they are relevant:
- a specification of the experimental system investigated (eg cell line, species name).
- the assay(s) and method(s) used to carry out the reported observations and measurements.
- an explicit mention of the biological and chemical entity(ies) that are being measured.
- an explicit mention of the biological and chemical entity(ies) that are altered/varied/perturbed in a controlled manner.
- the exact sample size (n) for each experimental group/condition, given as a number, not a range;
- a description of the sample collection allowing the reader to understand whether the samples represent technical or biological replicates (including how many animals, litters, cultures, etc.).
- a statement of how many times the experiment shown was independently replicated in the laboratory.
- definitions of statistical methods and measures:
  - common tests, such as t-test (please specify whether paired vs. unpaired), simple χ2 tests, Wilcoxon and Mann-Whitney tests, can be unambiguously identified by name only, but more complex techniques should be described in the methods section;
  - are tests one-sided or two-sided?
  - are there adjustments for multiple comparisons?
  - exact statistical test results, e.g., P values = x but not P values < x;
  - definition of 'center values' as median or average;
  - definition of error bars as s.d. or s.e.m.

Please complete ALL of the questions below.
Select "Not Applicable" only when the requested information is not relevant for your study.

Materials

Newly Created Materials	Information included in the manuscript?	In which section is the information available? (Reagents and Tools Table, Materials and Methods, Figures, Data Availability Section)
New materials and reagents need to be available; do any restrictions apply?	Not Applicable	

Antibodies	Information included in the manuscript?	In which section is the information available? (Reagents and Tools Table, Materials and Methods, Figures, Data Availability Section)
For **antibodies** provide the following information:<br>- Commercial antibodies: RRID (if possible) or supplier name, catalogue number and or/clone number<br>- Non-commercial: RRID or citation	Yes	Material and Methods

DNA and RNA sequences	Information included in the manuscript?	In which section is the information available? (Reagents and Tools Table, Materials and Methods, Figures, Data Availability Section)
**Short novel DNA or RNA including primers, probes**: provide the sequences.	Yes	Material and Methods

Cell materials	Information included in the manuscript?	In which section is the information available? (Reagents and Tools Table, Materials and Methods, Figures, Data Availability Section)
**Cell lines:** Provide species information, strain. Provide accession number in repository **OR** supplier name, catalog number, clone number, and/**OR** RRID.	Yes	Materials and Methods
**Primary cultures:** Provide species, strain, sex of origin, genetic modification status.	Not Applicable	
Report if the cell lines were recently **authenticated** (e.g., by STR profiling) and tested for mycoplasma contamination.	Yes	In Materials and Methods is mentioned that our cell lines are routinely tested for mycoplasma. The cell lines have not recently been authenticated.

Experimental animals	Information included in the manuscript?	In which section is the information available? (Reagents and Tools Table, Materials and Methods, Figures, Data Availability Section)
**Laboratory animals or Model organisms:** Provide species, strain, sex, age, genetic modification status. Provide accession number in repository **OR** supplier name, catalog number, clone number, **OR** RRID.	Not Applicable	
**Animal observed in or captured from the field:** Provide species, sex, and age where possible**.**	Not Applicable	
Please detail **housing and husbandry conditions**.	Not Applicable	

Plants and microbes	Information included in the manuscript?	In which section is the information available? (Reagents and Tools Table, Materials and Methods, Figures, Data Availability Section)
**Plants:** provide species and strain, ecotype and cultivar where relevant, unique accession number if available, and source (including location for collected wild specimens).	Not Applicable	
**Microbes:** provide species and strain, unique accession number if available, and source.	Not Applicable	

Human research participants	Information included in the manuscript?	In which section is the information available? (Reagents and Tools Table, Materials and Methods, Figures, Data Availability Section)
If collected and within the bounds of privacy constraints report on age, sex and gender or ethnicity for all study participants.	Not Applicable	

Core facilities	Information included in the manuscript?	In which section is the information available? (Reagents and Tools Table, Materials and Methods, Figures, Data Availability Section)
If your work benefited from core facilities, was their service mentioned in the acknowledgements section?	Yes	Acknowledgements

Design

Study protocol	Information included in the manuscript?	In which section is the information available? (Reagents and Tools Table, Materials and Methods, Figures, Data Availability Section)
If study protocol has been **pre-registered, provide DOI in the manuscript**. For clinical trials, provide the trial registration number **OR** cite DOI.	Not Applicable	
Report the **clinical trial registration number** (at ClinicalTrials.gov or equivalent), where applicable.	Not Applicable	

Laboratory protocol	Information included in the manuscript?	In which section is the information available? (Reagents and Tools Table, Materials and Methods, Figures, Data Availability Section)
Provide DOI OR other citation details if **external detailed step-by-step protocols** are available.	Not Applicable	

Experimental study design and statistics	Information included in the manuscript?	In which section is the information available? (Reagents and Tools Table, Materials and Methods, Figures, Data Availability Section)
Include a statement about **sample size** estimate even if no statistical methods were used.	Yes	
Were any steps taken to minimize the effects of subjective bias when allocating animals/samples to treatment (e.g. **randomization procedure**)? If yes, have they been described?	Not Applicable	
Include a statement about **blinding** even if no blinding was done.	Not Applicable	
Describe **inclusion/exclusion criteria** if samples or animals were excluded from the analysis. Were the criteria pre-established?<br><br>If sample or data points were omitted from analysis, report if this was due to attrition or intentional exclusion and provide justification.	Not Applicable	
For every figure, are **statistical tests** justified as appropriate? Do the data meet the assumptions of the tests (e.g., normal distribution)? Describe any methods used to assess it. Is there an estimate of variation within each group of data? Is the variance similar between the groups that are being statistically compared?	Yes	Material and Methods

Sample definition and in-laboratory replication	Information included in the manuscript?	In which section is the information available? (Reagents and Tools Table, Materials and Methods, Figures, Data Availability Section)
In the figure legends: state number of times the experiment was **replicated** in laboratory.	Yes	Figure legends
In the figure legends: define whether data describe **technical or biological replicates**.	Yes	In figure legends we specify if the data are from independent experiements and/or the number of cells per sample.

Ethics

Ethics	Information included in the manuscript?	In which section is the information available? (Reagents and Tools Table, Materials and Methods, Figures, Data Availability Section)
Studies involving **human participants**: State details of **authority granting ethics approval** (IRB or equivalent committee(s), provide reference number for approval.	Not Applicable	
Studies involving **human participants**: Include a statement confirming that **informed consent** was obtained from all subjects and that the experiments conformed to the principles set out in the WMA Declaration of Helsinki and the Department of Health and Human Services Belmont Report.	Not Applicable	
Studies involving **human participants:** For publication of **patient photos**, include a statement confirming that consent to publish was obtained.	Not Applicable	
Studies involving experimental **animals**: State details of **authority granting ethics approva**l (IRB or equivalent committee(s), provide reference number for approval. Include a statement of compliance with ethical regulations.	Not Applicable	
Studies involving **specimen and field samples:** State if relevant **permits** obtained, provide details of authority approving study; if none were required, explain why.	Not Applicable	

Dual Use Research of Concern (DURC)	Information included in the manuscript?	In which section is the information available? (Reagents and Tools Table, Materials and Methods, Figures, Data Availability Section)
Could your study fall under dual use research restrictions? Please check biosecurity documents and list of **select agents and toxins** (CDC): https://www.selectagents.gov/sat/list.htm	Not Applicable	
If you used a select agent, is the security level of the lab appropriate and reported in the manuscript?	Not Applicable	
If a study is subject to dual use research of concern regulations, is the name of the **authority granting approval and reference number** for the regulatory approval provided in the manuscript?	Not Applicable	

Reporting

The MDAR framework recommends adoption of discipline-specific guidelines, established and endorsed through community initiatives. Journals have their own policy about requiring specific guidelines and recommendations to complement MDAR.

Adherence to community standards	Information included in the manuscript?	In which section is the information available? (Reagents and Tools Table, Materials and Methods, Figures, Data Availability Section)
State if relevant guidelines or checklists (e.g., **ICMJE, MIBBI, ARRIVE, PRISMA**) have been followed or provided.	Not Applicable	
For **tumor marker prognostic studies,** we recommend that you follow the **REMARK** reporting guidelines (see link list at top right). See author guidelines, under 'Reporting Guidelines'. Please confirm you have followed these guidelines.	Not Applicable	
For **phase II and III randomized controlled trials**, please refer to the **CONSORT** flow diagram (see link list at top right) and submit the CONSORT checklist (see link list at top right) with your submission. See author guidelines, under 'Reporting Guidelines'. Please confirm you have submitted this list.	Not Applicable	

Data Availability

Data availability	Information included in the manuscript?	In which section is the information available? (Reagents and Tools Table, Materials and Methods, Figures, Data Availability Section)
Have **primary datasets** been deposited according to the journal's guidelines (see 'Data Deposition' section) and the respective accession numbers provided in the Data Availability Section?	Not Applicable	
Were **human clinical and genomic datasets** deposited in a public access-controlled repository in accordance to ethical obligations to the patients and to the applicable consent agreement?	Not Applicable	
Are **computational models** that are central and integral to a study available without restrictions in a machine-readable form? Were the relevant accession numbers or links provided?	Not Applicable	
If publicly available data were reused, provide the respective **data citations in the reference list**.	Not Applicable	
