# Supplementary material for: Cytosolic stress granules relieve the ubiquitin‐proteasome system in the nuclear compartment
Source: EMBO J. 2022 Dec 27;42(3):e111802. doi: 10.15252/embj.2022111802 (PMC9890234; doi:10.15252/embj.2022111802)
Supplement: Supplementary file 1 — Appendix [file EMBJ-42-e111802-s001.zip › EMBOJ-2022-111802R1_ComputerCodeEV1.zip!/Legend_CCEV1.rtf]

Legend: Computer Code EV1

CellProfiler pipeline for quantification of cellular mean fluorescence intensities
